# Palladium-Catalyzed Synthesis of 2,3-Disubstituted Benzofurans: An Approach Towards the Synthesis of Deuterium Labeled Compounds

**DOI:** 10.1002/adsc.201500308

**Published:** 2015-07-14

**Authors:** Soumitra Agasti, Soham Maity, Kalman J Szabo, Debabrata Maiti

**Affiliations:** a Department of Chemistry, Indian Institute of Technology Bombay Powai, Mumbai – 400076, India E-mail: dmaiti@chem.iitb.ac.in; b Department of Organic Chemistry, Stockholm University SE-106 91 Stockholm, Sweden E-mail: kalman@organ.su.se

**Keywords:** benzofurans, C–H activation, deuterium, palladium, synthetic methods

## Abstract

Palladium-catalyzed oxidative annulations between phenols and alkenylcarboxylic acids produced a library of benzofuran compounds. Depending on the nature of the substitution of the phenol precursor, either 2,3-dialkylbenzofurans or 2-alkyl-3-methylene-2,3-dihydrobenzofurans can be synthesized with excellent regioselectivity. Reactions between conjugated 5-phenylpenta-2,4-dienoic acids and phenol gave 3-alkylidenedihydrobenzofuran alkaloid motifs while biologically active 7-arylbenzofuran derivatives were prepared by starting from 2-phenylphenols. More interestingly, selective incorporation of deuterium from D_2_O has been discovered, which offers an attractive one-step method to access deuterated compounds.

Benzofurans are an important class of heterocyclic compounds[1] with unique biological activities.[2] Notable instances include derivatives of benzofurans acting as antitumor agents,[3] angiotensin II inhibitors,[4] and 5-lipoxygenase inhibitors etc.[5] Therefore, synthesis of these organic motifs has drawn significant attention from the synthetic community.[6] Recently, we have contributed to this area by synthesizing a wide array of 2-substituted benzofurans through a unique Pd-catalyzed annulation of simple phenols and olefins.[7] Of more interest was an orthogonal approach with cinnamic acids, which gave rise to 3-substituted benzofurans with excellent selectivity.[8] In this context, we became interested in the prospect of synthesizing 2,3-disubstituted benzofuran derivatives starting from phenols. Although numerous approaches have been made to synthesize these scaffolds,[9] the widely adopted method is the transition metal-catalyzed annulation[10] by using pre-functionalized phenol,[6a],[11] thus limiting the scope of the reaction to a considerable extent. Free phenols also have been employed in several cases but reactions with cinnamic acids remained exceedingly rare.[12]

In addition, we disclose a one-step method to synthesize deuterium-labeled benzofurans in the presence of D_2_O (Scheme 1). Deuterated compounds are ubiquitous in the realms of metabolic studies, mechanistic experimentations and most importantly in mass spectrometry.[13] Furthermore, deuterium incorporated compounds are found to improve the therapeutic and metabolic profiles of a drug candidate.[14] To the best of our knowledge, the synthesis of deuterated benzofurans from unbiased phenol remains unsolved as yet.

**Scheme 1:**
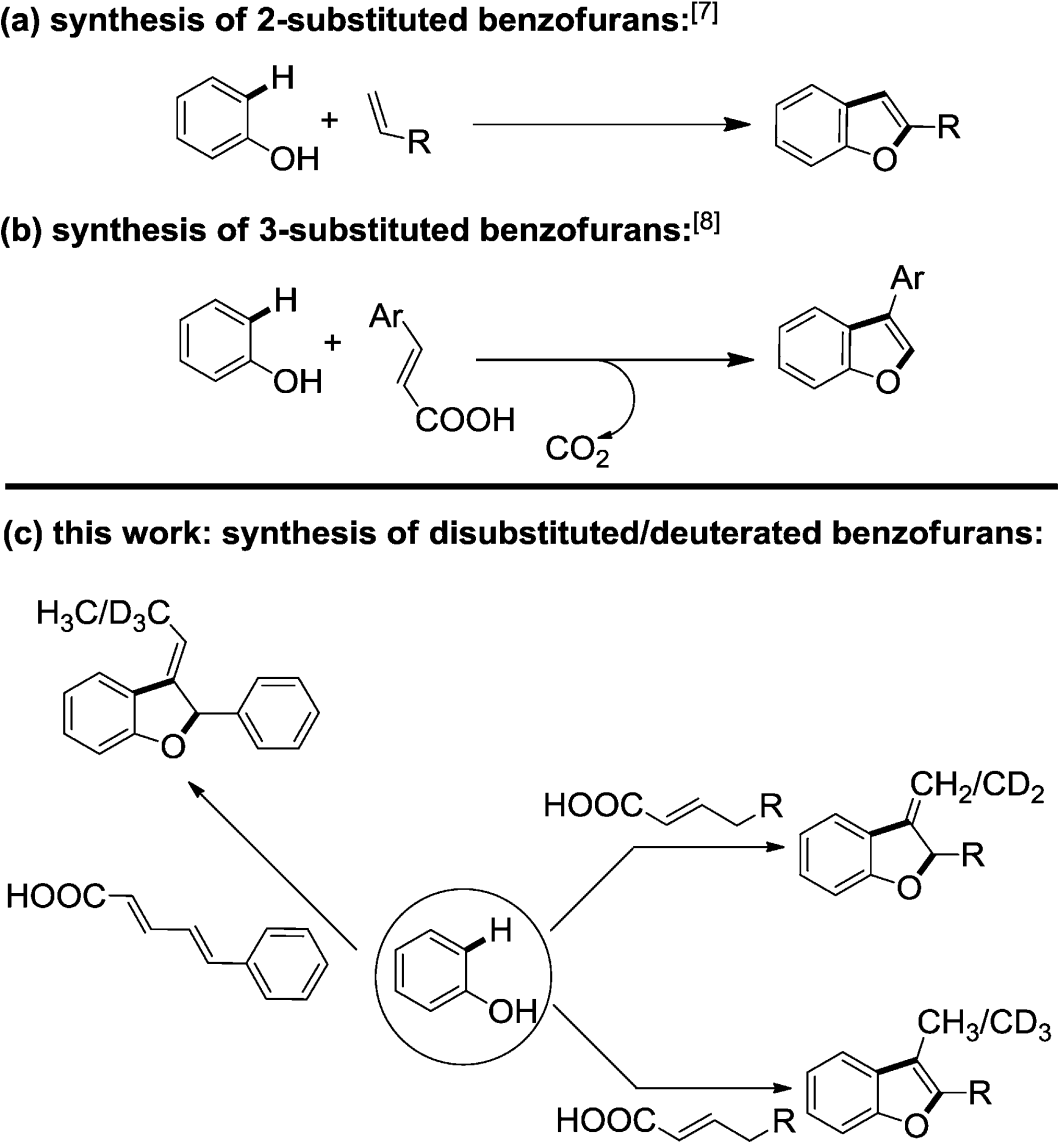
Our approaches to benzofuran synthesis.

At the beginning of our investigation, we hypothesized about an alteration of the reaction mode upon changing the coupling partner from cinnmic acids to α,β-unsaturated aliphatic acids (Scheme 2). This preliminary idea was based on the putative intermediate (**A**) which is less likely to undergo a direct oxopalldation due to the decreased stabilization of the incipient negative charge (monobenzyl *vs.* dibenzyl center). In fact, in the next step an allylpalladium species **B** can be envisaged with the tentative migration of the double bond to the more substituted position, which can generate disubstituted benzofurans as opposed to the 3-substituted ones observed previously.[8] In accordance with this hypothesis, we commenced our initial studies with a reaction of 2-chloro-4-nitrophenol and 8-nonenoic acid with the catalyst Pd/1,10-phen in the presence of Cu(OAc)_2_⋅H_2_O as the terminal oxidant. After several sets of optimization, we found that desired disubstituted benzofurans can be synthesized efficiently in dichloroethane (DCE) solvent at 130 °C.[15]

**Scheme 2:**
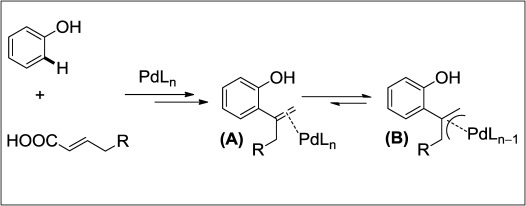
Mechanistic outline.

The scope of the phenol coupling partners was studied subsequently under the optimized reaction condition (**3a–4k**). Depending on the nature of substitution of the phenols, we observed formation of either 3-methylene-2,3-dihydrobenzofuran (**3**) or 2,3-dialkylbenzofuran (**4**) derivatives (Table 1). The 8-nonenoic acid reacted with 4-cyanophenol to produce 3-methylene-2,3-dihydrobenzofuran as the major product (**3b**) along with isomer **4b** in trace amount (**3b/4b**, 10:1). In a similar fashion, 4-nitrophenol reacted with the same olefin to produce **3c** in preparatively useful yields. Such compounds were previously synthesized by ruthenium-carbene promoted cycloisomerization of *O*-allyl-*o*-vinylphenols.[16] In the present case, formation of **3** likely involved a β-migratory insertion and β-hydride elimination pathway (*vide infra*).

**Table 1:** Scope with different phenols and α,β-unsaturated carboxylic acids^[a]^

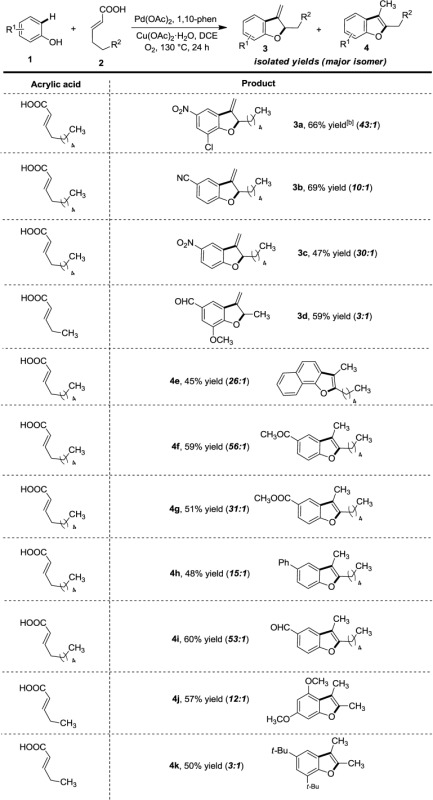

^[a]^ *Reaction conditions:*­ **1** (0.75 mmol, 3 equiv.), **2** (0.25 mmol, 1 equiv.), Pd(OAc)_2_ (0.025 mmol, 10 mol%), 1,10-phenanthroline (0.05 mmol, 20 mol%), Cu(OAc)_2_ (0.25 mmol, 1 equiv.), ClCH_2_CH_2_Cl (4 mL), 130 °C for 24 h in an O_2_ atm. Yields are those of the isolated major products. Compound ratio was determined on the basis of GC-MS analysis of the reaction mixture. Compound **3:4** ratio was mentioned for entries **3a**–**3d** and compound **4:3** ratio was mentioned for entries **4e**–**4k**. The minor product could not be isolated in pure form.

^[b]^ Compound was characterized by 1D and 2D NMR.

Relatively less electron-deficient phenols were also found to be suitable under the present system. A keto-substituted phenol could produce 3-methyl-substituted **4f** as the major product along with the exocyclic isomer in an negligible amount (**4f**/**3f**, 56:1). Similar products were also observed in **4g**–**4k**. Despite our best efforts, the preference for **3**­ *vs.*­ **4** (Table 1) cannot be rationalized at this point. We speculated that a subtle difference in electronic nature of phenols (e.g., strongly electron-deficient phenols gave **3**) is crucial for these product formations. Although **3** is known to isomerize to the corresponding 3-methyl-2,3-disubstituted benzofuran (**4**) under acidic conditions,[17] we failed to promote such a transformation in our laboratory (e.g., with **3a**). In addition to the synthesis of benzofurans, naphthofurans (e.g., **4e**; **4e**/**3e**, 26:1) can also be synthesized, which are integral components in natural products and pharmacologically relevant compounds.[18] Expectedly, electron-rich phenols reacted with 4-pentenoic acid to produce 2,3-dimethyl-substituted benzofuran compounds **4j** and **4k** with useful synthetic yields.

Subsequently, we planned to synthesize 7-arylbenzofuran derivatives which are present in a number of natural products.[19] Note that the synthesis of 7-arylbenzofuran from simple precursors remained problematic up to date (Scheme 3).

**Scheme 3:**
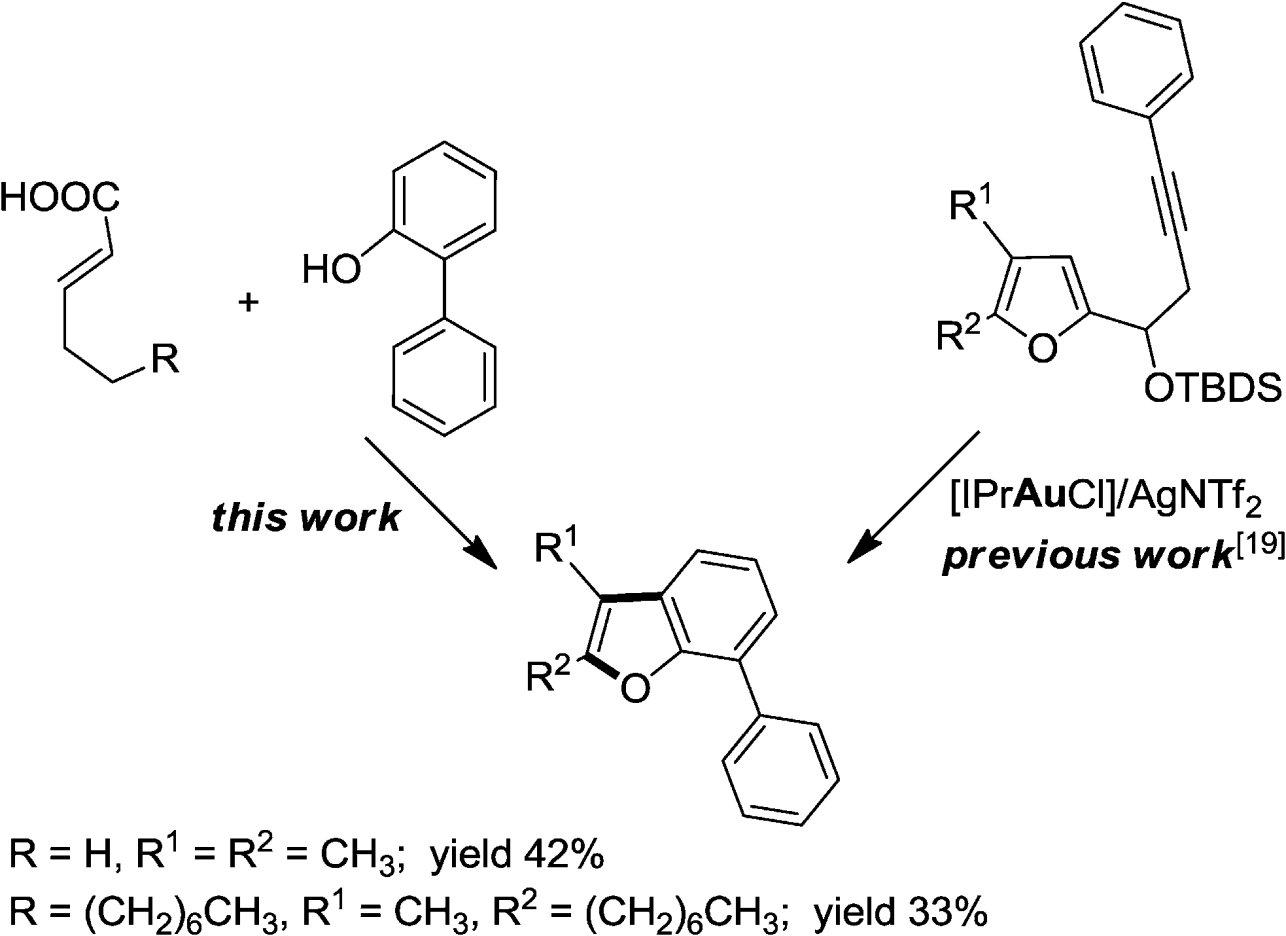
Synthesis of 2,3-disubstituted-7-arylbenzofurans.

Next, the scope of the present method was expanded to 3-alkylidenedihydrobenzofuran derivatives, which are very relevant to alkaloid chemistry, by reacting conjugated 5-phenylpenta-2,4-dienoic acid with phenol.[1b],[20] Ylide hydrolysis and intramolecular cyclization were previously explored to synthesize these 3-alkylidenedihydrobenzofuran compounds.[21] However, under the present conditions, an array of 3-alkylidenedihydrobenzofuran derivatives (**6**) could be synthesized in a much simpler way in good yields (Table 2).

**Table 2:** Scope with conjugated α,β-unsaturated carboxylic acids^[a]^

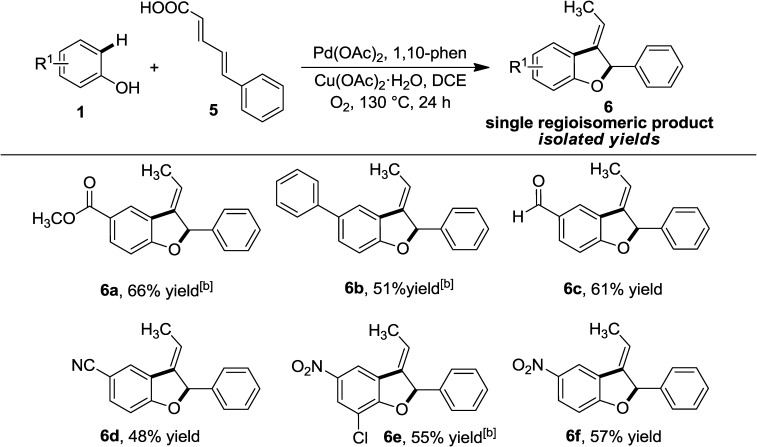

^[a]^ *Reaction conditions:*­ **1** (0.75 mmol, 3 equiv.), **2** (0.25 mmol, 1 equiv.), Pd(OAc)_2_ (0.025 mmol, 10 mol%), 1,10-phenanthroline (0.05 mmol, 20 mol%), Cu(OAc)_2_ (0.25 mmol, 1 equiv.), ClCH_2_CH_2_Cl (4 mL), 130 °C for 24 h in an O_2_ atm. Yields are those of the isolated products. Compounds were characterized by 1D and 2D NMR.

^[b]^ Bathophenanthroline as the ligand.

In view of the lability of the carboxylic proton, deuterium-exchange was planned with the addition of D_2_O under standard reaction conditions. After a brief optimization effort we found that 500 μL D_2_O are sufficient to obtain the maximum percentage of deuterium incorporation.[15] Employing the present approach, an array of deuterated benzofuran analogues were accessed in one step (Scheme 4). Furthermore, deuterated 3-methylene-2,3-dihydrobenzofuran derivatives were also synthesized in a similar fashion. The 8-nonenoic acid in the presence of the electron -withdrawing partner like 2-chloro-4-nitrophenol (**3′a**) and 4-cyanophenol (**3′b**) provided the desired benzofuran products in 65% and 47% yields, respectively. Substitution on the phenol coupling partner like *t-*Bu and Ph gave the expected 2,3-disubstituted benzofurans, where a –CD_3_ group is present on the 3-position (**4′a** and **4′b**). Dimethoxy-substituted phenol resulted in non-selective over deuteration (**4′c**) due to the acidic nature of protons present in the *ortho*-position of the methoxy group. Next, we synthesized deuterated 3-alkylidenedihydrobenzofuran derivatives from conjugated 5-phenylpenta-2,4-dienoic acid and phenol with synthetically useful yields (**6′a**–**6′c**). Note that, by using PhOH-*d*_5_ as coupling partner in the absence of D_2_O, we did not observe any deuterium scrambling (Scheme 5).

**Scheme 4:**
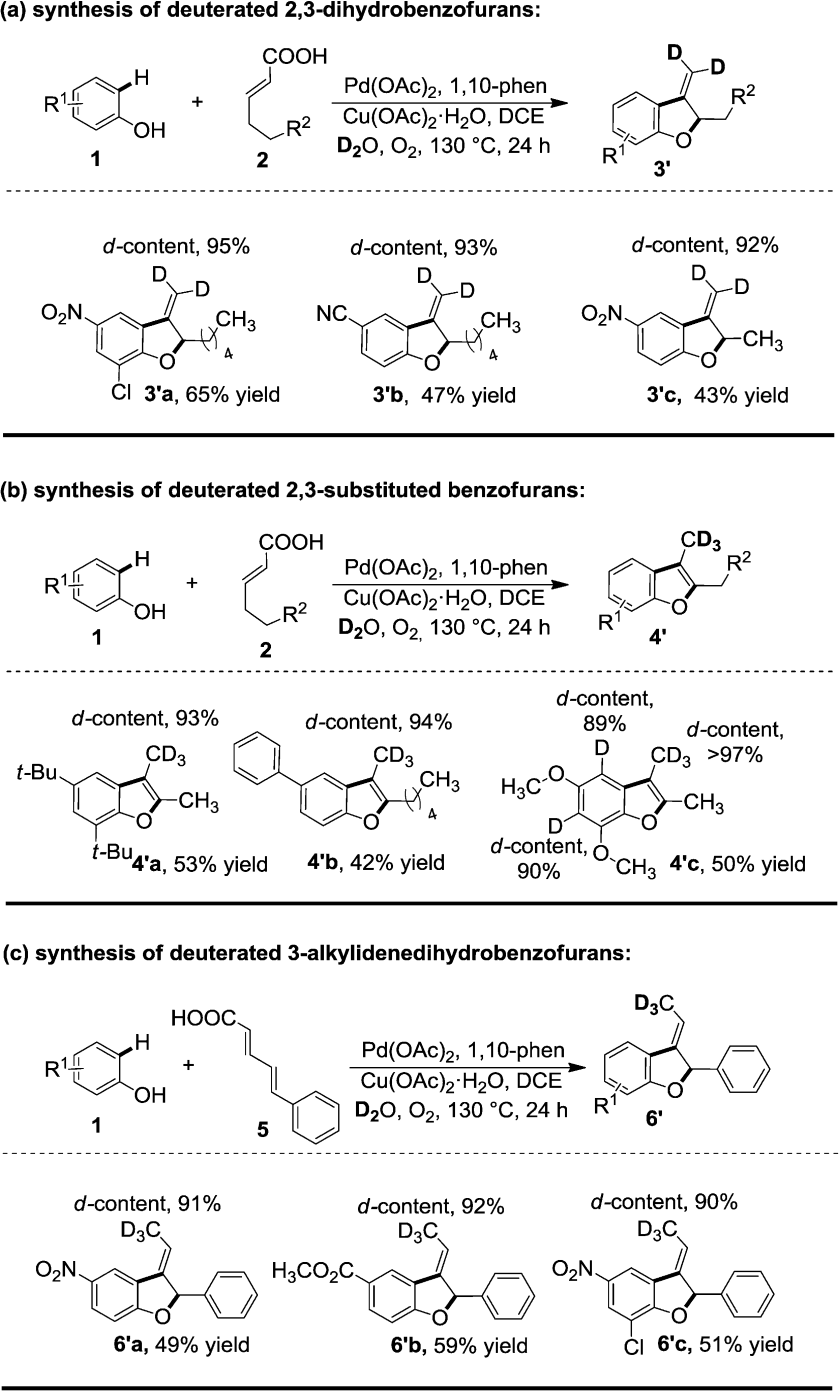
D_2_O addition under standard conditions. *Reaction conditions:*­ 1 (0.75 mmol, 3 equiv.), 2 (0.25 mmol, 1 equiv.), Pd(OAc)_2_ (0.025 mmol, 10 mol%), 1,10-phenanthroline (0.05 mmol, 20 mol%), Cu(OAc)_2_ (0.25 mmol, 1 equiv.), D_2_O (500 μL), ClCH_2_CH_2_Cl (4 mL), 130 °C for 24 h in an O_2_ atm. Yields are those of the isolated products.

**Scheme 5:**
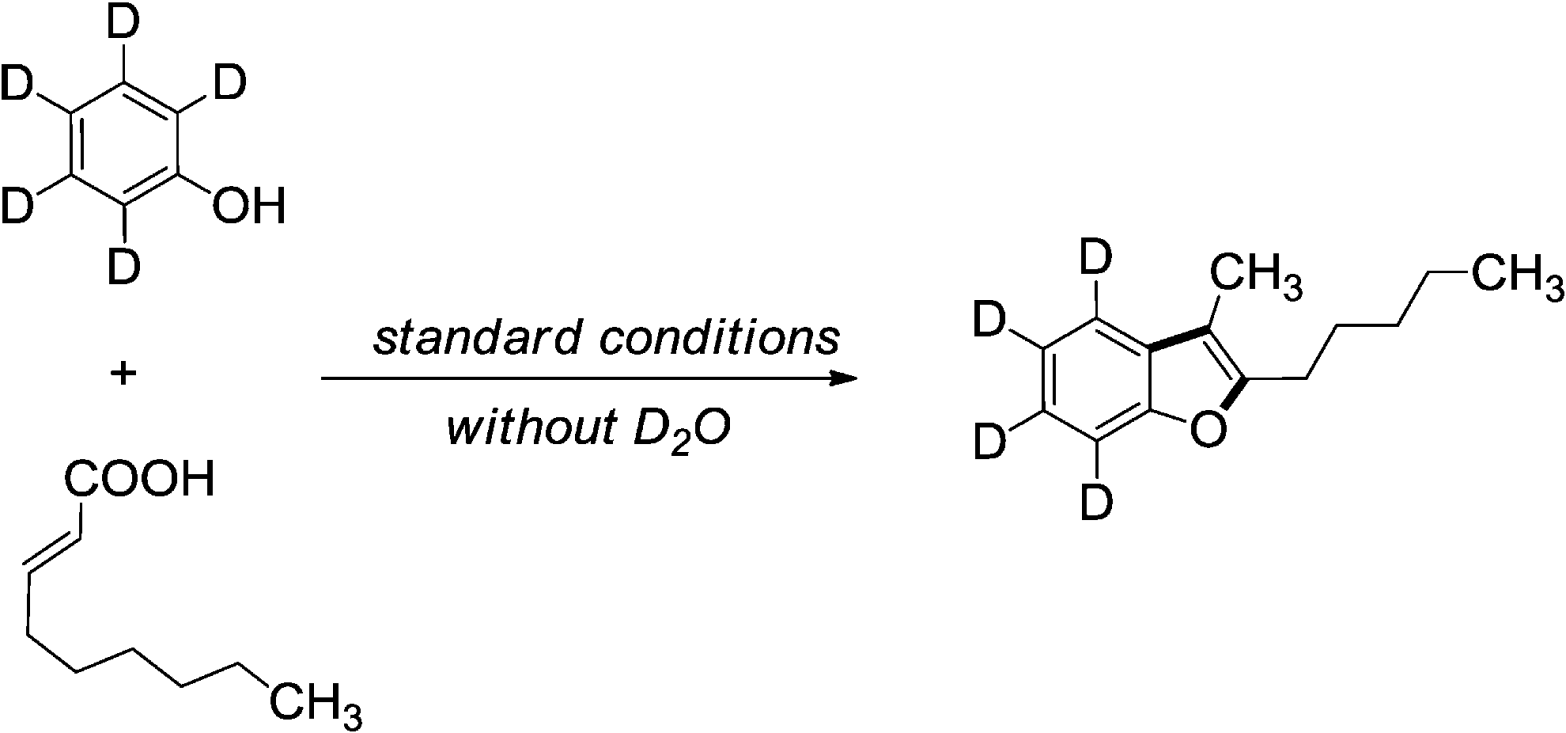
Complementary isotope labeling study using deuterated phenol.

Based on the experimental observations, a plausible mechanism of 3-methylene-2,3-dihydrobenzofuran and 2,3-disubstituted benzofuran synthesis is depicted in Scheme 6. Formation of a phenanthroline-palladium(II) complex increases the solubility and electrophilicity of the resulting catalyst. We tentatively speculated that the electrophilic palladium center will coordinate to the *ortho*-position of the phenol to give a palladium-phenolic complex.[12e],[22] Then α,β-unsaturated carboxylic acids will be inserted across the C–Pd bond and subsequent decarboxylation will give the Pd-allyl species (**Int-I**).[8],[23] In the presence of palladium, intermediate **I** will cyclize to form **Int**-**II**, which is the key species for the formation of **3** and **4**.[1b],[7],[11c] Syn β-hydride elimination from intermediate **II** leads to the formation of the desired benzofuran products and regenerates the Pd(0) species.[7] This Pd(0) is readily oxidised to Pd(II) by Cu(OAc)_2_⋅H_2_O under an oxygen atmosphere to maintain the catalytic process.

**Scheme 6:**
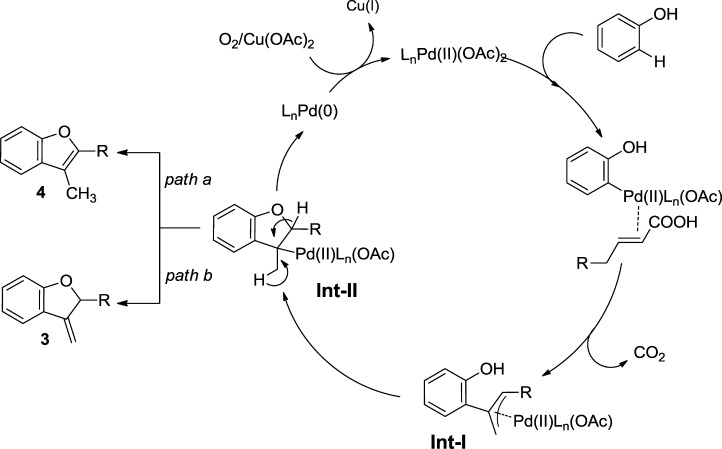
Formation of 3 and 4.

A reasonable pathway to obtain **3′** and **4′** (Scheme 4) *via* deuterium incorporation, β-migratory insertion and β-hydride elimination can also be envisaged (Scheme 7).

**Scheme 7:**
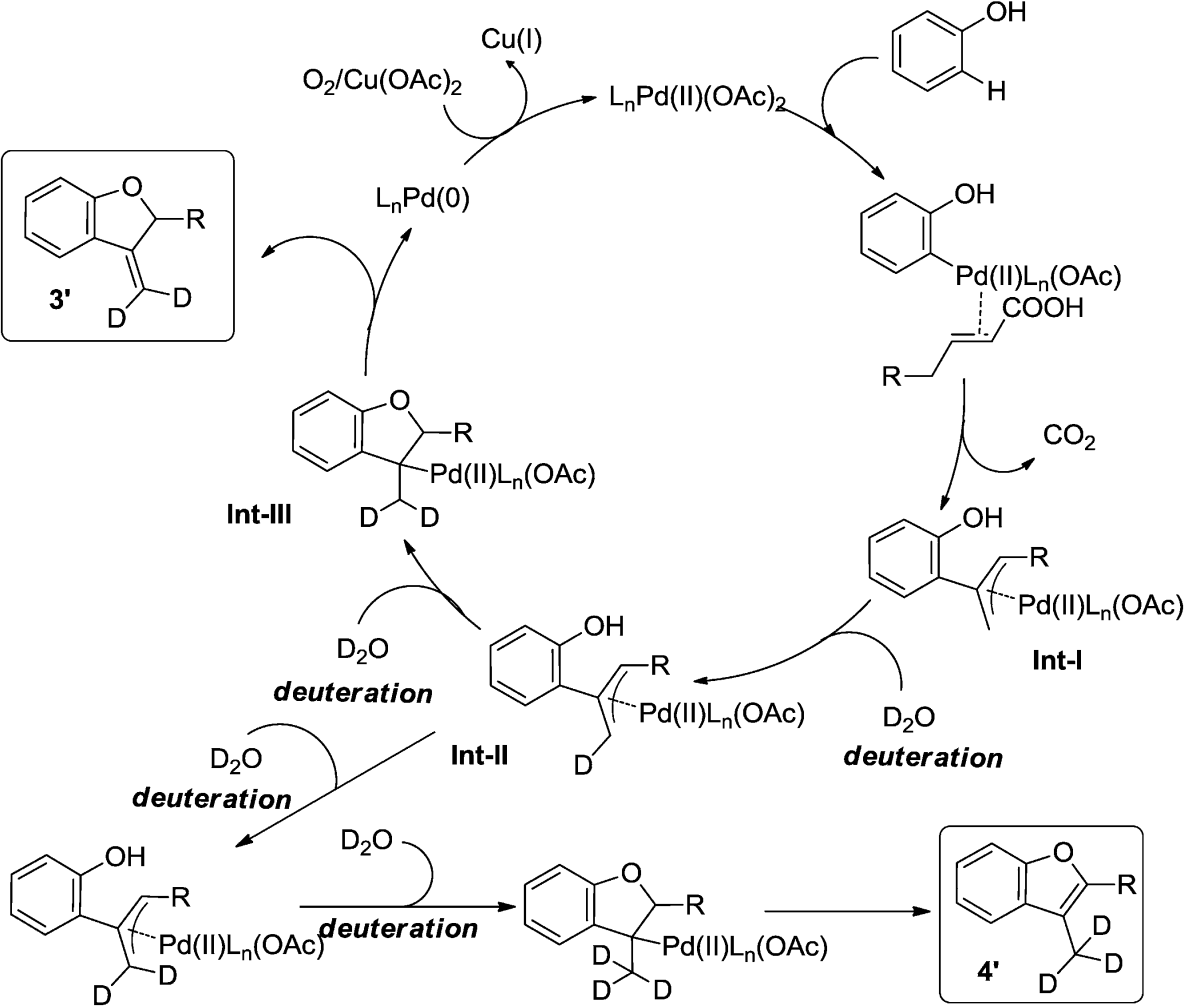
Formation of 3′ and 4′.

In summary, 2, 3-disubstituted benzofuran analogues are synthesized from readily available phenols and aliphatic α,β-unsaturated carboxylic acids. Excellent regioselectivity and use of inexpensive reagents make this method synthetically useful. An inverse insertion with α,β-unsaturated carboxylic acids compared to alkenes, was observed upon *ortho*-palladation of phenol. Additionally, this method can be utilized for the preparation of deuterated benzofuran compounds. Further mechanistic investigations and expansion of such strategies are currently underway in our laboratory.

## Experimental Section

### General Procedure

To an oven-dried screw cap reaction tube charged with a magnetic stir-bar, Pd(OAc)_2_ (10 mol%, 0.025 mmol, 5.6 mg), 1,10-phenonthroline monohydrate (20 mol%, 0.05 mmol, 10 mg) or bathophenanthroline (20 mol%, 0.05 mmol, 16.62 mg), Cu(OAc)_2_⋅H_2_O (0.25 mmol, 50 mg) were added. Then phenol (0.75 mmol) and α,β-unsaturated carboxylic acid (0.25 mmol) were introduced into the reaction mixture. Solid compounds were weighed before the other reagents, whereas liquid phenols/α,β-unsaturated carboxylic acids were added by micro-liter syringe and laboratory syringe under an air atmosphere. In the reaction tube 4 mL ClCH_2_CH_2_Cl were added and O_2_ was purged in the reaction mixture for 15 min. For the deuterated compounds (**3′a**–**6′c**), 500 μL D_2_O were added by micro-liter syringe under the positive pressure of oxygen. Then the reaction mixture was vigorously stirred in a preheated oil bath at 130 °C for 24 h. After completion, the reaction mixture was filtered through a celite pad with ethyl acetate as the washing solvent. The ethyl acetate layer was washed with brine solution and dried over anhydrous Na_2_SO_4_, and evaporated under reduced pressure. The residue was purified by column chromatography.

## Supporting Information

As a service to our authors and readers, this journal provides supporting information supplied by the authors. Such materials are peer reviewed and may be re-organized for online delivery, but are not copy-edited or typeset. Technical support issues arising from supporting information (other than missing files) should be addressed to the authors.

miscellaneous_information
