# Supplementary material for: Palladium-Catalyzed Synthesis of 2,3-Disubstituted Benzofurans: An Approach Towards the Synthesis of Deuterium Labeled Compounds
Source: Adv Synth Catal. 2015 Jul 14;357(10):2331–8. doi: 10.1002/adsc.201500308 (PMC4552971; doi:10.1002/adsc.201500308)
Supplement: Supplementary file 1 — miscellaneous_information [file adsc0357-2331-sd1.pdf]

Advanced Synthesis & Catalysis

Supporting Information

Palladium-Catalyzed Synthesis of 2,3-disubstituted Benzofurans: An Approach Towards the Synthesis of Deuterium Labeled Compounds

SoumitraAgasti,[a] SohamMaity,[a] Kalman J. Szabo,[b,*] and Debabrata Maiti[a,*]

[a]Department of Chemistry, Indian Institute of Technology Bombay, Powai, Mumbai 400076, India

[b]Department of Organic Chemistry, Stockholm University, SE-106 91 Stockholm, Sweden

Supporting Information

Table of Contents

General Consideration………………… **S2**

Optimization Detail…………………… **S3**

General Synthesis Procedure………… **S4**

Characterization Data………………… **S5-S20**

Mechanistic details…………………… **S21-S22**

References………………………………….. **S23**

NMR File………………………………. **S24-S78**

General Consideration:

Reagent Information: Unless otherwise stated, all reactions were carried out under oxygen (O_2) atmosphere in screw cap reaction tubes. All the solvents were bought from Aldrich in sure-seal bottle and were used as received. Palladium acetate was obtained as generous gift from Johnson Matthey. All the α,β-unsaturated carboxylic acids and phenols were bought from Aldrich and Alfa-Aesar. For column chromatography, silica gel (100–200 mesh) from SRL Co. was used. A gradient elution using pet ether and ethyl acetate was performed based on Merck aluminium TLC sheets (silica gel $60F_{254}$).

Analytical Information: All isolated compounds are characterized by ^{1}H NMR, ^{13}C NMR spectroscopy, gas chromatography mass spectra (GC-MS). In addition, new compounds are further characterized by HRMS. Copies of the ^{1}H NMR, ^{13}C NMR can be found in the supporting information. Nuclear magnetic resonance spectra were recorded on Bruker 400 MHz, 500 MHz or Varian 400 MHz instrument. All ^{1}H NMR experiments are reported in units, parts per million (ppm), and were measured relative to the signals for residual chloroform (7.26 ppm) in the deuterated solvent, unless otherwise stated. All ^{13}C NMR spectra were reported in ppm relative to deuteron chloroform (77.23 ppm), unless otherwise stated, and all were obtained with ^{1}H decoupling. All GC analyses were performed on Agilent 7890A GC system with an FID detector using a J & W DB–1 column (10 m, 0.1 mm I.D.) with *n*-decane as the internal standard. All GCMS analysis was done by Agilent 7890A GC system connected with 5975C inert XL EI/CI MSD (with triple axis detector).

Optimization Details:[1]

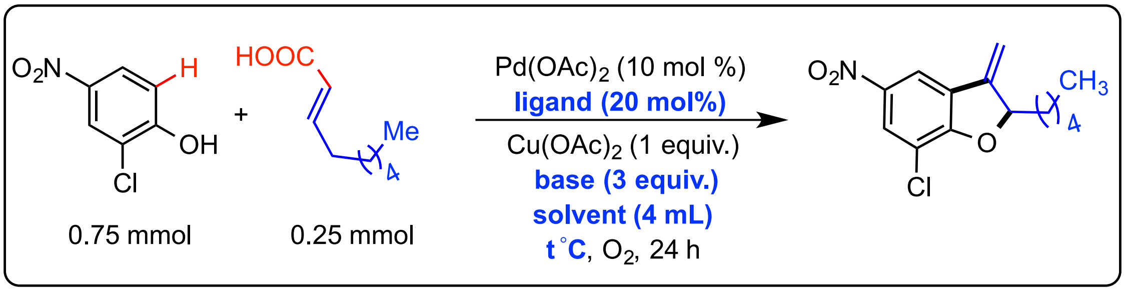

Entry	Ligand	Base	Solvent	Temperature(°C)	Yield (%)
1	1,10-phenanthroline	NaOAc	DCE	110	32
2	1,10-phenanthroline	NaOAc	trichloropropane	110	25
3	1,10-phenanthroline	NaOAc	DCE	120	37
4	1,10-phenanthroline	NaOAc	DCE	130	37
5	1,10-phenanthroline	NaOAc	DCE	140	39
6	bathophenanthroline	NaOAc	DCE	130	35
7	bathophenanthroline	KOAc	DCE	130	33
8	bathophenanthroline	KO^tBu	DCE	130	26
9	bathophenanthroline	NaO^tBu	DCE	130	21
10	1,10-phenanthroline	KOAc	DCE	130	29
**11**	**1,10-phenanthroline**	**---**	**DCE**	**130**	**69**
12	1,10-phenanthroline	---	DCE	140	69
**13**	**bathophenanthroline**	**---**	**DCE**	**130**	**70**
14	1,10-phenanthroline	---	DCE+D_2O(200 μL)	130	69(30)[a]
15	1,10-phenanthroline	---	DCE+D_2O(400 μL)	130	68(70)[a]
**16**	**1,10-phenanthroline**	**---**	**DCE+D_2O(500 μL)**	**130**	**68(95)[a]**

[a]Deuterium incorporation in parenthesis

Pd-Catalyzed Benzofuran Synthesis from Corresponding Phenols and α,β-unsaturated carboxylic acids (General Procedure A): To an oven-dried screw cap reaction tube charged with a magnetic stir-bar, $Pd(OAc)_2$ (10 mol%, 0.025 mmol, 5.6 mg), 1,10-phenonthroline monohydrate (20 mol%, 0.05 mmol, 10 mg) or bathophenanthroline (20 mol%, 0.05mmol, 16.62 mg)[b], $Cu(OAc)_2.H_2O$ (0.25 mmol, 50 mg) were added. Then phenol (0.75mmol) and α,β-unsaturated carboxylic acid (0.25 mmol) were introduced in the reaction mixture. Solid compounds were weighed before the other reagents, whereas liquid phenols/ α,β-unsaturated carboxylic acids were added by micro-litre syringe or laboratory syringe under air atmosphere. In the reaction tube 4 mL DCE ($ClCH_2CH_2Cl$) was added and O_2 was purged in the reaction mixture for 15 min. Then the reaction mixture was vigorously stirred (900 rpm on Heidolph MR Hei-Standard stirrer) in a preheated oil bath at 130 ºC for 24h. After completion, reaction mixture was filtered through a celite pad with ethylacetate as the washing solvent. The ethylacetate layer was washed with brine solution and dried over anhydrous Na_2SO_4, and evaporated under reduced pressure. The residue was purified by column chromatography using silica gel (100-200 mesh size) and petroleum-ether/ ethyl acetate as the eluent.

Deuterated Benzofuran Synthesis (General Procedure B): To an oven-dried screw cap reaction tube charged with a magnetic stir-bar, $Pd(OAc)_2$ (10 mol%, 0.025 mmol, 5.6 mg),1,10-phenonthroline monohydrate (20 mol%, 0.05 mmol, 10 mg)bathophenanthroline (20 mol%, 0.05mmol, 16.62 mg)[b], $Cu(OAc)_2.H_2O$ (0.25 mmol, 50 mg) were added. Then Phenol (0.75 mmol), α,β-unsaturated carboxylic acid (0.25 mmol) wereintroducedin the reaction tube. Liquid reagents were added by micro-litre syringe or laboratory syringe at the end under air atmosphere. In the reaction tube 4 mL DCE ($ClCH_2CH_2Cl$) was added and O_2 was purged in the reaction mixture for 15 min. Then 500 μL D_2O was added by micro-litre syringe in sealed condition under positive pressure of O_2. Then the reaction mixture was vigorously stirred (900 rpm on Heidolph MR Hei-Standard stirrer) in a preheated oil bath at 130 ^oC for 24h. After completion, reaction mixture was filtered through a celite pad with ethylacetate as the washing solvent. The ethylacetate layer was washed with brine solution and dried over anhydrous Na_2SO_4, and evaporated under reduced pressure. The residue was purified by column chromatography using silica gel (100-200 mesh size) and petroleum-ether / ethyl acetate as the eluent.

Characterization data:

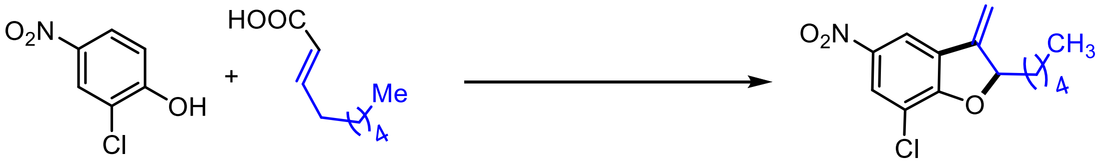

7-chloro-2-ethyl-3-methylene-5-nitro-2,3-dihydrobenzofuran (Table 1; entry 3a) was synthesized by general procedure A. Desired product was obtained as yellow solid in 66% yield after column chromatography of the crude reaction mixture (silica gel, mesh 100-200; petroleum ether)

^{1}H NMR (400 MHz, $CDCl_3$) δ: 0.82 – 0.96 (m, 3H), 1.30 – 1.61 (m, 6H), 1.75 – 1.96 (m, 2H), 5.14 (dd, J = 2.6, 1.2 Hz, 1H), 5.45 (ddt, J = 7.1, 4.0, 2.8 Hz, 1H), 5.64 (dd, J = 3.1, 1.2 Hz, 1H), 8.15 (d, J = 2.3 Hz, 1H), 8.19 (d, J = 2.2 Hz, 1H).

^{13}C NMR (126 MHz, $CDCl_3$) δ: 14.19, 22.66, 23.99, 31.75, 36.18, 89.73, 105.27, 115.68, 116.54, 126.94, 128.43, 142.39, 144.88, 163.03.

GC-MS (*m/z*): 281.1 $[M]^+$. **m.p.**<80 ºC

Structure of the compound was further confirmed by 1D (DEPT-135) and 2D NMR (COSY, HMBC and HSQC). For these spectra's with important correlation see NMR supporting information.

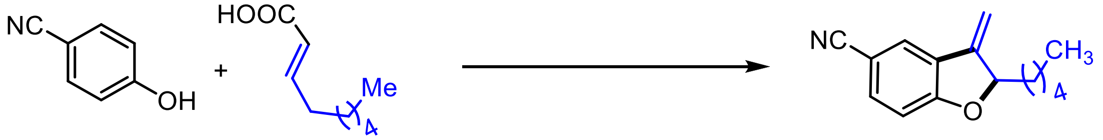

2-ethyl-3-methylene-2,3-dihydrobenzofuran-5-carbonitrile (Table 1; entry 3b) was synthesized by general procedure A. Desired product was obtained as yellow viscous liquid in 69% yield after column chromatography of the crude reaction mixture (silica gel, mesh 100-200; petroleum ether: ethyl acetate 98:2)

^{1}H NMR (400 MHz, CDCl$_3$) δ: 0.85 – 0.93 (m, 3H), 1.26 – 1.39 (m, 5H), 1.40 – 1.56 (m, 1H), 1.67 – 1.88 (m, 2H), 5.03 (dd, *J* = 2.7, 0.9 Hz, 1H), 5.25 (ddt, *J* = 6.9, 3.9, 2.9 Hz, 1H), 5.51 (dd, *J* = 3.0, 0.9 Hz, 1H), 6.88 (d, *J* = 8.4 Hz, 1H), 7.48 (dd, *J* = 8.4, 1.8 Hz, 1H), 7.65 (d, *J* = 1.7 Hz, 1H).

^{13}C NMR (101 MHz, CDCl$_3$) δ: 14.28, 22.78, 24.30, 31.87, 36.38, 87.80, 103.24, 104.13, 109.67, 111.76, 125.58, 127.66, 135.23, 145.45, 165.62.

ESI-MS calculated for $C_{15}H_{17}KNO[M-K]^+$, 266.0942 found 266.0943.

GC-MS (*m/z*): 227.2 $[M]^+$.

Structure of the compound was further confirmed by DEPT-135.

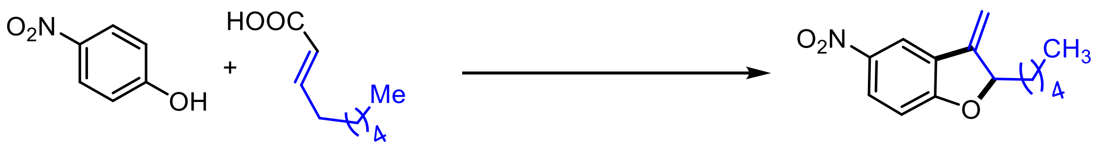

2-ethyl-3-methylene-5-nitro-2,3-dihydrobenzofuran (Table 1; entry 3c) was synthesized by general procedure A. Desired product was obtained as brown viscous liquidin 47% yield after column chromatography of the crude reaction mixture (silica gel, mesh 100-200; petroleum ether)

^{1}H NMR (400 MHz, CDCl$_3$)*δ*:0.80 – 0.85 (m, 3H), 1.23 – 1.5 (m, 6H), 1.68 – 1.79(m, 2H), 5.0 (d,*J* = 4Hz, 1H), 5.27 (dd, *J* =8, 4 Hz, 1H), 5.53 (d, *J* = 4 Hz, 1H), 6.8 (d, *J* =8Hz, 1H), 8.08 (dd,*J* =8, 4 Hz, 1H), 8.19 (d,*J* =4Hz, 1H).

^{13}C NMR (101 MHz, CDCl$_3$) δ: 14.20, 22.70, 24.18, 31.77, 36.30, 88.76, 103.83, 110.61, 117.60, 127.39, 142.20, 145.14, 167.20.

HRMS (ESI) calculated for $C_{14}H_{17}NNaO_3[M-Na]^+$, 270.1101found 270.1108.

GC-MS (*m/z*): 247.1 $[M]^+$.

1D (DEPT-135) and 2D NMR (COSY, NOESY, HSQC and HMBC)[2]

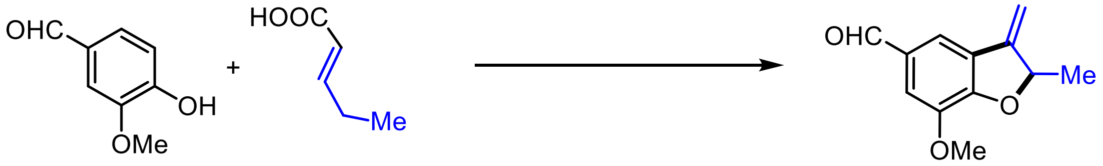

7-methoxy-2-methyl-3-methylene-2,3-dihydrobenzofuran-5-carbaldehyde (Table 1; entry 3d) was synthesized by general procedure A. Desired product was obtained as white crystalline solid in 59% yield after column chromatography of the crude reaction mixture (silica gel, mesh 100-200; petroleum ether: ethyl acetate 98:2)

^{1}H NMR (400 MHz, $CDCl_3$) δ: 1.58 (d, J = 6.5 Hz, 3H), 3.94(s, 3H), 5.0 (dd, J = 2.6, 0.9 Hz, 1H), 5.44 (dtd, J = 9.4, 6.5, 2.8 Hz, 1H),5.50 (dd, J = 3.1, 0.9 Hz, 1H), 7.35 (d, J = 1.4 Hz, 1H), 7.53 (d, J = 1.5 Hz, 1H), 9.84 (s, 1H).

^{13}C NMR (101 MHz, $CDCl_3$) δ: 21.72, 56.31, 85.11, 102.14, 111.78, 118.40, 127.27, 131.33, 145.95, 147.54, 156.31, 190.82.

GC-MS (*m/z*): 204.1 $[M]^+$.

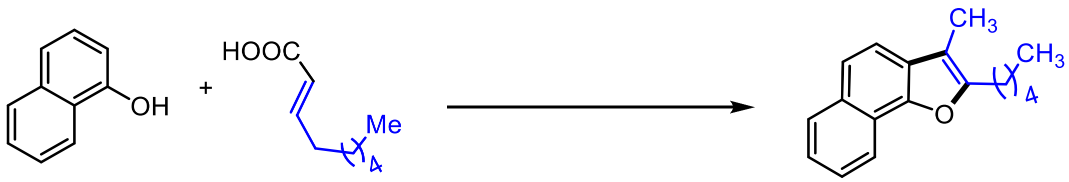

2-ethyl-3-methylnaphtho[1,2-b]furan (Table 1; entry 4e) was synthesized by general procedure A. Desired product was obtained as brown viscous liquid in 45% yield after column chromatography of the crude reaction mixture (silica gel, mesh 100-200; petroleum ether).

^{1}H NMR (500 MHz, $CDCl_3$) δ: 0.88 – 0.95 (m, 3H), 1.36 – 1.41 (m, 4H),1.73 – 1.84 (m, 2H), 2.24(s, 3H), 2.81 (t, J = 7.5 Hz, 2H), 7.42 (ddd, J = 8.2, 6.8, 1.3 Hz, 1H), 7.51 – 7.59 (m, 2H), 7.63 (d, J = 8.5 Hz, 1H), 7.91 (d, J = 8.2 Hz, 1H), 8.27 (dd, J = 8.3, 1.1 Hz, 1H).

^{13}C NMR (126 MHz, $CDCl_3$) δ: 8.30, 14.25, 22.68, 26.62, 28.51, 31.63, 110.78, 118.15, 120.04, 121.36, 122.61, 124.47, 125.82, 126.13, 128.53, 131.09, 149.12, 154.19.

GC-MS (*m/z*): 252.2 $[M]^+$.

Structure of the compound was further confirmed by 1D (DEPT-135)

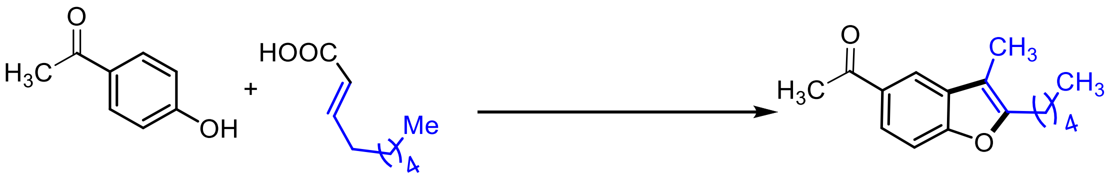

1-(3-methyl-2-pentylbenzofuran-5-yl)ethanone(Table 1; entry 4f) was synthesized by general procedure A. Desired product was obtained as yellow viscous liquidin 59% yield after column chromatography of the crude reaction mixture (silica gel, mesh 100-200; petroleum ether)

^{1}H NMR (400 MHz, $CDCl_3$) δ: 0.87 – 0.92 (m, 3H), 1.30 – 1.37 (m, 4H), 1.65 – 1.76 (m, 2H), 2.19(s, 3H), 2.66(s, 3H), 2.73 (t, *J* = 7.5 Hz, 2H), 7.39 (dd, *J* = 8.6, 0.6 Hz, 1H), 7.87 (dd, *J* = 8.6, 1.9 Hz, 1H), 8.06 (dd, *J* = 1.9, 0.6 Hz, 1H).

^{13}C NMR (126 MHz, $CDCl_3$) δ: 8.07, 14.20, 22.61, 26.47, 27.01, 27.97, 31.56, 110.43, 110.61, 120.04, 124.35, 130.89, 132.21, 156.53, 156.88, 198.17

HRMS (ESI)calculated for $C_{16}H_{20}NaO_2[M\text{-}Na]^+$, 267.1356 found 267.1357

GC-MS (*m/z*): 244.2 $[M]^+$.

Structure of the compound was further confirmed by 1D (DEPT-135)

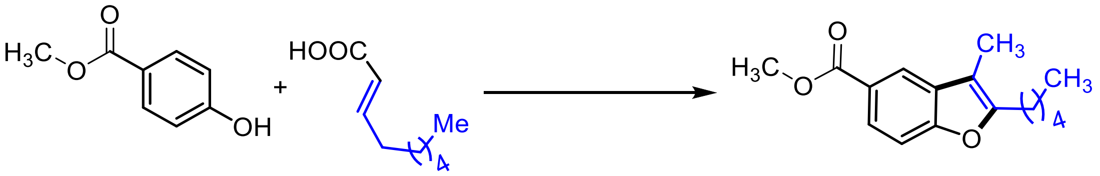

Methyl 2-ethyl-3-methylbenzofuran-5-carboxylate (Table 1; entry 4g) was synthesized by general procedure A. Desired product was obtained as yellow viscous liquidin 51% yield after column chromatography of the crude reaction mixture (silica gel, mesh 100-200; petroleum ether: ethyl acetate 98:2)

^{1}H NMR (400 MHz, CDCl$_3$) δ: 0.87 – 0.92 (m, 3H), 1.29 – 1.38 (m, 4H), 1.66 – 1.75 (m, 2H), 2.19 (d, *J* = 0.6 Hz, 3H), 2.72 (t, *J* = 7.5 Hz, 2H),3.93 (s, 3H), 7.38 (dd, *J* = 8.5, 0.6 Hz, 1H), 7.93 (dd, *J* = 8.6, 1.8 Hz, 1H), 8.15 (dd, *J* = 1.8, 0.6 Hz, 1H).

^{13}C NMR (101 MHz, CDCl$_3$) δ: 8.05, 14.20, 22.61, 26.44, 27.99, 31.56, 52.22, 110.26, 110.51, 121.30, 124.41, 125.17, 130.75, 156.28, 156.78, 167.82.

HRMS (ESI) calculated for $C_{16}H_{20}NaO_3[M\text{-}Na]^+$, 283.1305 found 283.1304.

GC-MS (*m/z*): 260.1 $[M]^+$.

Structure of the compound was further confirmed by 1D (DEPT-135)

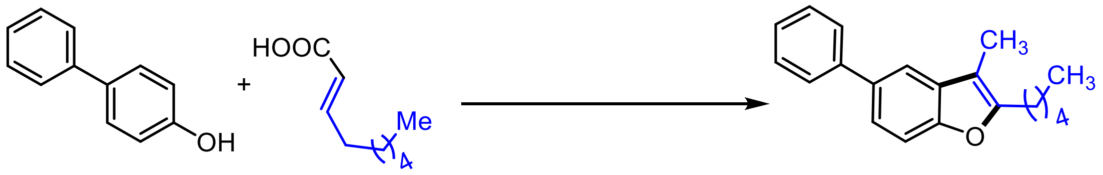

2-ethyl-3-methyl-5-phenylbenzofuran (Table 1; entry 4h) was synthesized by general procedure A. Desired product was obtained as viscous liquidin 48% yield after column chromatography of the crude reaction mixture (silica gel, mesh 100-200; petroleum ether)

^{1}H NMR (400 MHz, CDCl$_3$) δ: 0.88 – 0.94 (m, 3H), 1.35 (dt, *J* = 7.8, 2.6Hz, 4H), 1.68 – 1.79 (m, 2H), 2.20(s, 3H), 2.74 (t, *J* = 7.4 Hz, 2H), 7.31 – 7.37 (m, 1H), 7.42 – 7.48 (m, 4H), 7.58 – 7.67 (m, 3H).

^{13}C NMR (101 MHz, CDCl$_3$) δ: 8.18, 14.26, 22.67, 26.50, 28.15, 31.58, 109.91, 110.81, 117.46, 122.84, 126.87, 127.65, 128.88, 131.18, 135.88, 142.27, 153.65, 155.52

GC-MS (*m/z*): 278.1 $[M]^+$.

Structure of the compound was further confirmed by DEPT-135.

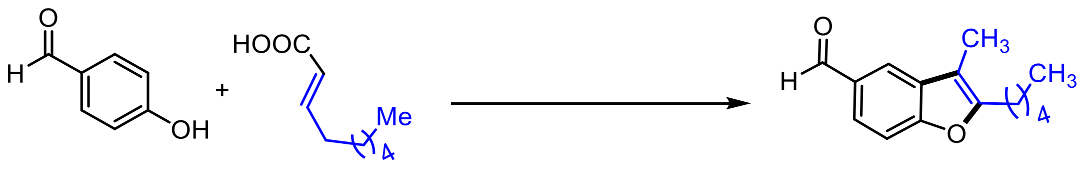

2-ethyl-3-methylbenzofuran-5-carbaldehyde (Table 1; entry 4i) was synthesized by general procedure A. Desired product was obtained as yellow viscous liquidin 60% yield after column chromatography of the crude reaction mixture (silica gel, mesh 100-200; petroleum ether: ethyl acetate 98:2)

^{1}H NMR (400 MHz, CDCl$_3$) δ: 0.87 – 0.92 (m, 3H), 1.30 – 1.38 (m, 4H), 1.68 – 1.77 (m, 2H), 2.20(s, 3H), 2.73 (t, *J* = 7.5 Hz, 2H),7.46 (d, *J* = 8.4 Hz, 1H), 7.76 (dd, *J* = 8.4, 1.7 Hz, 1H), 7.98 (dd, *J* = 1.7, 0.6 Hz, 1H), 10.05 (s, 1H).

^{13}C NMR (126 MHz, CDCl$_3$) δ: 8.04, 14.19, 22.61, 26.48, 27.94, 31.56, 110.46, 111.36, 121.64, 125.63, 131.43, 131.77, 156.99, 157.75, 192.30.

HRMS (ESI)calculated for $C_{15}H_{18}NaO_2$[M-Na]$^+$, 253.1199found 253.1204.

GC-MS (*m/z*): 230 [M]$^+$.

Structure of the compound was further confirmed by 1D (DEPT-135)

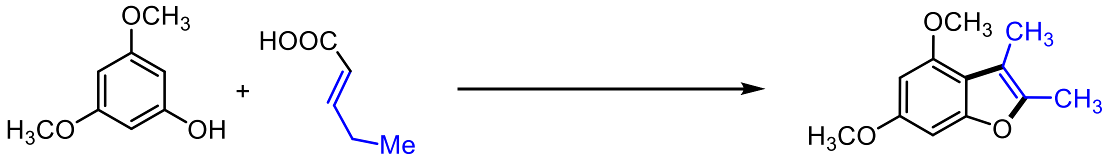

4,6-dimethoxy-2,3-dimethylbenzofuran (Table 1; entry 4j) was synthesized by general procedure A. Desired product was obtained as white crystalline solidin 57% yield after column chromatography of the crude reaction mixture (silica gel, mesh 100-200; petroleum ether)

^{1}H NMR (500 MHz, CDCl$_3$) δ: 2.23 (d, *J* = 1.1 Hz, 3H), 2.30 (d, *J* = 1.1 Hz, 3H), 3.81 (s, 3H), 3.85(s, 3H), 6.25 (d, *J* = 2.0 Hz, 1H), 6.54 (d, *J* = 1.9 Hz, 1H).

^{13}C NMR (101 MHz, CDCl$_3$) δ: 9.96, 11.59, 55.52, 55.87, 88.11, 93.69, 109.64, 113.38, 147.75, 154.51, 155.69, 158.16

HRMS (ESI)calculated for $C_{12}H_{15}O_3$[M-H]$^+$, 207.1016 found 207.1013.

GC-MS (*m/z*): 206.1 [M]$^+$. **m.p.**<80 ºC

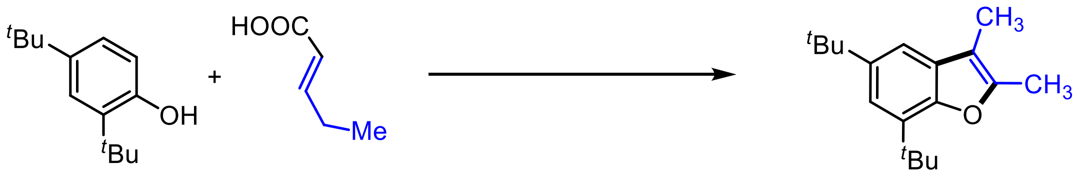

5,7-di-tert-butyl-2,3-dimethylbenzofuranfuran(Table 1; entry 4k) was synthesized by general procedure A. Desired product was obtained as light brown crystalline solidin 50% yield after column chromatography of the crude reaction mixture (silica gel, mesh 100-200; petroleum ether)

^{1}H NMR (500 MHz, CDCl$_3$) δ: 1.40 (s, 9H), 1.52 (s, 9H), 2.16 (t, J = 1.0 Hz, 3H), 2.39 (d, J = 1.0 Hz, 3H), 7.19 (d, J = 2.0 Hz, 1H), 7.24 – 7.28 (m, 1H).

^{13}C NMR (101 MHz, CDCl$_3$) δ: 8.22, 12.14, 30.18, 32.22, 34.62, 35.06, 109.57, 112.73, 117.84, 130.53, 133.25, 144.90, 149.81, 150.32.

GC-MS (*m/z*): 258.1 [M]$^+$.

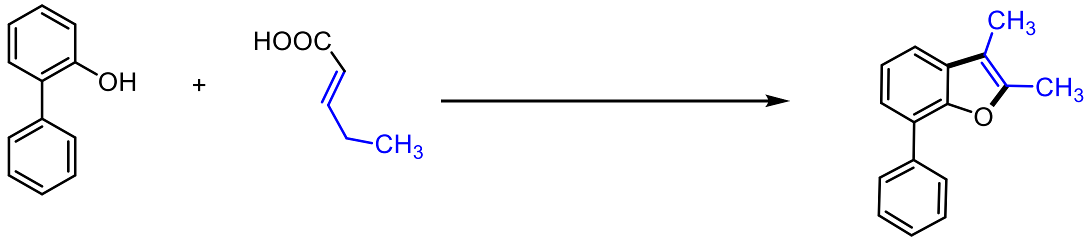

2,3-dimethyl-7-phenylbenzofuran(Scheme 3, R$_1$= R$_2$= CH$_3$) was synthesized by general procedure A. Desired product was obtained as yellow solidin 42% yield after column chromatography of the crude reaction mixture (silica gel, mesh 100-200; petroleum ether)

^{1}H NMR (500 MHz, CDCl$_3$) δ:2.22 (q, *J* = 0.9 Hz, 3H),2.44 (d, *J* = 1.0 Hz, 3H), 7.26 – 7.35 (m, 1H), 7.37 – 7.43 (m, 3H), 7.50 – 7.54 (m, 2H), 7.87 – 7.91 (m, 2H).

^{13}C NMR (101 MHz, CDCl$_3$) δ: 8.24, 12.16, 110.03, 117.93, 122.72, 122.91, 124.72, 127.63, 128.07, 128.73, 128.86, 131.45, 137.08, 151.02

GC-MS (*m/z*): 222.1[M]$^+$.

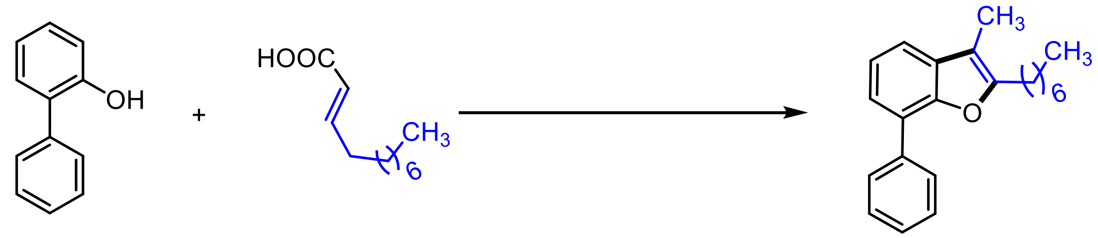

2-ethyl-3-methyl-7-phenylbenzofuran (Scheme 3, R_1=$CH_3$and R_2= $[CH_2]_6CH_3$) was synthesized by general procedure A. Desired product was obtained as brown liquidin 33% yield after column chromatography of the crude reaction mixture (silica gel, mesh 100-200; petroleum ether)

^{1}H NMR (500 MHz, CDCl$_3$) δ:0.89 (t, *J* = 6.9 Hz, 3H), 1.23 – 1.42 (m, 8H), 1.72 (p, *J* = 7.5 Hz, 2H), 2.19(s, 3H), 2.75 (t, *J* = 7.4 Hz, 2H), 7.28 (t, *J* = 7.6 Hz, 1H), 7.38 (qd, *J* = 7.2, 1.3 Hz, 3H), 7.47 – 7.51 (m, 2H), 7.86 – 7.90 (m, 2H).

^{13}C NMR (101 MHz, CDCl$_3$) δ: 8.22, 14.32, 22.87, 26.43, 28.52, 29.26, 29.40, 32.05, 109.72, 118.02, 122.66, 122.84, 124.71, 127.57, 128.71, 128.82, 131.49, 137.13, 151.17, 155.04.

GC-MS (*m/z*): 306 [M]$^+$.

Structure of the compound was further confirmed by 1D NMR (DEPT-135) and 2D NMR (COSY, NOESY, HSQC and HMBC). For these spectra's with important correlation see NMR supporting information.

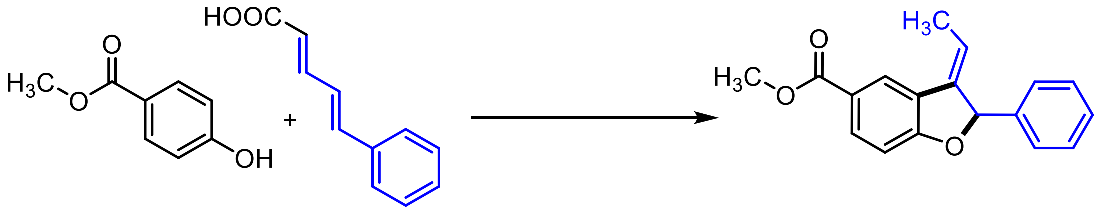

(E)-methyl 3-ethylidene-2-phenyl-2,3-dihydrobenzofuran-5-carboxylate (Table 2; entry 6a) was synthesized by general procedure A. Desired product was obtained as black viscous liquid in 66%[b]yield after column chromatography of the crude reaction mixture (silica gel, mesh 100-200; petroleum ether: ethyl acetate 97:3)

^{1}H NMR (500 MHz, CDCl$_3$) δ: 2.13 (t, *J* = 1.7 Hz, 3H), 3.90 (s, 3H), 5.65 (dt, *J* = 3.4, 1.6 Hz, 1H), 5.94 (dt, *J* = 3.7, 1.9 Hz, 1H), 6.83 (dd, *J* = 8.4, 0.8 Hz, 1H), 7.33 – 7.43 (m, 5H), 7.85 (ddd, *J* = 8.5, 2.2, 0.8 Hz, 1H), 7.90 (d, *J* = 2.1 Hz, 1H).

^{13}C NMR (126 MHz, CDCl$_3$) δ: 18.22, 52.04, 77.87, 116.02, 122.05, 122.68, 122.91, 125.49, 127.10, 128.64, 128.84, 129.07, 131.46, 140.85, 157.48, 167.02.

GC-MS (*m/z*): 280.1 $[M]^+$.

Structure of the compound was further confirmed by 1D NMR (DEPT-135, NOE) and 2D NMR (COSY, NOESY). For these spectra's with important correlation see NMR supporting information.

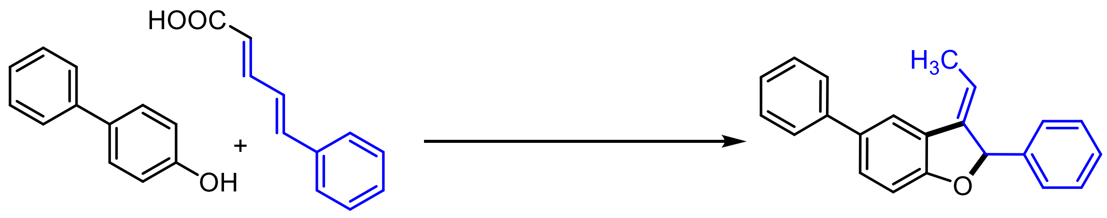

(E)-3-ethylidene-2,5-diphenyl-2,3-dihydrobenzofuran (Table 2; entry 6b)was synthesized by general procedure A. Desired product was obtained as black viscous liquid in 51%[b] yield after column chromatography of the crude reaction mixture (silica gel, mesh 100-200; petroleum ether: ethyl acetate 98:2)

^{1}H NMR (400 MHz, CDCl$_3$) δ: 2.18 (t, *J* = 1.6 Hz, 3H), 5.70 (dt, *J* = 2.9, 1.5 Hz, 1H), 5.93 (dd, *J* = 3.6, 1.8 Hz, 1H), 6.94 (dd, *J* = 8.2, 1.3 Hz, 1H), 7.32 – 7.52 (m, 10H), 7.57 – 7.63 (m, 2H).

^{13}C NMR (101 MHz, CDCl$_3$) δ: 18.32, 77.39 116.47, 122.26, 122.47, 123.51, 126.93, 127.18, 128.17, 128.46, 128.81, 128.90, 129.73, 134.33, 141.24, 141.38, 153.06.

GC-MS (*m/z*): 298.1 $[M]^+$.

Structure of the compound was further confirmed by 1D (DEPT-135)

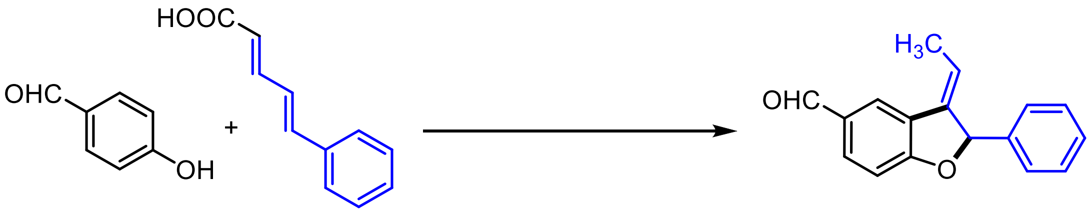

(E)-3-ethylidene-2-phenyl-2,3-dihydrobenzofuran-5-carbaldehyde (Table 2; entry 6c) was synthesized by general procedure A. Desired product was obtained as yellow viscous liquid in 61% yield after column chromatography of the crude reaction mixture (silica gel, mesh 100-200; petroleum ether: ethyl acetate 98:2)

^{1}H NMR (500 MHz, $CDCl_3$)δ: 2.15 (q, *J* = 2.0 Hz, 3H),5.69 (dt, *J* = 3.4, 1.7 Hz, 1H), 5.98 (dq, *J* = 3.6, 1.8 Hz, 1H), 6.90 (d, *J* = 8.2 Hz, 1H), 7.32 – 7.43 (m, 5H), 7.66 (dd, *J* = 8.3, 2.0 Hz, 1H), 7.74 (d, *J* = 2.1 Hz, 1H), 9.86 (s, 1H).

^{13}C NMR (126 MHz, $CDCl_3$) δ: 18.23, 78.21, 116.63, 122.50, 123.25, 124.92, 127.15, 128.73, 128.82, 128.94, 130.2, 132.75, 140.60, 158.94, 191.06.

HRMS (ESI) calculated for $C_{17}H_{14}NaO_2$[M-Na]$^+$, 273.0886, found 273.0887.

GC-MS (*m/z*): 250.1 $[M]^+$.

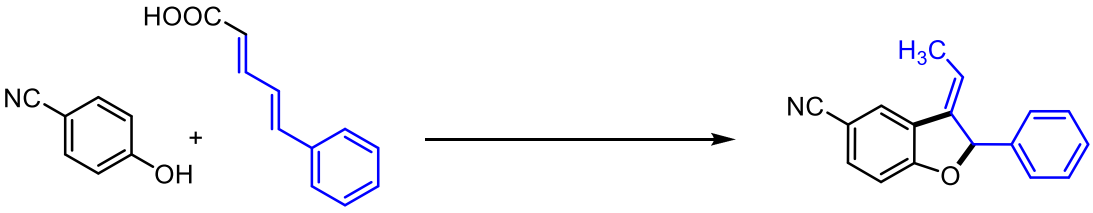

(E)-3-ethylidene-2-phenyl-2,3-dihydrobenzofuran-5-carbonitrile(Table 2; entry 6d) was synthesized by general procedure A. Desired product was obtained as greenish black viscous liquid in 48% yield after column chromatography of the crude reaction mixture (silica gel, mesh 100-200; petroleum ether: ethyl acetate 99:1)

^{1}H NMR (500 MHz, $CDCl_3$)δ: 2.09 (t, *J* = 1.7 Hz, 3H), 5.70 (dq, *J* = 3.2, 1.5 Hz, 1H), 5.96 (dq, *J* = 3.4, 1.8 Hz, 1H), 6.83 (d, *J* = 8.3 Hz, 1H), 7.32 – 7.42 (m, 6H), 7.45 (d, *J* = 2.0 Hz, 1H).

^{13}C NMR (126 MHz, $CDCl_3$)δ:18.01, 78.07, 104.28, 117.05, 119.50, 123.13, 123.76, 127.13, 127.76, 128.05, 128.91, 128.96, 133.68, 140.33, 157.02.

HRMS (ESI) calculated for $C_{17}H_{13}NNaO[M-H]^{+}$, 270.0889, found 270.0990.

GC-MS (*m/z*): 247.1 $[M]^{+}$.

Structure of the compound was further confirmed by 1D (DEPT-135)

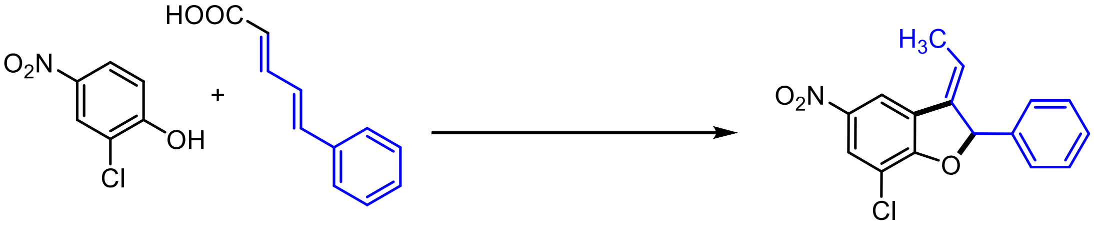

(E)-7-chloro-3-ethylidene-5-nitro-2-phenyl-2,3-dihydrobenzofuran (Table 2; entry 6e) was synthesized by general procedure A. Desired product was obtained as deep yellow solid in 55%[b] yield after column chromatography of the crude reaction mixture (silica gel, mesh 100-200; petroleum ether: ethyl acetate 98:2)

^{1}H NMR (400 MHz, $CDCl_3$) δ: 2.16 (t, *J* = 1.6 Hz, 3H), 5.86 (dq, *J* = 3.1, 1.5 Hz, 1H), 6.13 (dq, *J* = 3.5, 1.7 Hz, 1H), 7.33 – 7.43 (m, 5H), 7.98 (d, *J* = 2.6 Hz, 1H), 8.15 (d, *J* = 2.7 Hz, 1H).

^{13}C NMR (101 MHz, $CDCl_3$) δ: 18.34, 78.73, 117.80, 121.90, 123.76, 124.14, 125.87, 126.91, 128.10, 129.08, 129.12, 139.69, 142.46, 154.70.

HRMS (ESI) calculated for $C_{16}H_{12}ClNNaO_3$ $[M-Na]^{+}$, 324.0398, found 324.0397.

GC-MS (*m/z*): 301.2 $[M]^{+}$.**m.p.**112-114 ºC

Structure of the compound was further confirmed by 1D (DEPT-135)

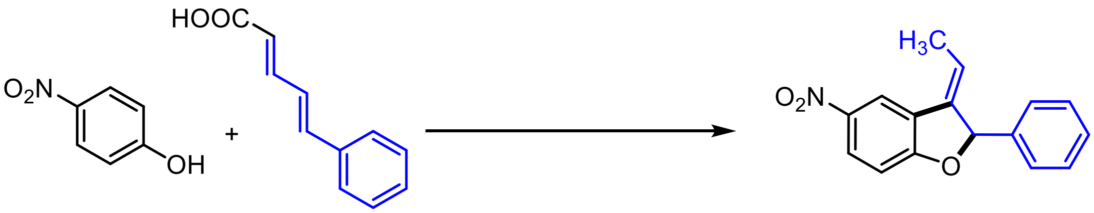

(E)-3-ethylidene-5-nitro-2-phenyl-2,3-dihydrobenzofuran (Table 2; entry 6f) was synthesized by general procedure A. Desired product was obtained as deep brown viscous liquid in 57% yield after column chromatography of the crude reaction mixture (silica gel, mesh 100-200; petroleum ether: ethyl acetate 98:2)

^{1}H NMR (500 MHz, $CDCl_3$) δ: 2.15 (t, J = 1.7 Hz, 3H), 5.75 (dt, J = 3.4, 1.7 Hz, 1H), 6.01 (dq, J = 3.6, 1.8 Hz, 1H), 6.84 (d, J = 8.8 Hz, 1H), 7.34 – 7.44 (m, 5H), 8.04 (dd, J = 8.9, 2.7 Hz, 1H), 8.08 (d, J = 2.7 Hz, 1H).

^{13}C NMR (126 MHz, $CDCl_3$) δ:18.18, 78.53, 116.44, 119.67, 122.97, 123.21, 125.66, 126.59, 127.18, 128.21, 129.02, 129.04, 140.14, 158.89.

GC-MS (*m/z*): 267.1 $[M]^+$.

Structure of the compound was further confirmed by 1D (DEPT-135)

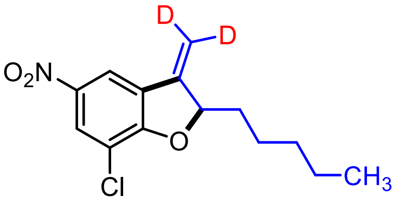

(Scheme 4; compound 3'a)**:** Following compound was synthesized by general procedure B. Desired product was obtained as yellow crystalline solid in 65% (deuterium content 95%) yield after column chromatography of the crude reaction mixture (silica gel, mesh 100-200; petroleum ether)

^{1}H NMR (500 MHz, $CDCl_3$) δ: 0.88 – 0.92 (m, 3H), 1.29 – 1.56 (m, 6H), 1.76 – 1.84 (m, 1H), 1.91 (dddd, J = 14.6, 10.2, 5.8, 4.1 Hz, 1H), **5.12 (1D)**, 5.45 (dd, J = 7.6, 4.1 Hz, 1H), **5.63 (1D)**, 8.16 (d, J = 2.3 Hz, 1H), 8.19 (d, J = 2.2 Hz, 1H).

HRMS (ESI) calculated for $C_{14}H_{14}ClD_2NNaO_3$ [M-Na]$^+$, 306.0836, found 306.0836.

GC-MS (*m/z*): 283.2 [M]$^+$.

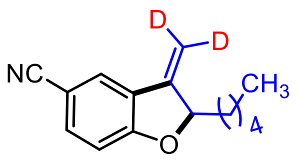

***(Scheme 4; compound 3'b)*:** Following compound was synthesized by general procedure B. Desired product was obtained as yellow liquid in 47% (deuterium content 93%) yield after column chromatography of the crude reaction mixture (silica gel, mesh 100-200; petroleum ether)

^{1}H NMR (400 MHz, CDCl$_3$) δ:7.64 (d, J = 1.7 Hz, 1H), 7.48 (dd, J = 8.4, 1.8 Hz, 1H), 6.87 (d, J = 8.4 Hz, 1H), **5.49 (1D)**, 5.26 (dd, J = 7.8, 3.9 Hz, 1H), **5.01 (1D)**, 1.88 – 1.79 (m, 1H), 1.73 (dddd, J = 19.2, 10.1, 8.3, 4.4 Hz, 1H), 1.58 – 1.26 (m, 6H), 0.94 – 0.84 (m, 3H).

^{13}C NMR (101 MHz, CDCl$_3$) δ: 165.51, 145.18, 135.11, 127.56, 125.49, 119.51, 111.65, 104.02, 87.66, 36.29, 31.78, 24.21, 22.69, 14.19.

GC-MS (*m/z*): 229.1 [M]$^+$.

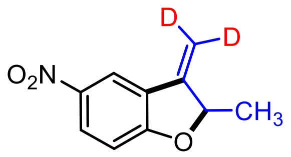

***(Scheme 4; compound 3'c)*:** Following compound was synthesized by general procedure B. Desired product was obtained as yellow solid in 43% (deuterium content 92%) yield after column chromatography of the crude reaction mixture (silica gel, mesh 100-200; petroleum ether)

^{1}H NMR (500 MHz, CDCl$_3$) δ: 8.25 (dt, J = 2.6, 1.3 Hz, 1H), 8.15 (ddd, J = 8.9, 2.4, 1.2 Hz, 1H), 6.86 (dd, J = 8.8, 1.2 Hz, 1H), **5.57 (1D)**, 5.43 (q, J = 6.6 Hz, 1H), **5.07 (1D)**, 1.55 (dd, J = 6.5, 0.9 Hz, 3H).

^{13}C NMR (126 MHz, CDCl$_3$) δ: 166.82, 146.22, 142.25, 127.40, 126.94, 117.71, 110.68, 84.99, 21.70.

GC-MS (*m/z*): 193.1 $[M]^+$.

Structure of the compound was further confirmed by 1D (DEPT-135)

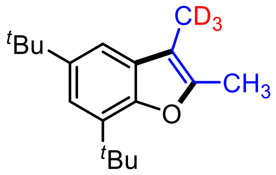

***(Scheme 4; compound 4'a)*:** Following compound was synthesized by general procedure B. Desired product was obtained as light white solid in 53% (deuterium content 93%) yield after column chromatography of the crude reaction mixture (silica gel, mesh 100-200; petroleum ether)

^{1}H NMR (400 MHz, $CDCl_3$) δ: 1.38 (s, 9H), 1.49 (s, 9H),**2.11 (3D)**, 2.38 (s, 3H), 7.17 (d, *J* = 2.0 Hz, 1H), 7.23 (d, *J* = 2.0 Hz, 1H).

GC-MS (*m/z*): 261.2 $[M]^+$.

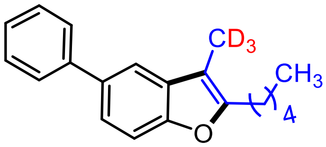

***(Scheme 4; compound 4'b)*:** Following compound was synthesized by general procedure B. Desired product was obtained as yellow solid in 42% (deuterium content 94%) yield after column chromatography of the crude reaction mixture (silica gel, mesh 100-200; petroleum ether)

^{1}H NMR (400 MHz, $CDCl_3$) δ: 7.65 (d, *J* = 1.4 Hz, 1H), 7.63 (dd, *J* = 2.0, 0.9 Hz, 1H), 7.60 (t, *J* = 1.3 Hz, 1H), 7.48 – 7.44 (m, 2H), 7.43 (t, *J* = 1.4 Hz, 2H), 7.36 – 7.31 (m, 1H), 2.74 (t, *J* = 7.4 Hz, 2H), **2.17 (3D)**, 1.78 – 1.69 (m, 2H), 1.36 (dq, *J* = 7.2, 3.8, 3.1 Hz, 4H), 0.93 – 0.89 (m, 3H).

^{13}C NMR (126 MHz, $CDCl_3$) δ: 155.57, 153.71, 142.32, 135.92, 131.22, 128.88, 127.66, 126.87, 122.85, 117.47, 110.81, 31.60, 28.15, 26.53, 22.67, 14.23.

GC-MS (*m/z*): 281.2 $[M]^+$.

Structure of the compound was further confirmed by 1D (DEPT-135)

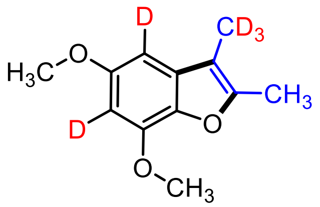

***(Scheme 4; compound 4'c)*: ** Following compound was synthesized by general procedure B. Desired product was obtained as yellow solid in 50% (-CD_3, deuterium content>97%) yield after column chromatography of the crude reaction mixture (silica gel, mesh 100-200; petroleum ether)

^{1}H NMR (400 MHz, $CDCl_3$)δ:**6.53 (1D)**, **6.25 (1D)**, 3.84 (s, 3H), 3.81 (s, 3H), 2.29 (s, 3H), **2.24 (3D)**.

GC-MS (*m/z*): 211.1 $[M]^+$.

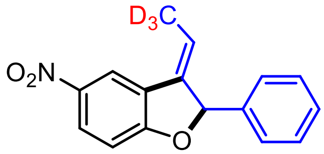

***(Scheme 4; compound 6'a)*: ** Following compound was synthesized by general procedure B. Desired product was obtained as yellow solid in 49% (deuterium content 91%) yield after column chromatography of the crude reaction mixture (silica gel, mesh 100-200; petroleum ether)

^{1}H NMR (400 MHz, $CDCl_3$) δ: 8.08 (d, J = 2.7 Hz, 1H), 8.05 (dd, J = 8.8, 2.7 Hz, 1H), 7.42 – 7.35 (m, 5H), 6.84 (d, J = 8.8 Hz, 1H), 6.03 – 5.98 (m, 1H), 5.75 (d, J = 3.5 Hz, 1H), **2.13 (3D)**.

^{13}C NMR (101 MHz, $CDCl_3$) δ: 158.93, 141.95, 140.19, 129.06, 127.21, 125.70, 123.26, 123.03, 119.71, 116.48, 78.57.

GC-MS (*m/z*): 270.1 $[M]^+$.

Structure of the compound was further confirmed by 1D (DEPT-135)

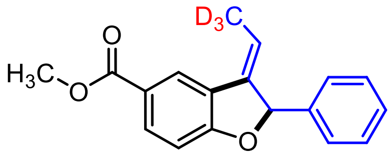

***(Scheme 4; compound 6'b)*:**Following compound was synthesized by general procedure B. Desired product was obtained black viscous liquid in 59% (deuterium content 92%) yield[b] after column chromatography of the crude reaction mixture (silica gel, mesh 100-200; petroleum ether: ethyl acetate 97:3)

^{1}H NMR (400 MHz, CDCl$_3$) δ: **2.10 (3D)**, 3.89 (s, 3H), 5.65 (d, J = 3.4 Hz, 1H), 5.93 (d, J = 3.4 Hz, 1H), 6.81 (d, J = 8.4 Hz, 1H), 7.31 – 7.44 (m, 5H), 7.83 (dd, J = 8.4, 2.1 Hz, 1H), 7.87 (d, J = 2.1 Hz, 1H).

HRMS (ESI) calculated for $C_{18}H_{13}D_3NaO_3$ $[M\text{-}Na]^+$, 306.1180, found 306.1180.

GC-MS (*m/z*): 283.1 $[M]^+$.

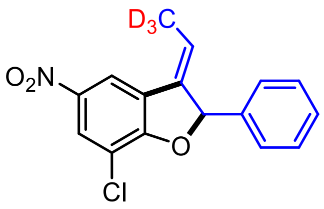

***(Scheme 4; compound 6'c)*:**Following compound was synthesized by general procedure B Desired product was obtained black viscous liquid in 51% (deuterium content 90%) yield after column chromatography of the crude reaction mixture (silica gel, mesh 100-200; petroleum ether: ethyl acetate 97:3)

^{1}H NMR (500 MHz, CDCl$_3$) δ: 8.15 (d, J = 2.7 Hz, 1H), 7.97 (d, J = 2.6 Hz, 1H), 7.41 – 7.34 (m, 5H), 6.13 (d, J = 3.4 Hz, 1H), 5.86 (d, J = 3.6 Hz, 1H), **2.13 (3D)**.

^{13}C NMR (126 MHz, CDCl$_3$) δ: 154.71, 141.34, 139.71, 129.12, 129.09, 128.02, 126.91, 125.88, 124.16, 123.78, 121.91, 117.81, 78.74.

GC-MS (*m/z*): 304.1 $[M]^+$.

Structure of the compound was further confirmed by 1D (DEPT-135)

Mechanistic details:

Exp-1: To an oven-dried screw cap reaction tube charged with a magnetic stir-bar, $Pd(OAc)_2$ (10 mol%, 0.025 mmol, 5.6 mg), 1,10-phenonthroline monohydrate (20 mol%, 0.05 mmol, 10 mg), $Cu(OAc)_2.H_2O$ (0.25 mmol, 50 mg) were added. Then instead of phenol and α,β-unsaturated carboxylic acid , 7-chloro-3-methylene-5-nitro-2-pentyl-2,3-dihydrobenzofuran (table **1**, entry **3a**) was introduced in the reaction mixture. In the reaction tube 4 mL DCE ($ClCH_2CH_2Cl$) was added and O_2 was purged in the reaction mixture for 15 min. Then the reaction mixture was vigorously stirred (900 rpm on Heidolph MR Hei-Standard stirrer) in a preheated oil bath at 130 ºC for 24h. After completion, reaction mixture was filtered through a celite pad with ethylacetate as the washing solvent. Then the mixture was carefully analyzed by GCMS and TLC.

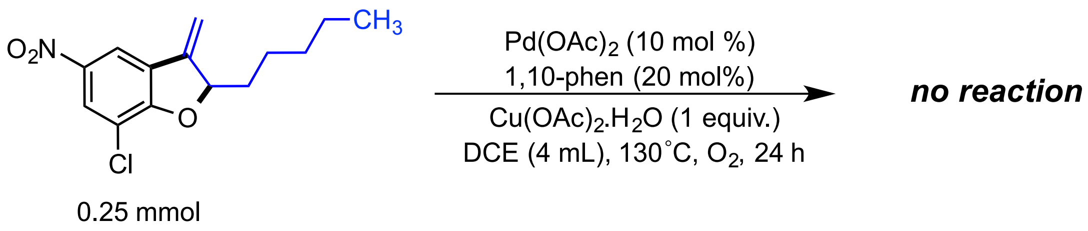

Exp-2[3]**:** 7-chloro-3-methylene-5-nitro-2-pentyl-2,3-dihydrobenzofuran (0.1 mmol, 28 mg) was introduced into an oven dried screw cap reaction tube. In the reaction tube, 10 mL of chloroform was added in the nitrogen atmosphere. Then the reaction mixture was vigorously stirred at 25 ºC and catalytic amount of trifluoro acetic acid was added. After two hours the reaction mixture was carefully analyzed by GCMS and TLC.

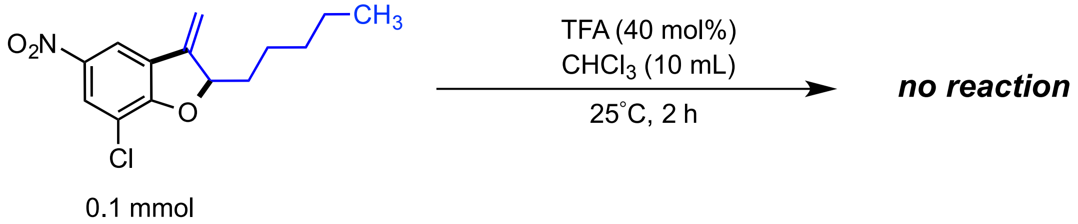

Exp-3: To an oven-dried screw cap reaction tube charged with a magnetic stir-bar, $Pd(OAc)_2$ (10 mol%, 0.025 mmol, 2.8 mg), 1,10-phenonthroline monohydrate (20 mol%, 0.05 mmol, 5 mg), $Cu(OAc)_2.H_2O$ (0.25 mmol, 25 mg) were added. Then [d_5]PhOH (0.375 mmol) and 8-nonenoic acid (0.125 mmol) were introduced in the reaction mixture. In the reaction tube 4 mL DCE ($ClCH_2CH_2Cl$) was added and O_2 was purged in the reaction mixture for 15 min. Then the reaction mixture was vigorously stirred (900 rpm on Heidolph MR Hei-Standard stirrer) in a preheated oil bath at 130 ºC for 24h. After completion, reaction mixture was filtered through a celite pad with ethylacetate as the washing solvent. The ethylacetate layer was washed with brine solution and dried over anhydrous Na_2SO_4, and evaporated under reduced pressure. The residue was purified by column chromatography using silica gel (100-200 mesh size) and petroleum-ether/ ethyl acetate as the eluent.

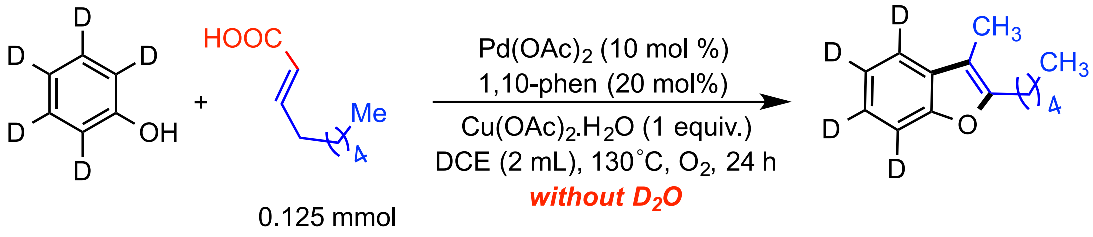

NMR data:

^{1}H NMR (400 MHz, CDCl$_3$) δ: 2.72 (t, J = 7.5 Hz, 2H), 2.16 (s, 3H), 1.75 – 1.66 (m, 2H), 1.33 (tt, J = 7.5, 3.2 Hz, 4H), 0.92 – 0.86 (m, 3H).

References:

[1] S. Agasti, U. Sharma, T. Naveen, D. Maiti, *Chem. Commun.* **2015**, *51*, 5375.

[2] U. Sharma, T. Naveen, A. Maji, S. Manna, D. Maiti, *Angew. Chem. Int. Ed.* **2013**, *52*, 12669.

[3] H. Bhandal, V. F. Patel, G. Pattenden, J. J. Russell, *J. Chem. Soc., Perkin Trans. 1,* **1990**, 2691.

Palladium-Catalyzed Synthesis of 2,3-disubstituted Benzofurans: An Approach Towards the Synthesis of Deuterium Labeled Compounds

Soumitra Agasti,[a] Soham Maity,[a] Kalman J. Szabo,[b,]* and Debabrata Maiti[a,]*

[a]Department of Chemistry, Indian Institute of Technology BombayPowai, Mumbai 400076, India

[b]Department of Organic Chemistry, Stockholm University, SE-106 91 Stockholm, Sweden

Supporting Information (NMR Files)

Table 1, Entry 3a:

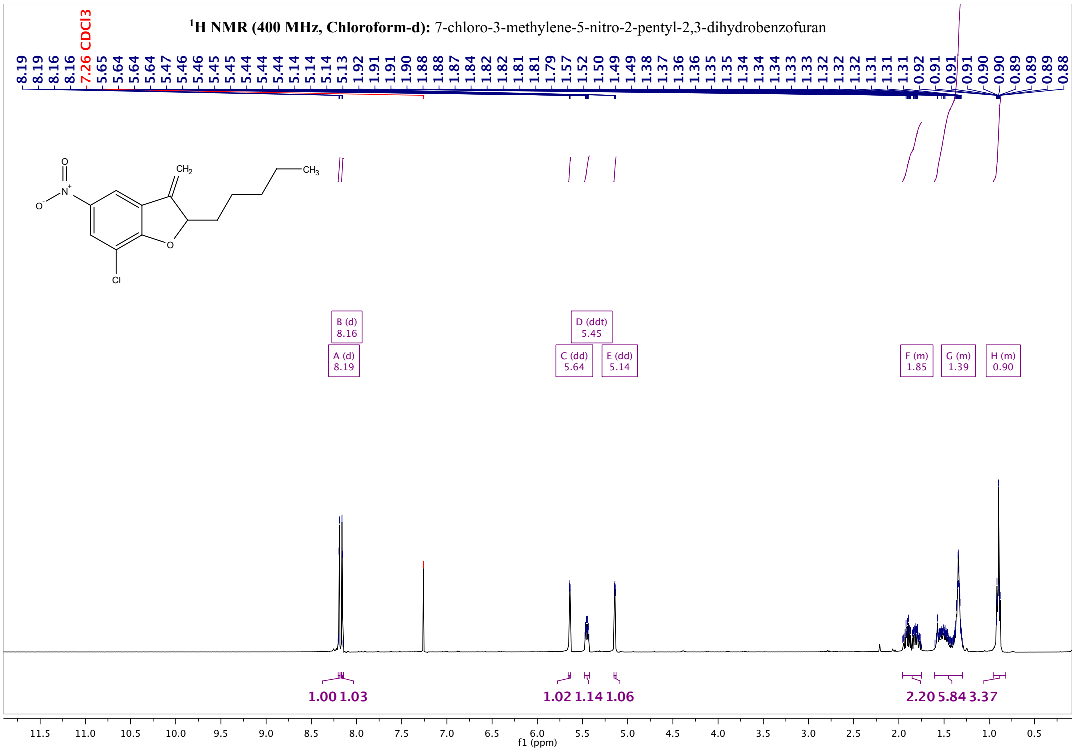

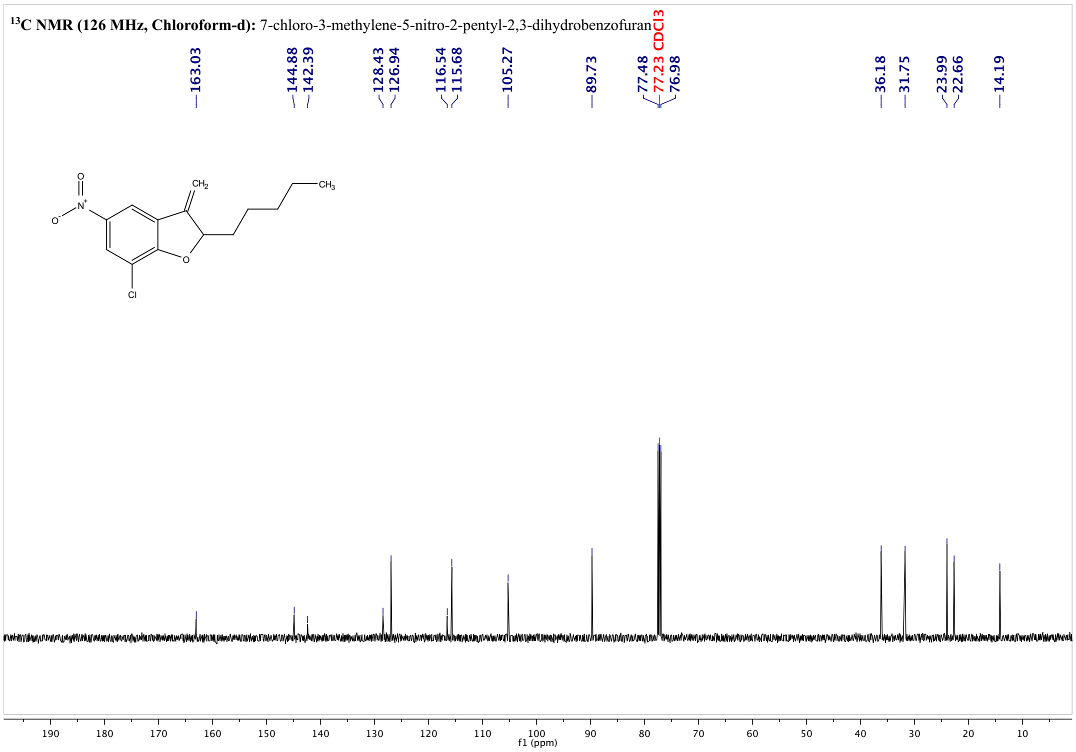

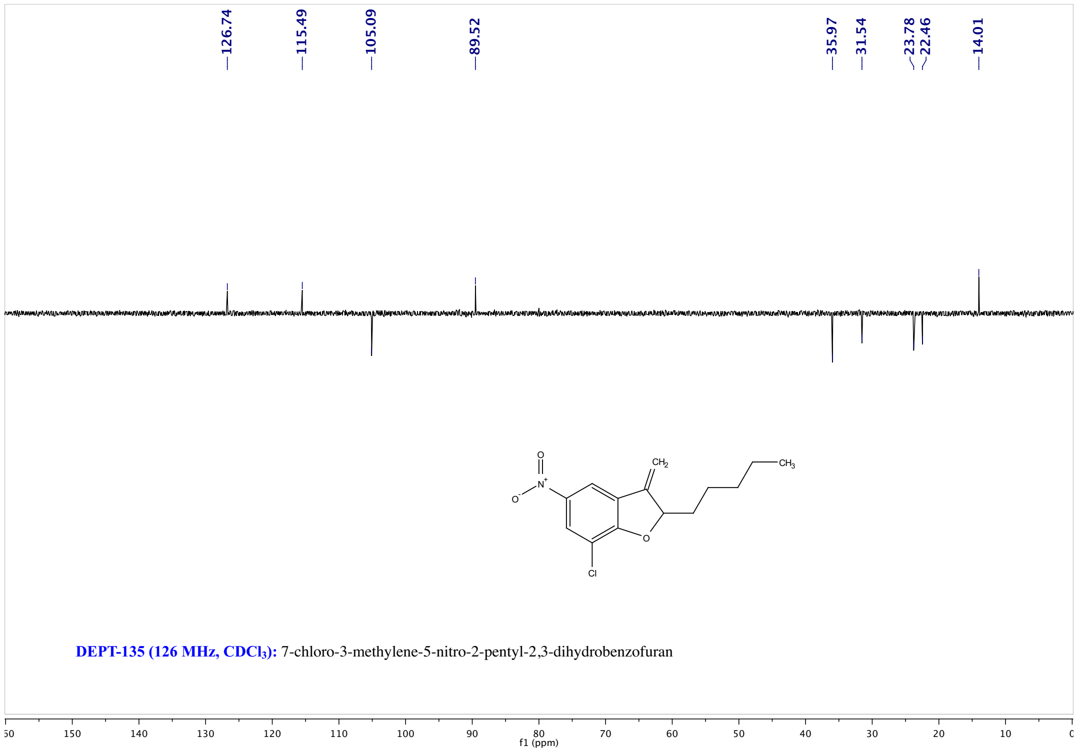

DEPT-135 (126 MHz, $CDCl_3$): 7-chloro-3-methylene-5-nitro-2-pentyl-2,3-dihydrobenzofuran

2D NMR: COSY

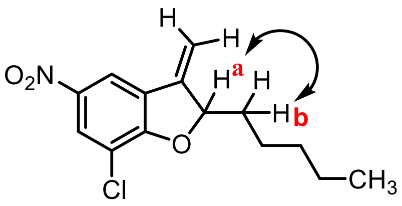

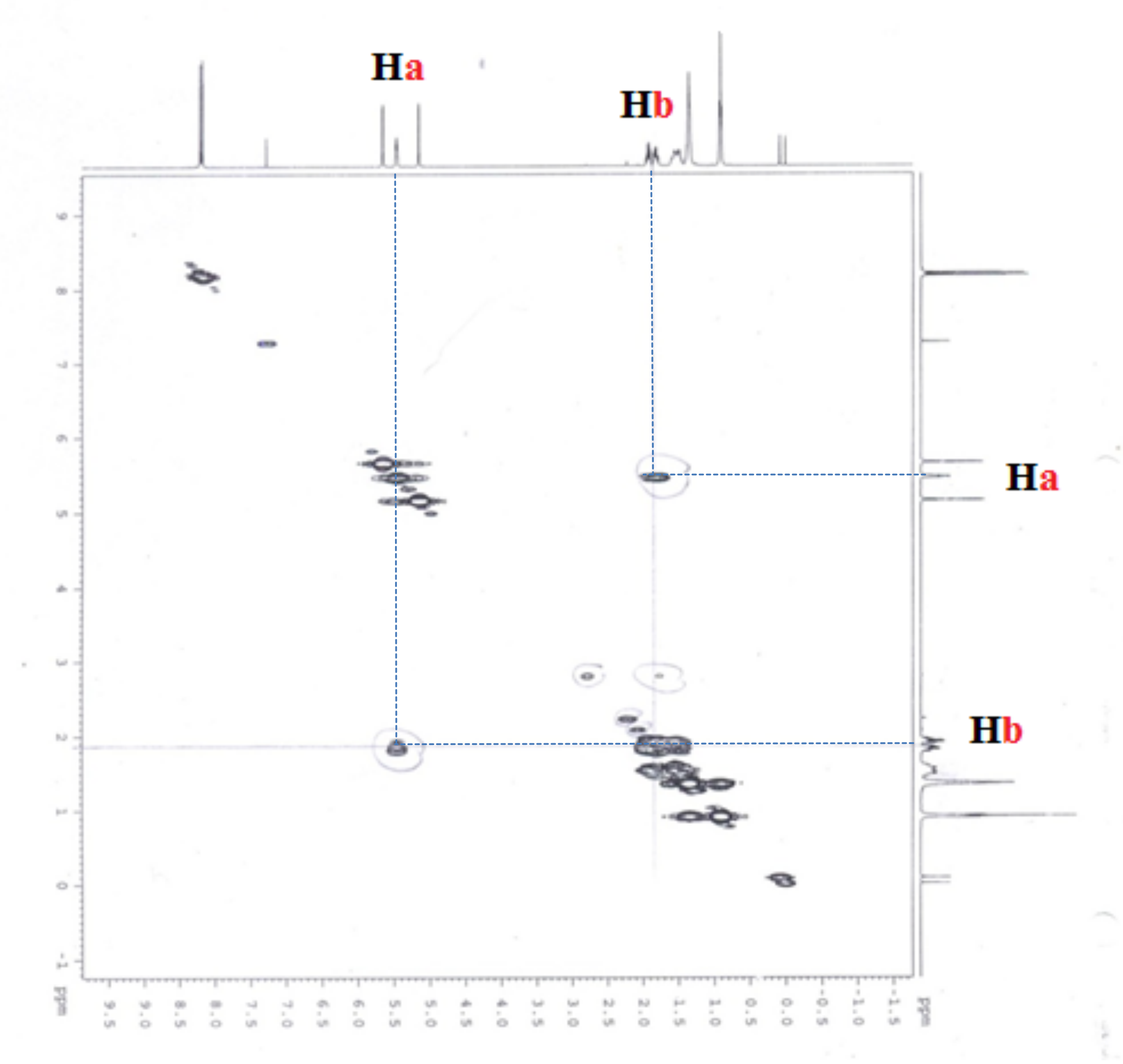

2D NMR: HMBC

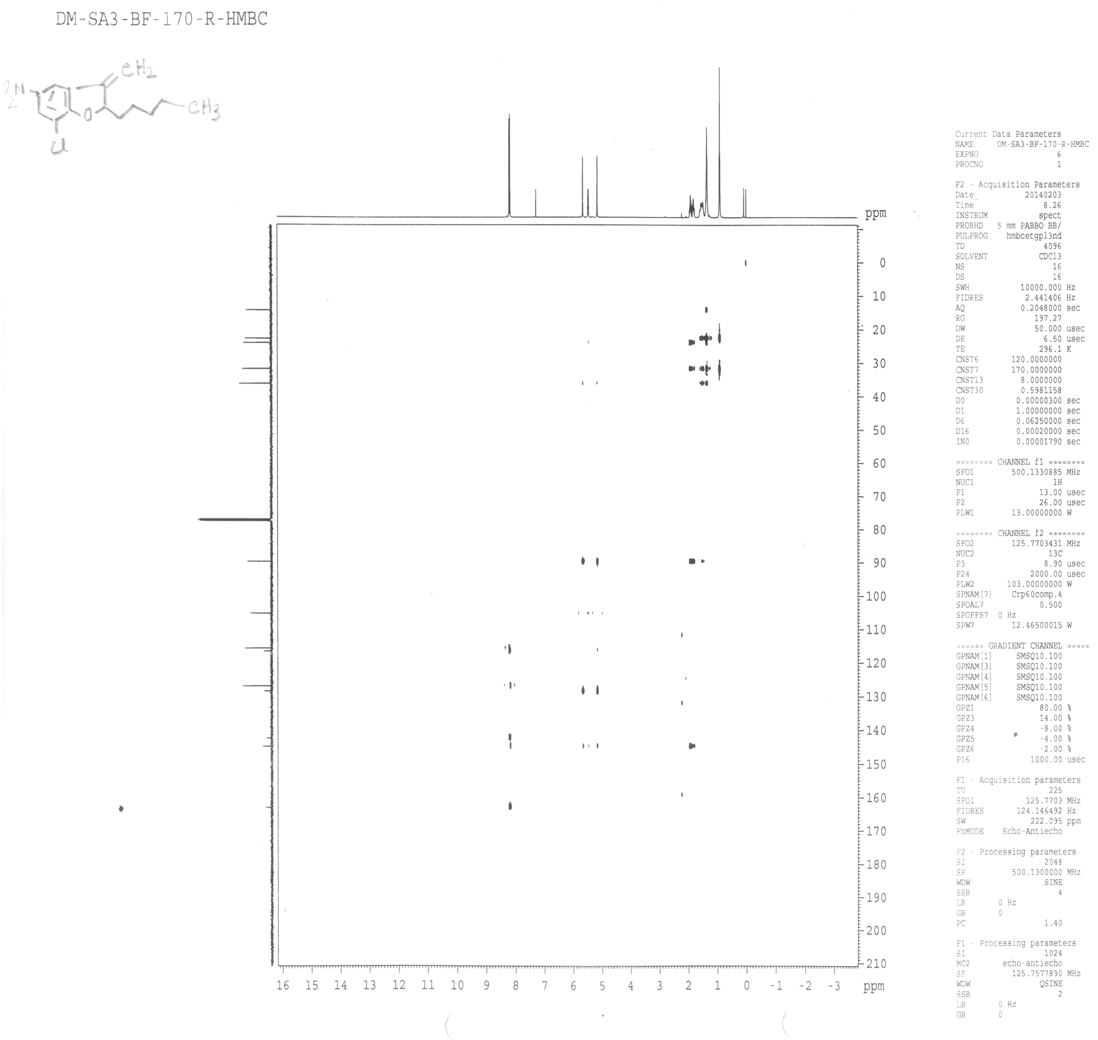
DM-SA3-BF-170-R-HMBC
CH2
CH3
Cl
ppm
0
10
20
30
40
50
60
70
80
90
100
110
120
130
140
150
160
170
180
190
200
210
16 15 14 13 12 11 10 9 8 7 6 5 4 3 2 1 0 -1 -2 -3 ppm
Current Data Parameters
NAME DM-SA3-BF-170-R-HMBC
EXPNO 6
PROCNO 1
F2 - Acquisition Parameters
Date_ 20140203
Time 8.26
INSTRUM spect
PROBHD 5 mm PABBO BB/
PULPROG hmbcetgpl3nd
TD 4096
SOLVENT CDCl3
NS 16
DS 16
SWH 10000.000 Hz
FIDRES 2.441406 Hz
AQ 0.2048000 sec
RG 197.27
DW 50.000 usec
DE 6.50 usec
TE 296.1 K
CNST6 120.0000000
CNST7 170.0000000
CNST13 8.0000000
CNST30 0.5981158
D0 0.00000300 sec
D1 1.00000000 sec
D6 0.06250000 sec
D16 0.00020000 sec
IN0 0.00001790 sec
======== CHANNEL f1 ========
SFO1 500.1330885 MHz
NUC1 1H
P1 13.00 usec
P2 26.00 usec
PLW1 13.00000000 W
======== CHANNEL f2 ========
SFO2 125.7703431 MHz
NUC2 13C
P3 8.90 usec
P24 2000.00 usec
PLW2 103.00000000 W
SPNAM[7] Crp60comp.4
SPOAL7 0.500
SPOFFS7 0 Hz
SPW7 12.46500015 W
====== GRADIENT CHANNEL =====
GPNAM[1] SMSQ10.100
GPNAM[3] SMSQ10.100
GPNAM[4] SMSQ10.100
GPNAM[5] SMSQ10.100
GPNAM[6] SMSQ10.100
GPZ1 80.00 %
GPZ3 14.00 %
GPZ4 -8.00 %
GPZ5 -4.00 %
GPZ6 -2.00 %
P16 1000.00 usec
F1 - Acquisition parameters
TD 225
SFO1 125.7703 MHz
FIDRES 124.146492 Hz
SW 222.095 ppm
FnMODE Echo-Antiecho
F2 - Processing parameters
SI 2048
SF 500.1300000 MHz
WDW SINE
SSB 4
LB 0 Hz
GB 0
PC 1.40
F1 - Processing parameters
SI 1024
MC2 echo-antiecho
SF 125.7577890 MHz
WDW QSINE
SSB 2
LB 0 Hz
GB 0

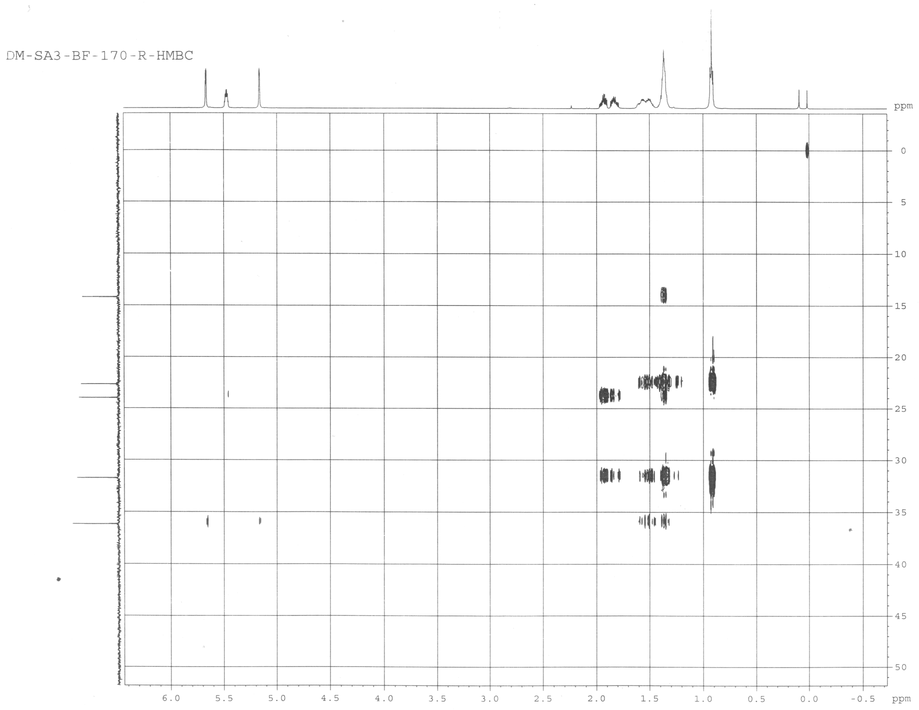
DM-SA3-BF-170-R-HMBC
ppm
0
5
10
15
20
25
30
35
40
45
50
6.0
5.5
5.0
4.5
4.0
3.5
3.0
2.5
2.0
1.5
1.0
0.5
0.0
-0.5
ppm

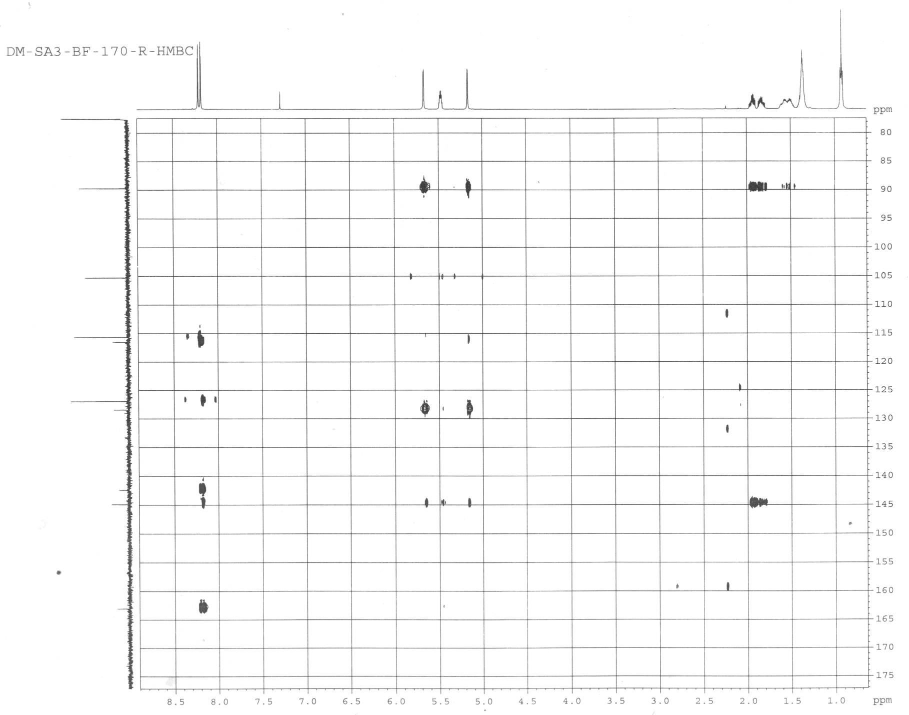
DM-SA3-BF-170-R-HMBC
ppm
80
85
90
95
100
105
110
115
120
125
130
135
140
145
150
155
160
165
170
175
8.5
8.0
7.5
7.0
6.5
6.0
5.5
5.0
4.5
4.0
3.5
3.0
2.5
2.0
1.5
1.0
ppm

2D NMR: HSQC

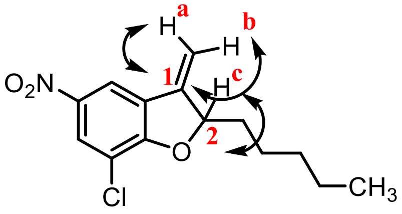

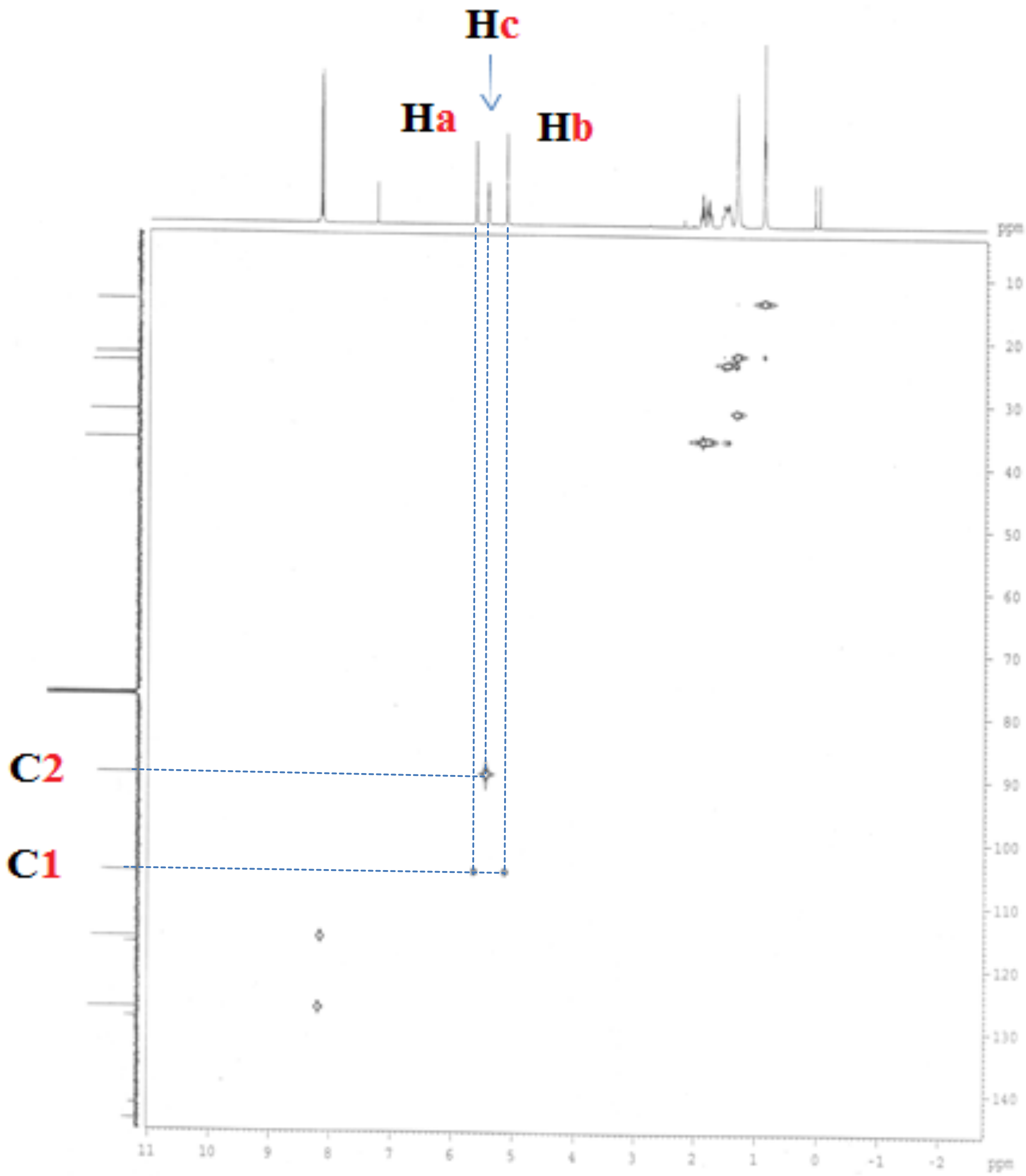

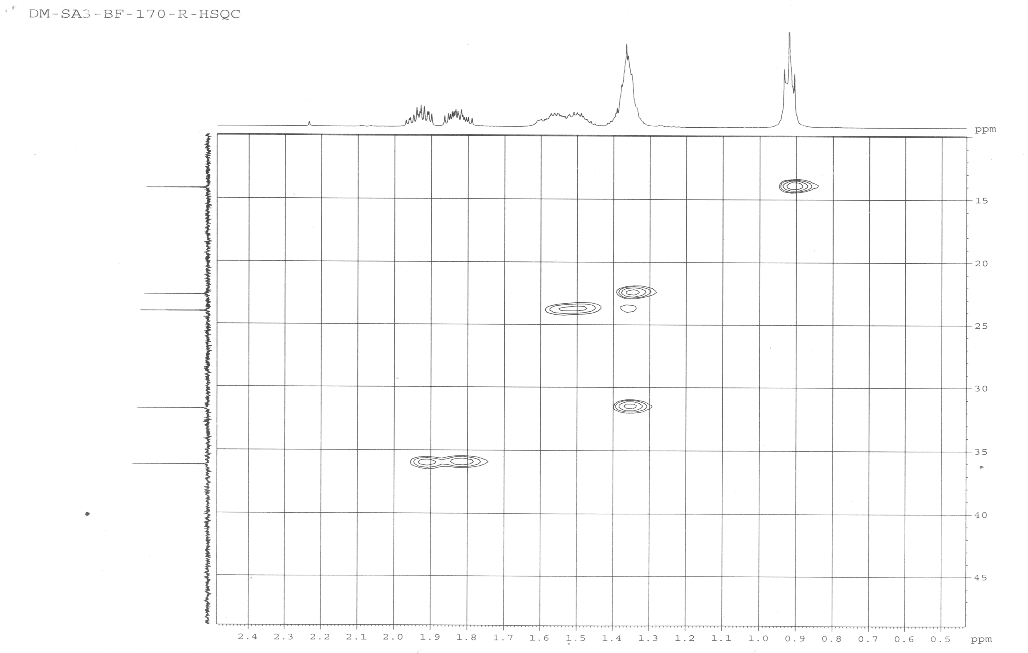
DM-SA3-BF-170-R-HSQC
ppm

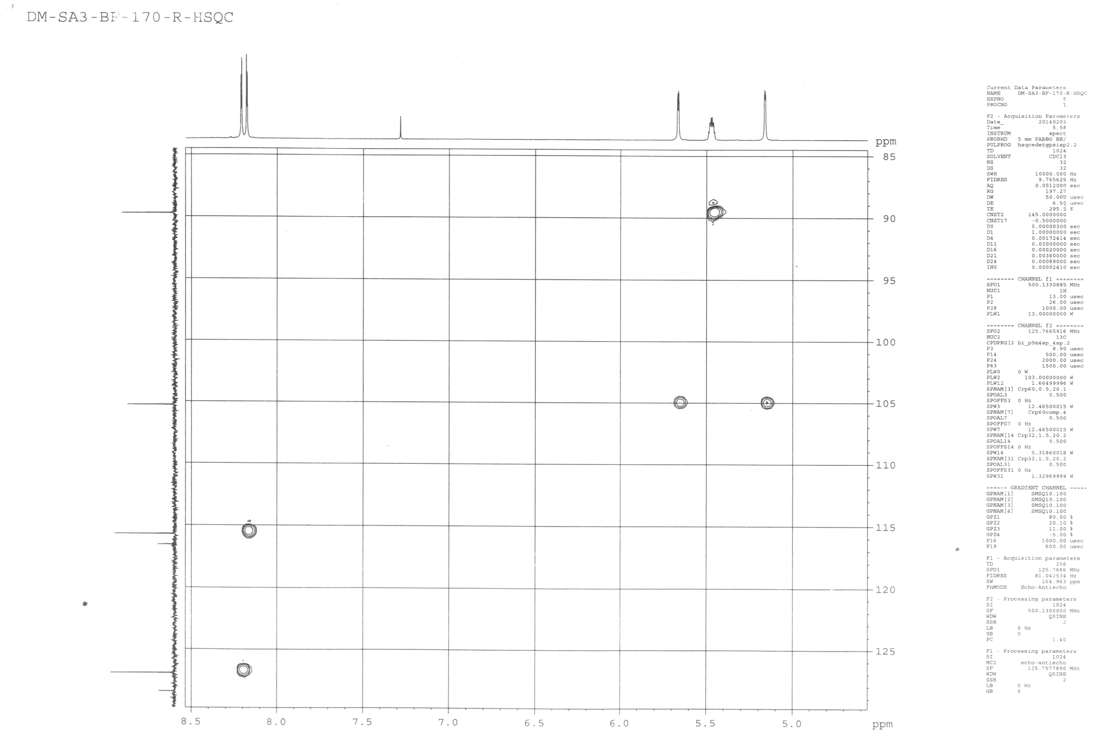
DM-SA3-BF-170-R-HSQC
ppm

Table 1, Entry 3b:

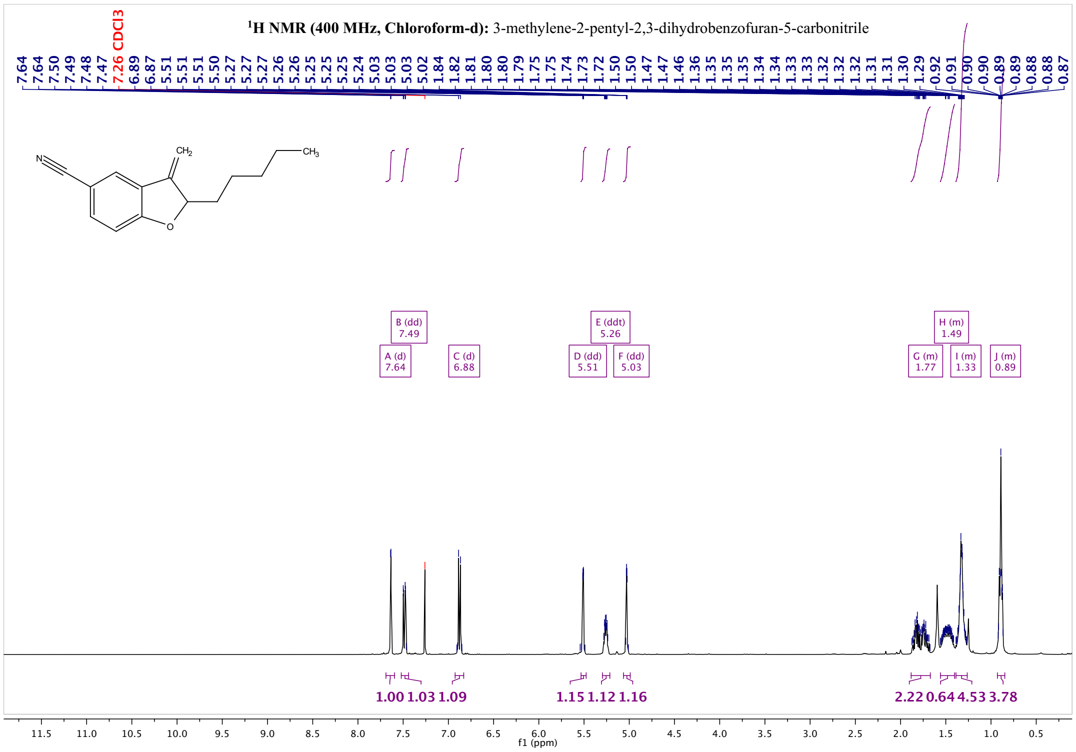

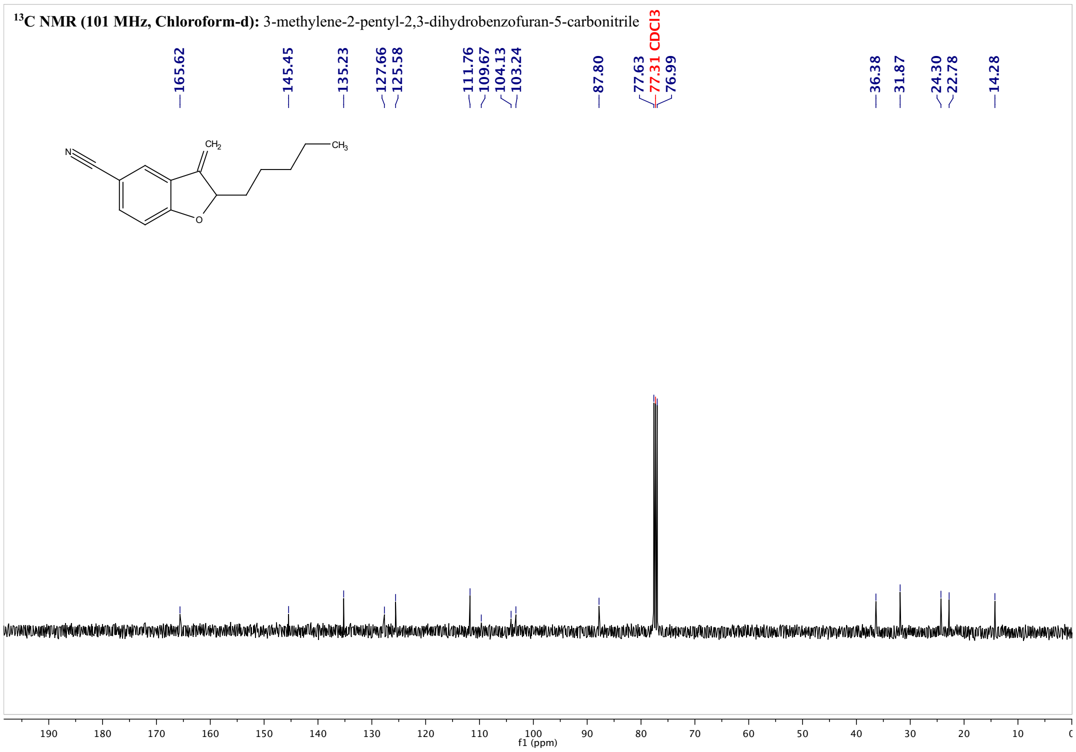

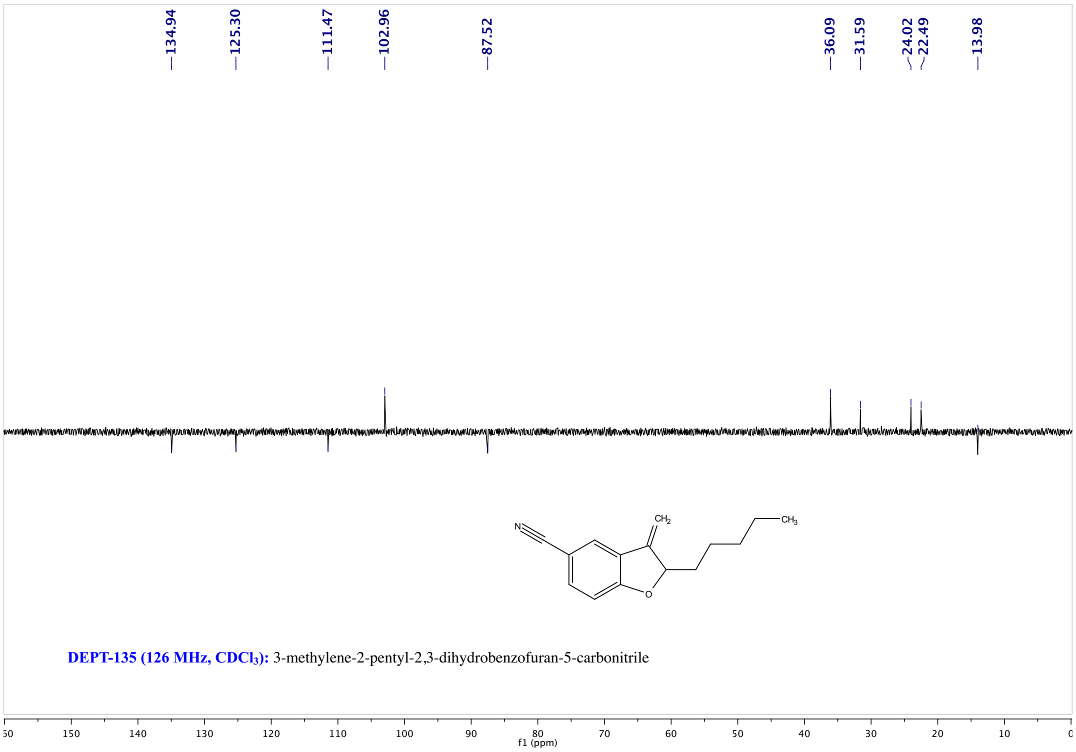

DEPT-135 (126 MHz, $CDCl_3$): 3-methylene-2-pentyl-2,3-dihydrobenzofuran-5-carbonitrile

Table 1, Entry 3c:

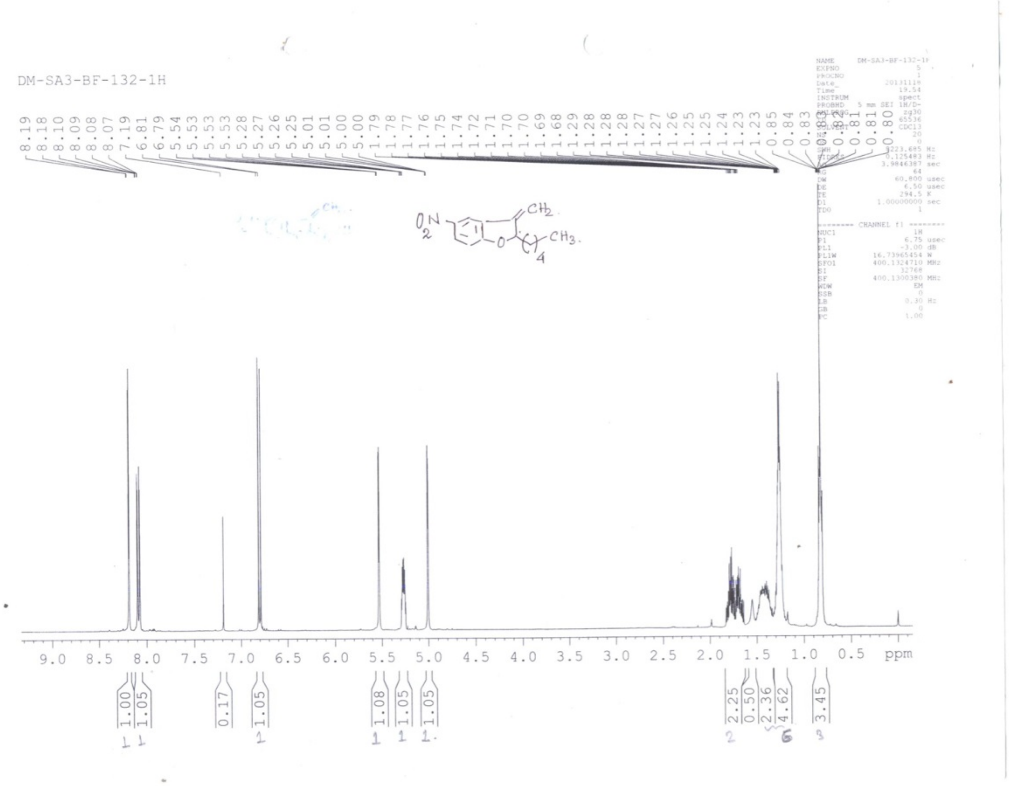

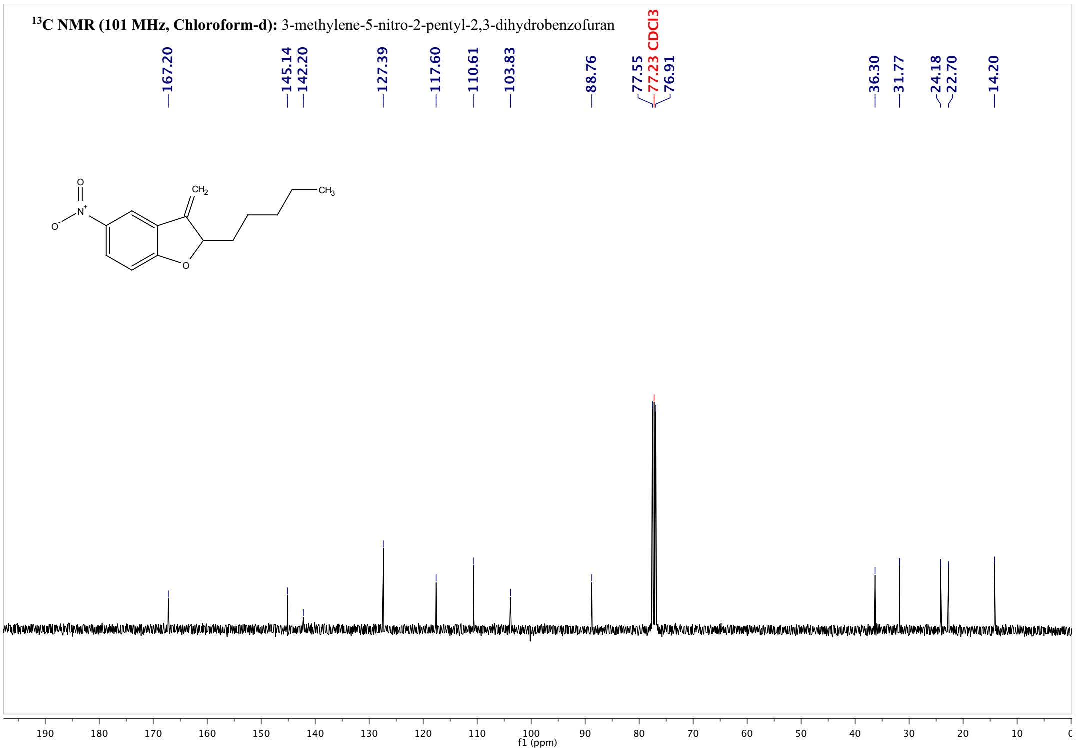

Table 1, Entry 3d:

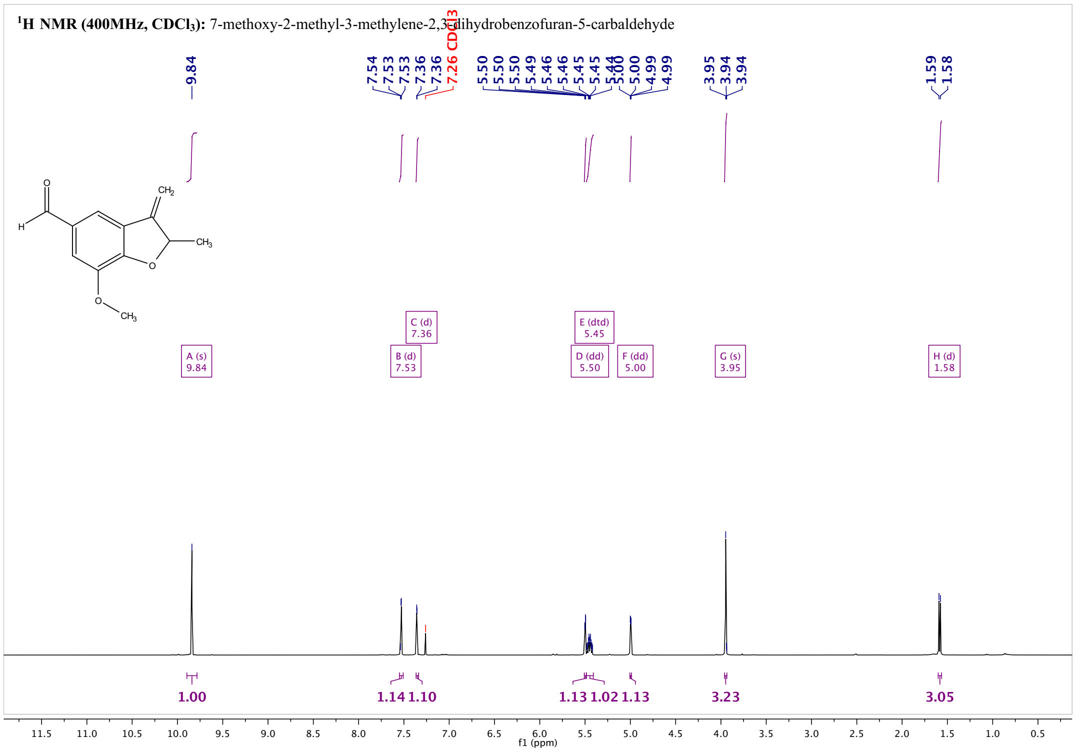

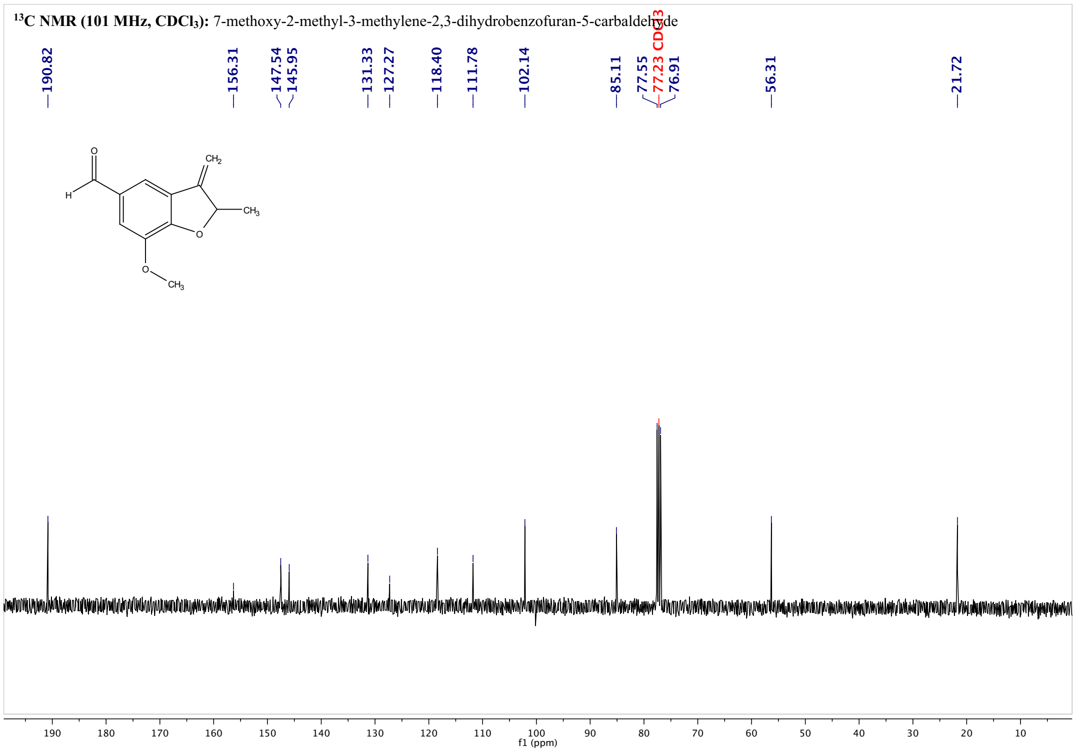

Table 1, Entry 4e:

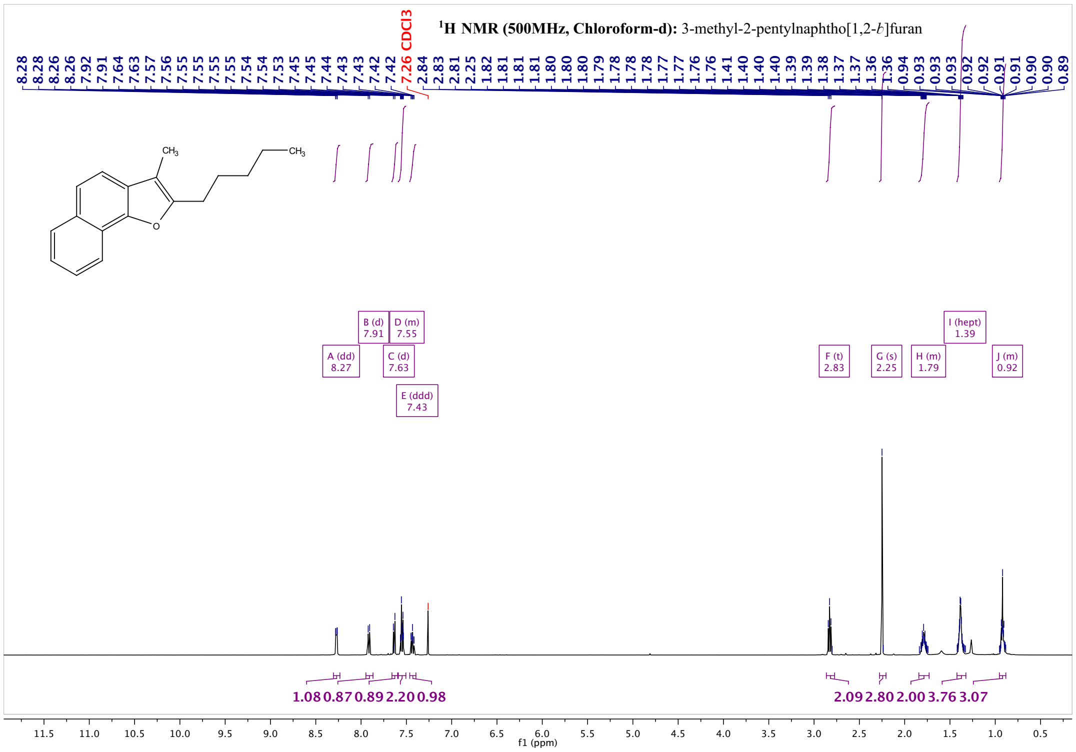

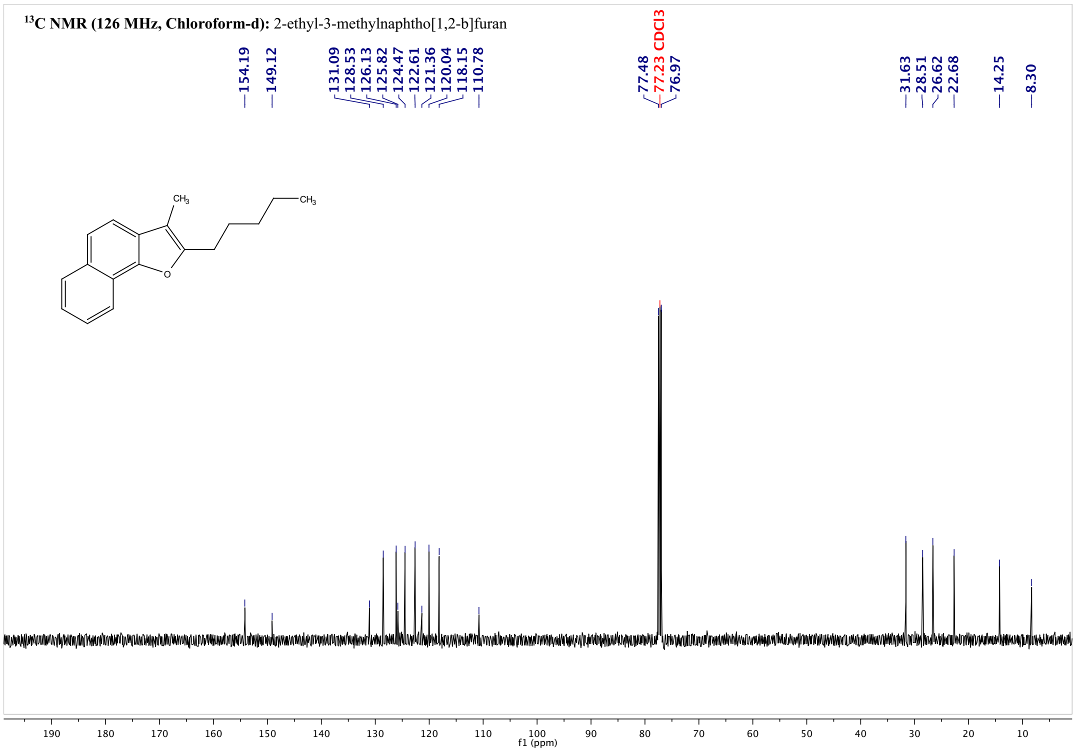

^{13}C NMR (126 MHz, Chloroform-d): 2-ethyl-3-methylnaphtho[1,2-b]furan

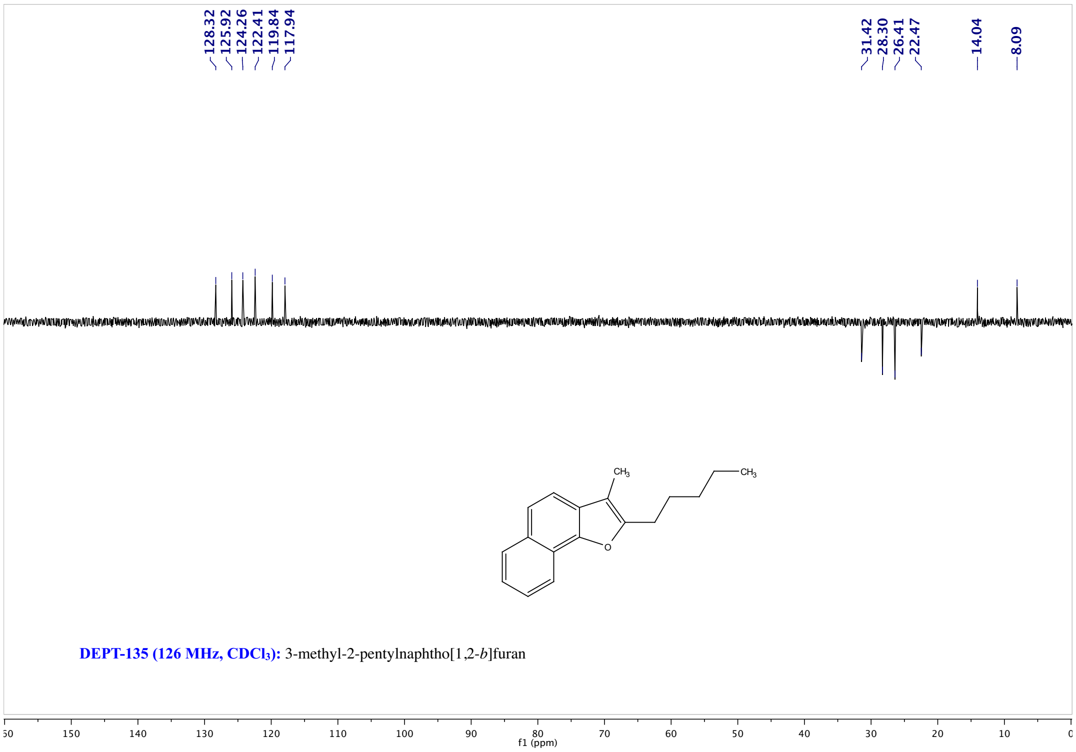

DEPT-135 (126 MHz, $CDCl_3$): 3-methyl-2-pentylnaphtho[1,2-*b*]furan

Table 1, Entry 4f:

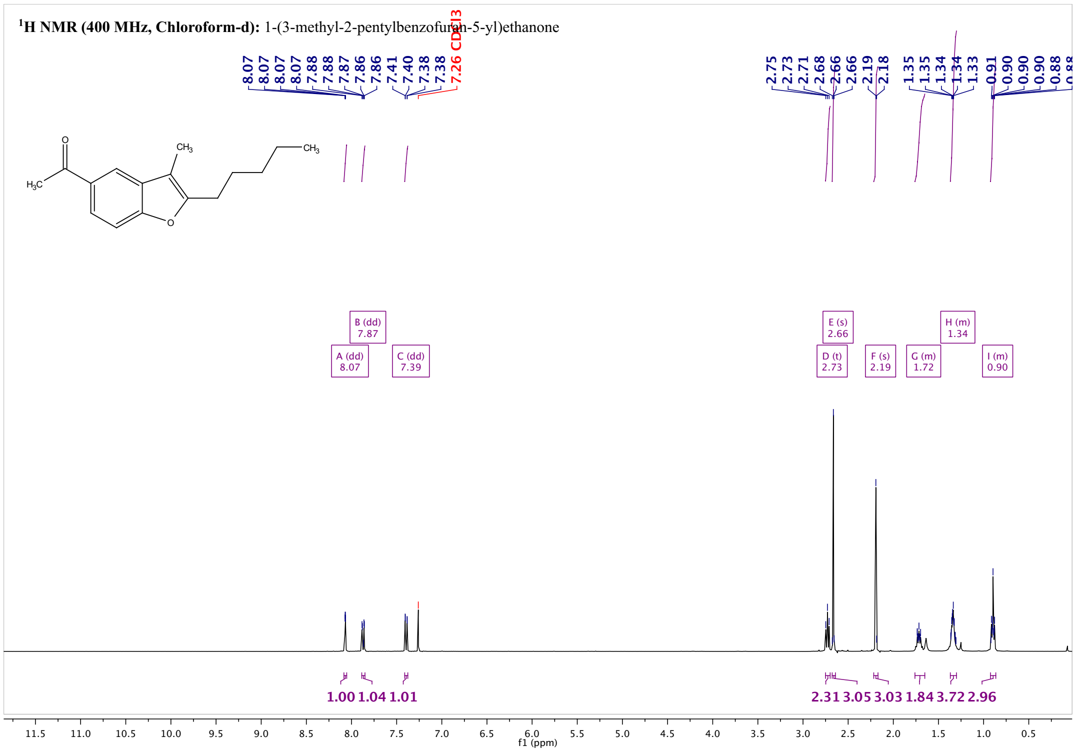

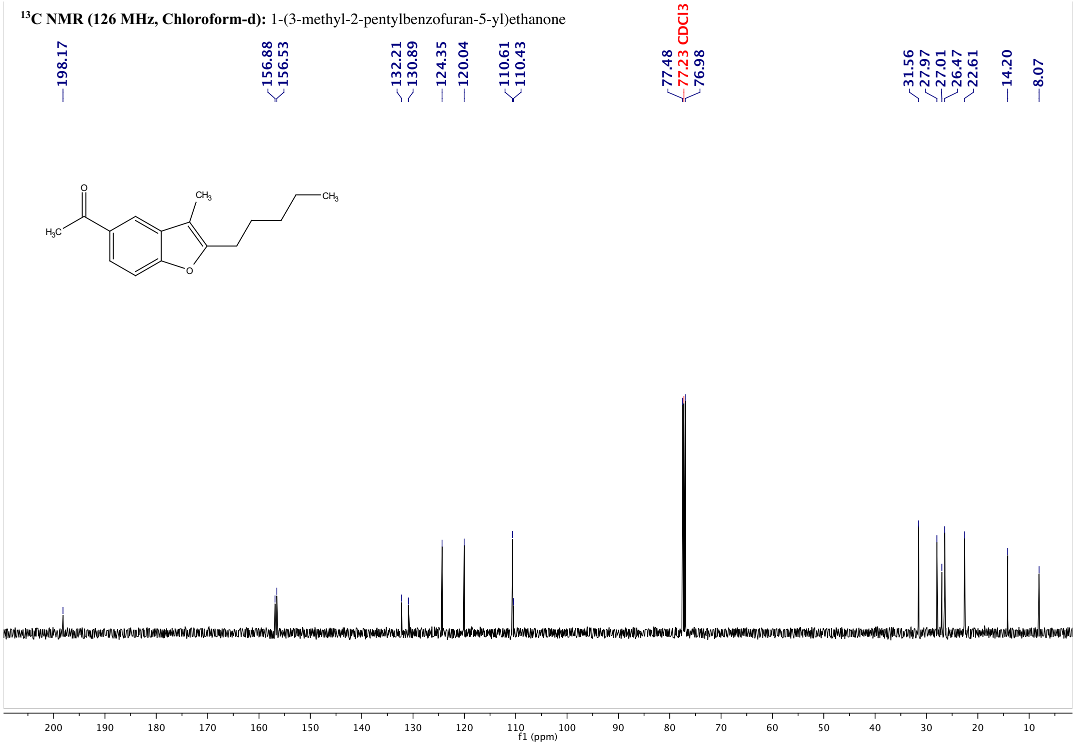

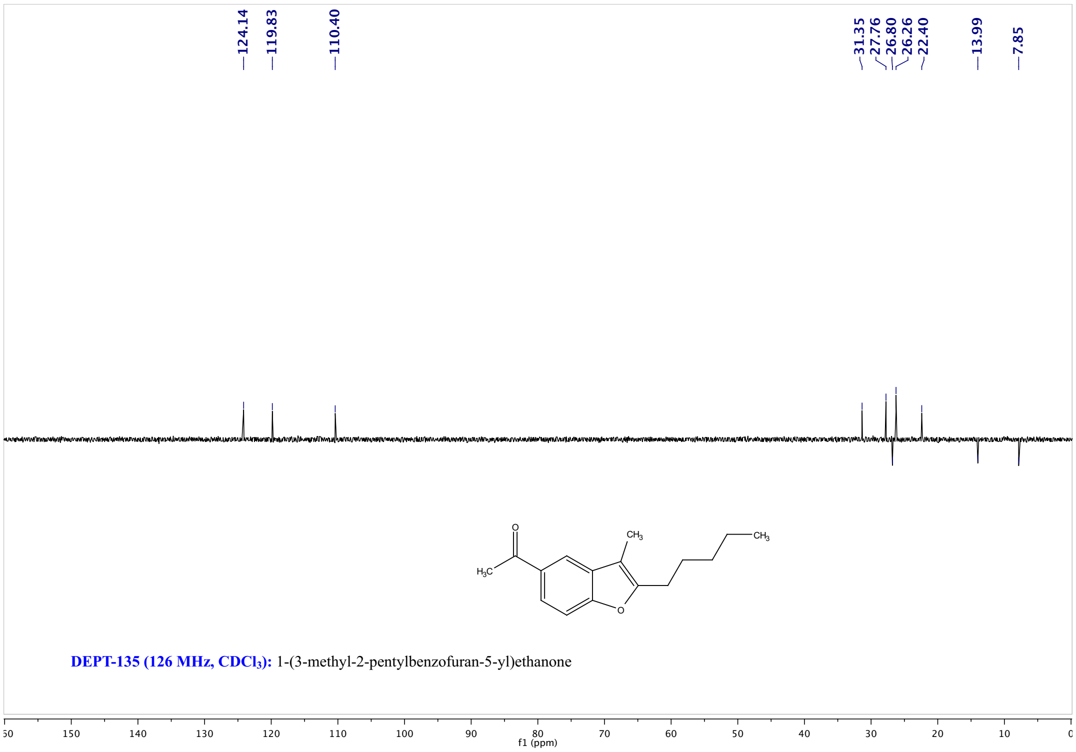

Table 1, Entry 4g:

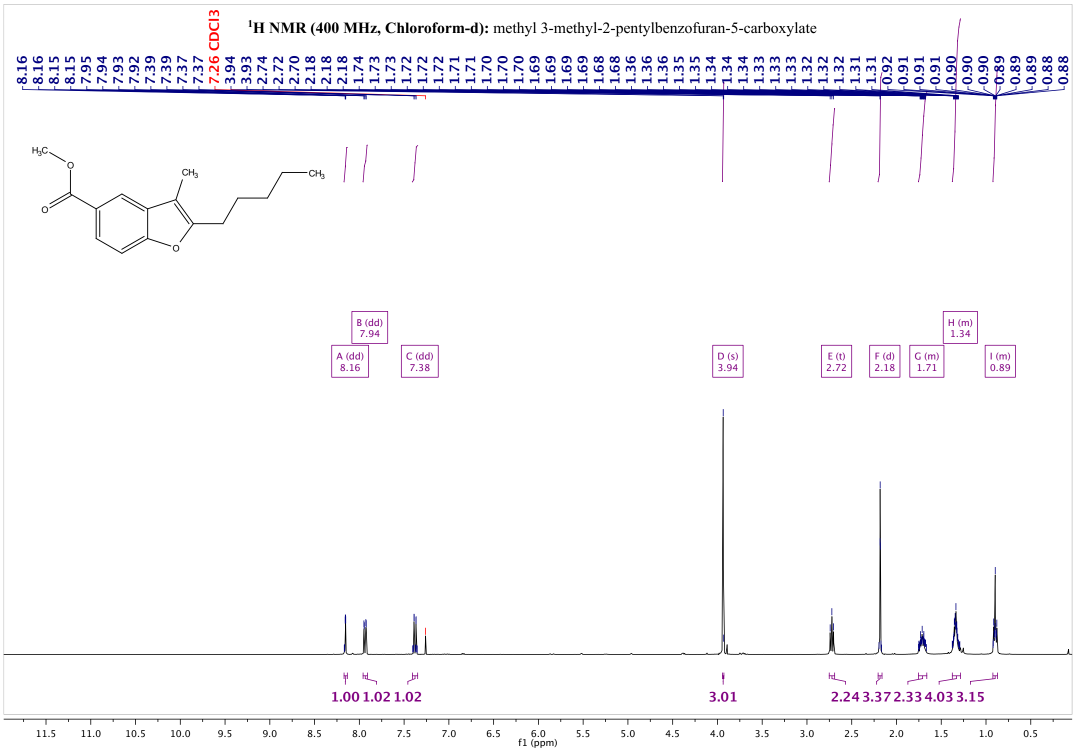

^{13}C NMR (101 MHz, Chloroform-d): methyl 3-methyl-2-pentylbenzofuran-5-carboxylate

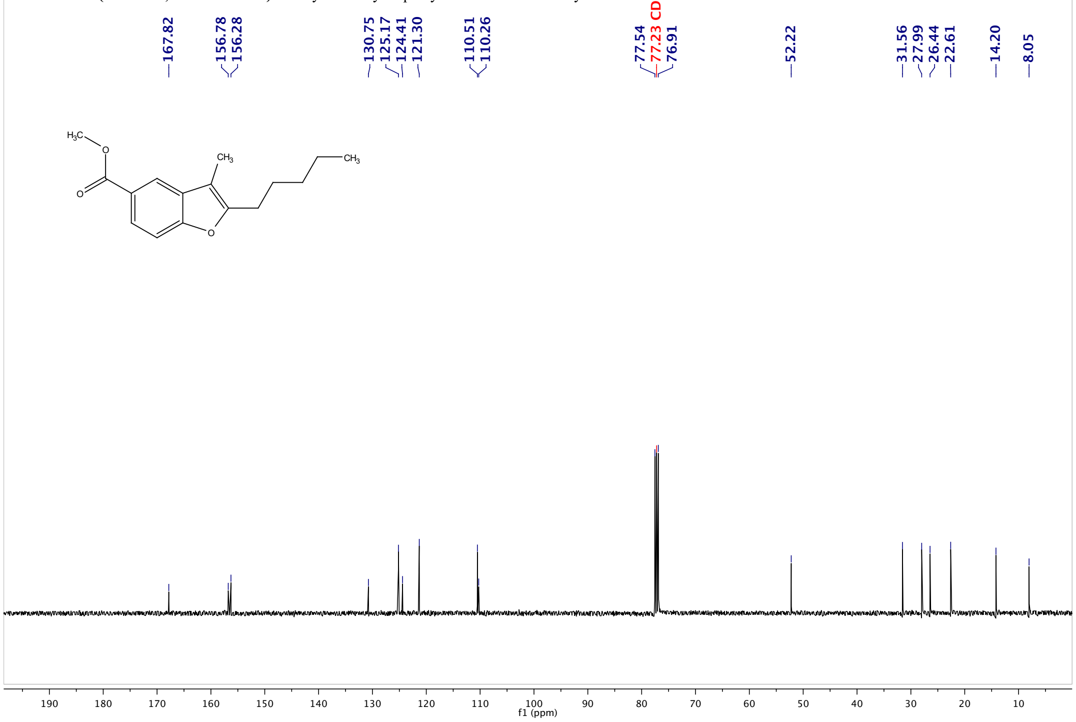

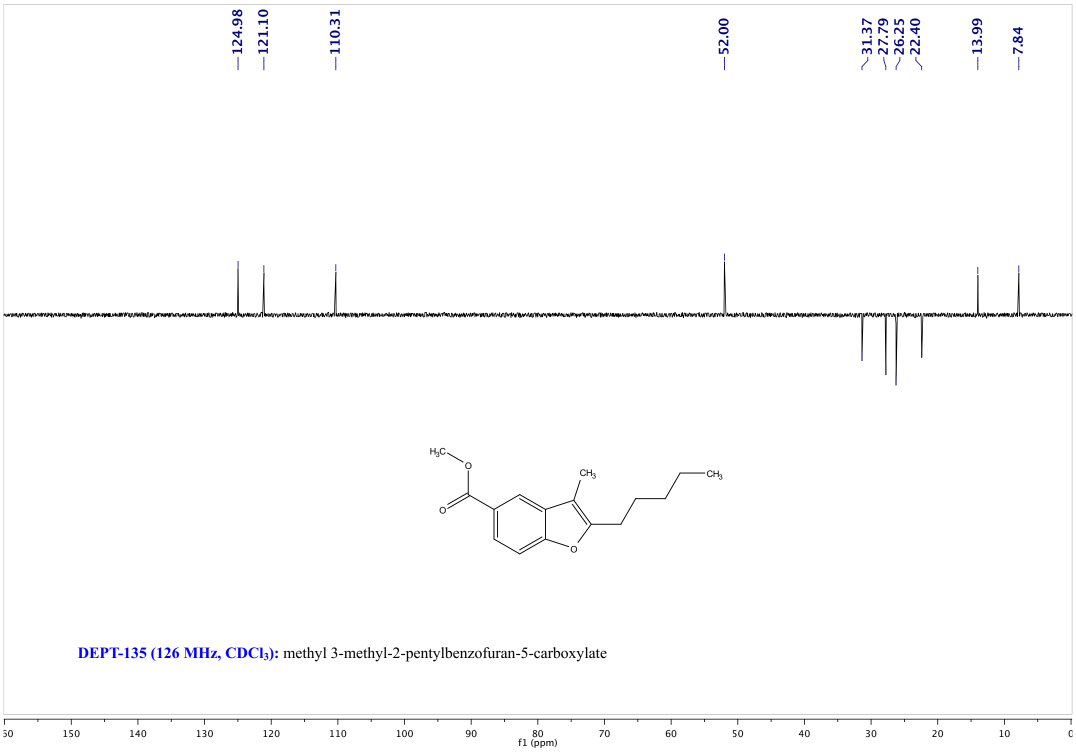

DEPT-135 (126 MHz, $CDCl_3$): methyl 3-methyl-2-pentylbenzofuran-5-carboxylate

Table 1, Entry 4h:

^{1}H NMR (400MHz, Chloroform-d): 3-methyl-2-pentyl-5-phenylbenzofuran

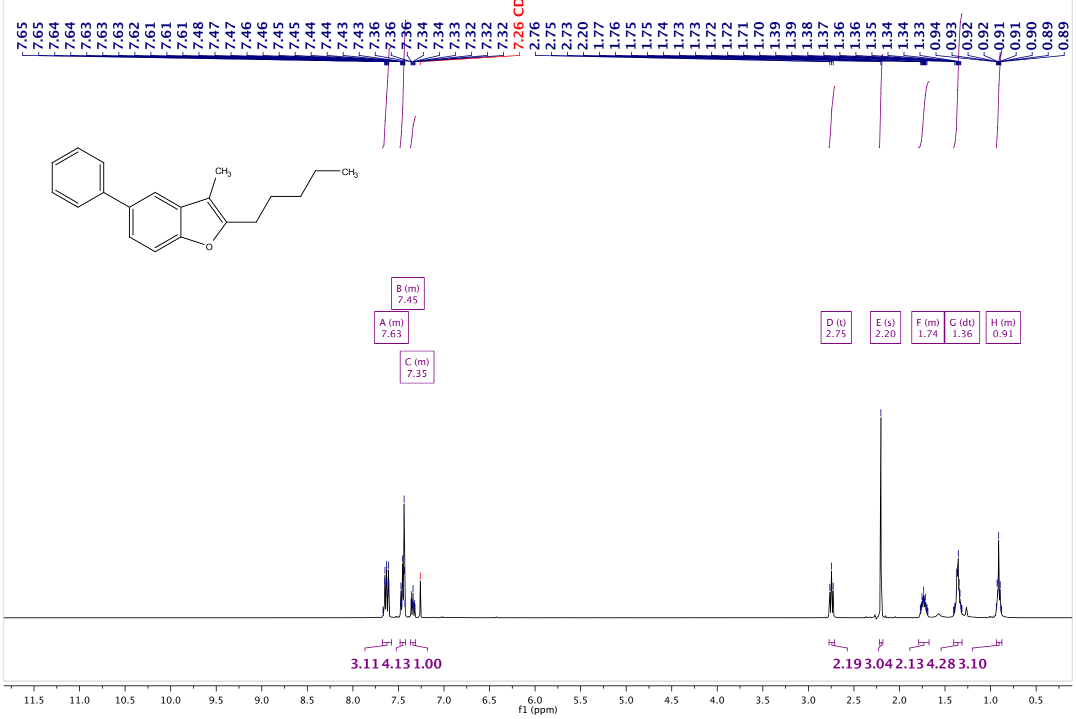

^{13}C NMR (101 MHz, $CDCl_3$): 3-methyl-2-pentyl-5-phenylbenzofuran

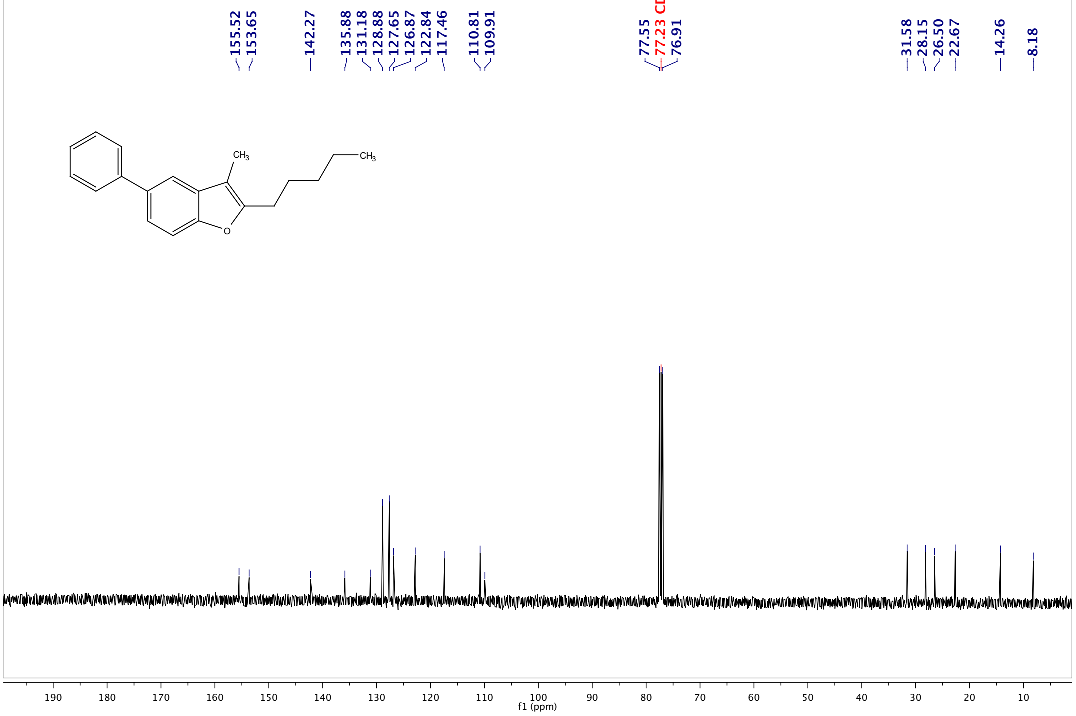

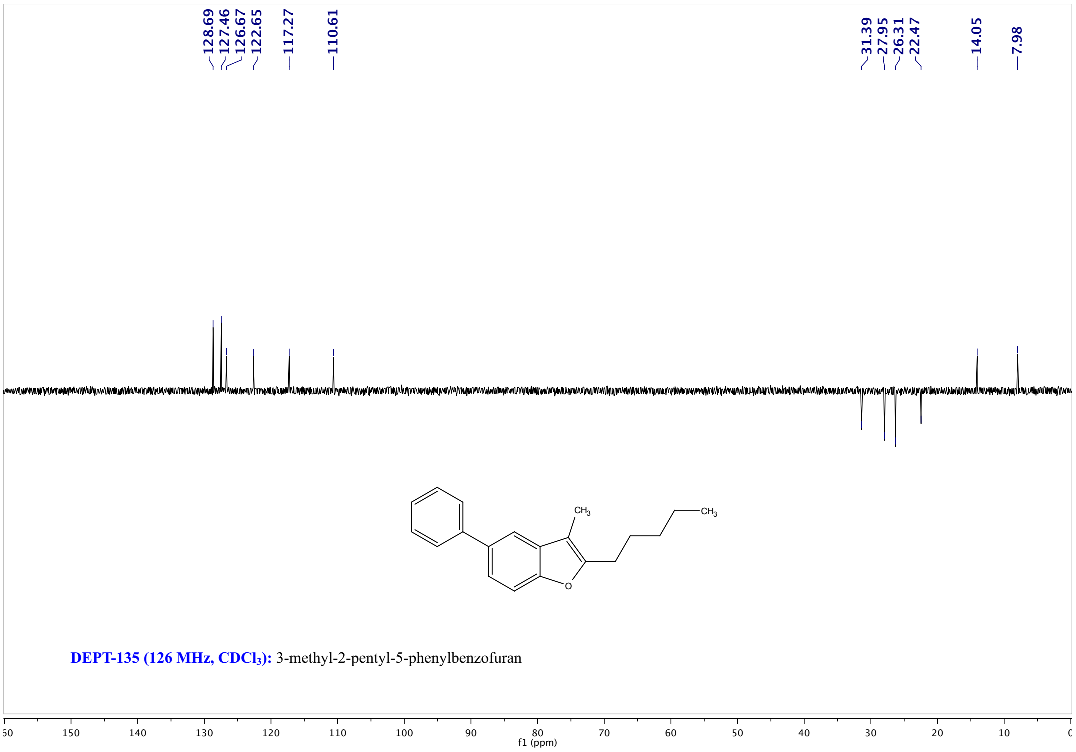

DEPT-135 (126 MHz, CDCl$_3$): 3-methyl-2-pentyl-5-phenylbenzofuran

Table 1, Entry 4i:

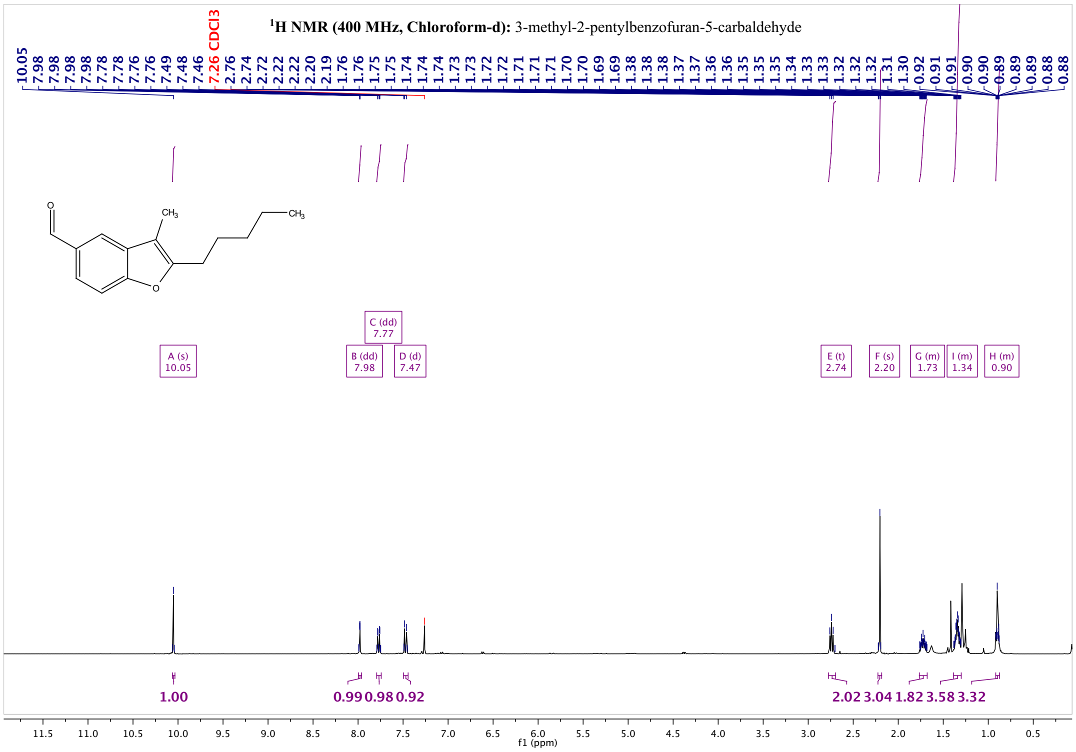

^{1}H NMR (400 MHz, Chloroform-d): 3-methyl-2-pentylbenzofuran-5-carbaldehyde

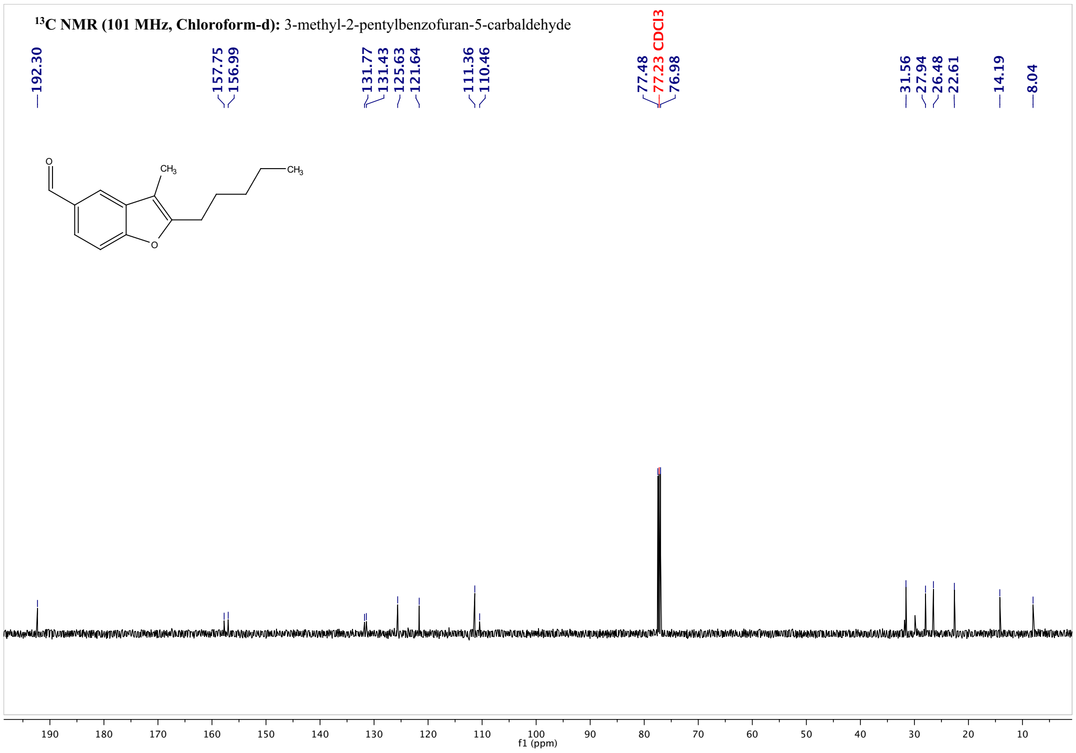

^{13}C NMR (101 MHz, Chloroform-d): 3-methyl-2-pentylbenzofuran-5-carbaldehyde
192.30
157.75
156.99
131.77
131.43
125.63
121.64
111.36
110.46
77.48
77.23 CDCl3
76.98
31.56
27.94
26.48
22.61
14.19
8.04
O
CH3
CH3
O
f1 (ppm)

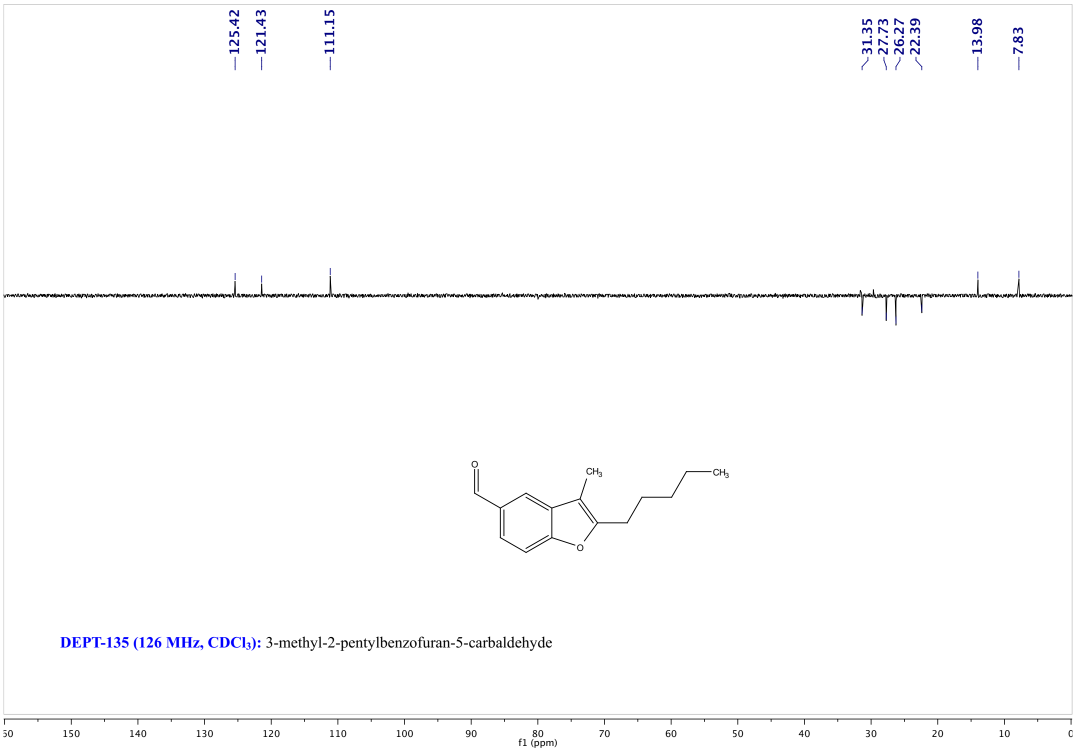

125.42
121.43
111.15
31.35
27.73
26.27
22.39
13.98
7.83
O
CH3
CH3
O
DEPT-135 (126 MHz, $CDCl_3$): 3-methyl-2-pentylbenzofuran-5-carbaldehyde
f1 (ppm)

Table 1, Entry 4j:

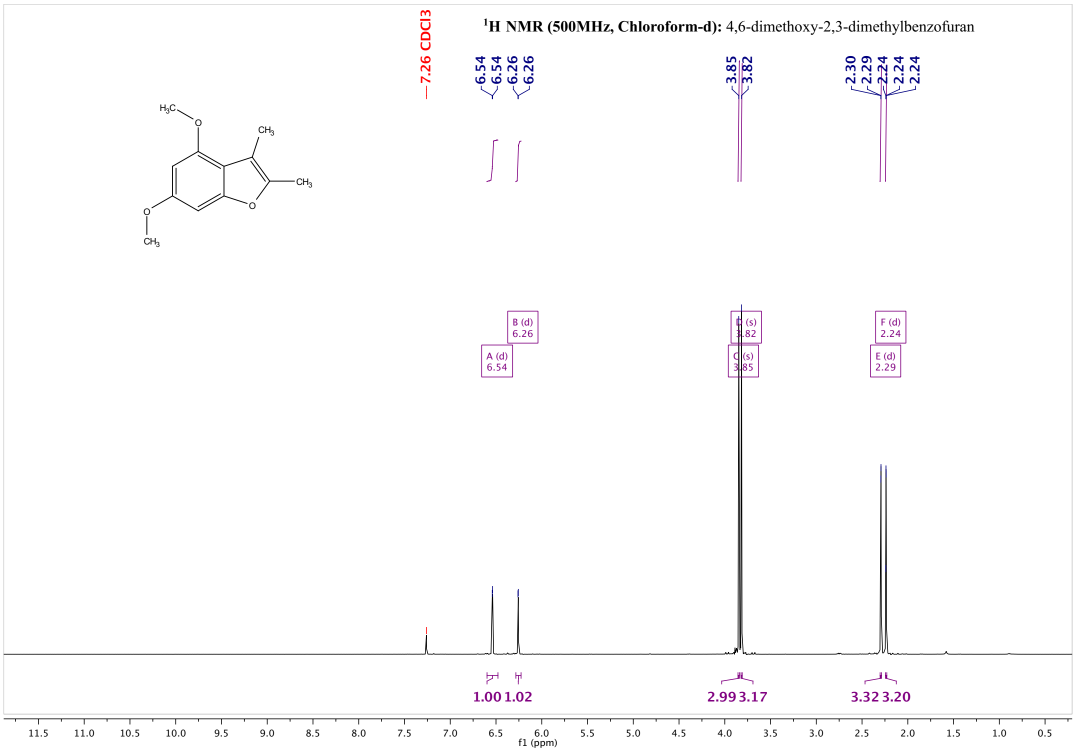

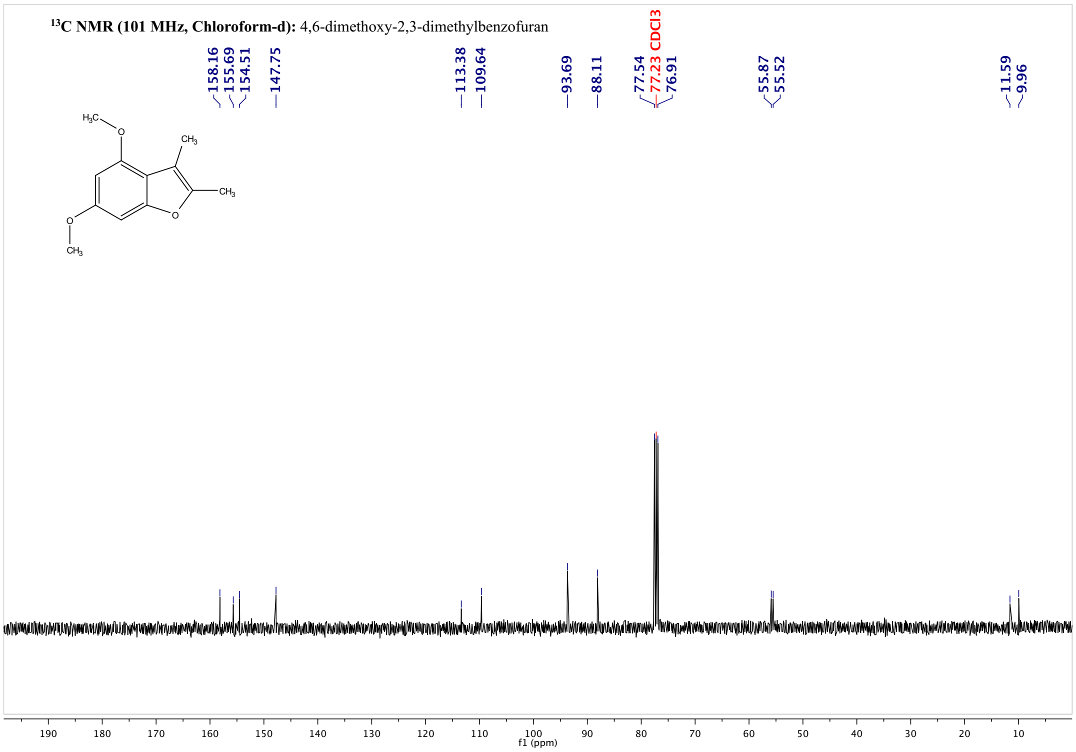

Table 1, Entry 4k:

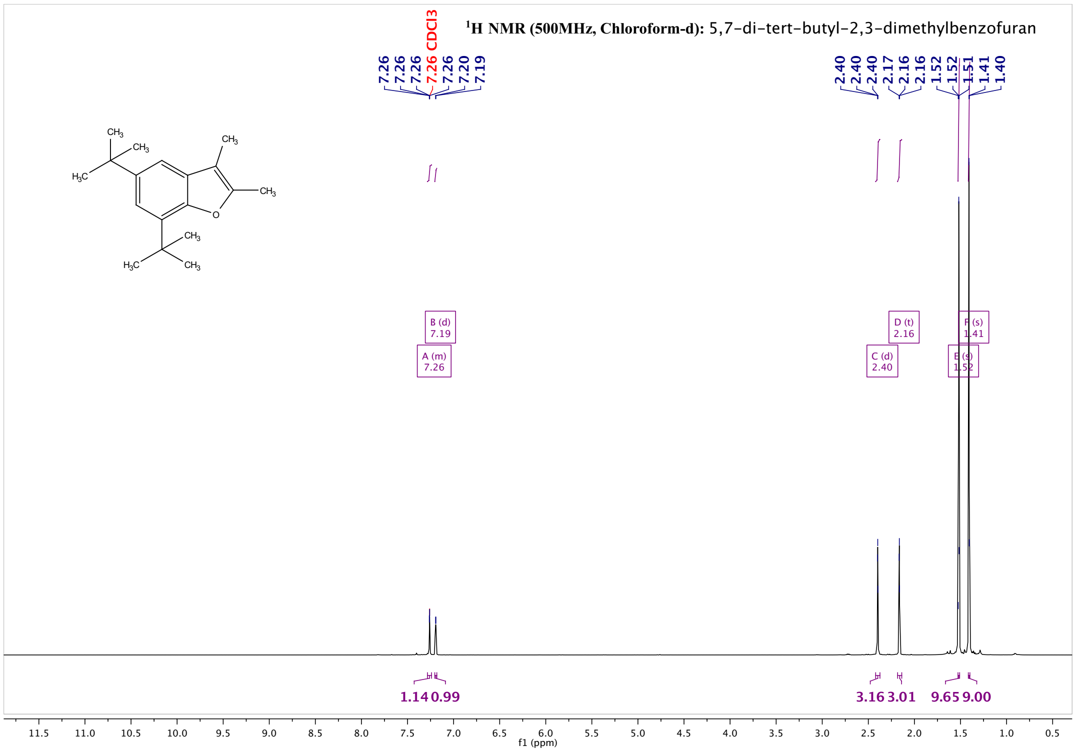

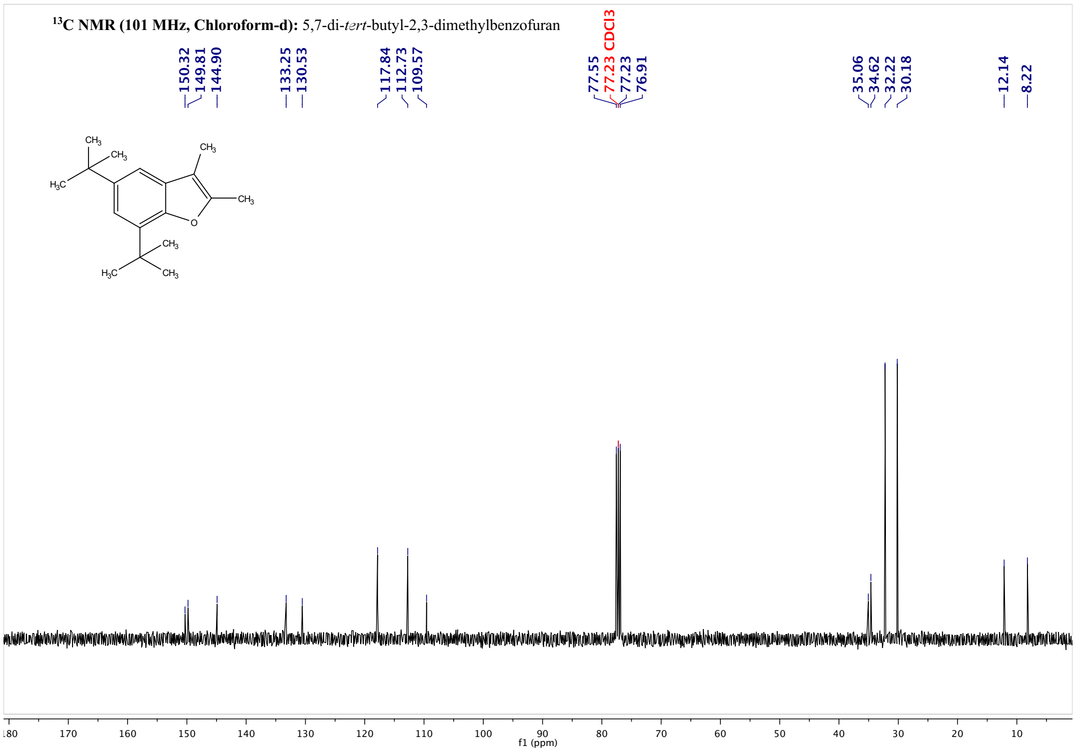

Scheme 3, $R_1 = R_2 = CH_3$:

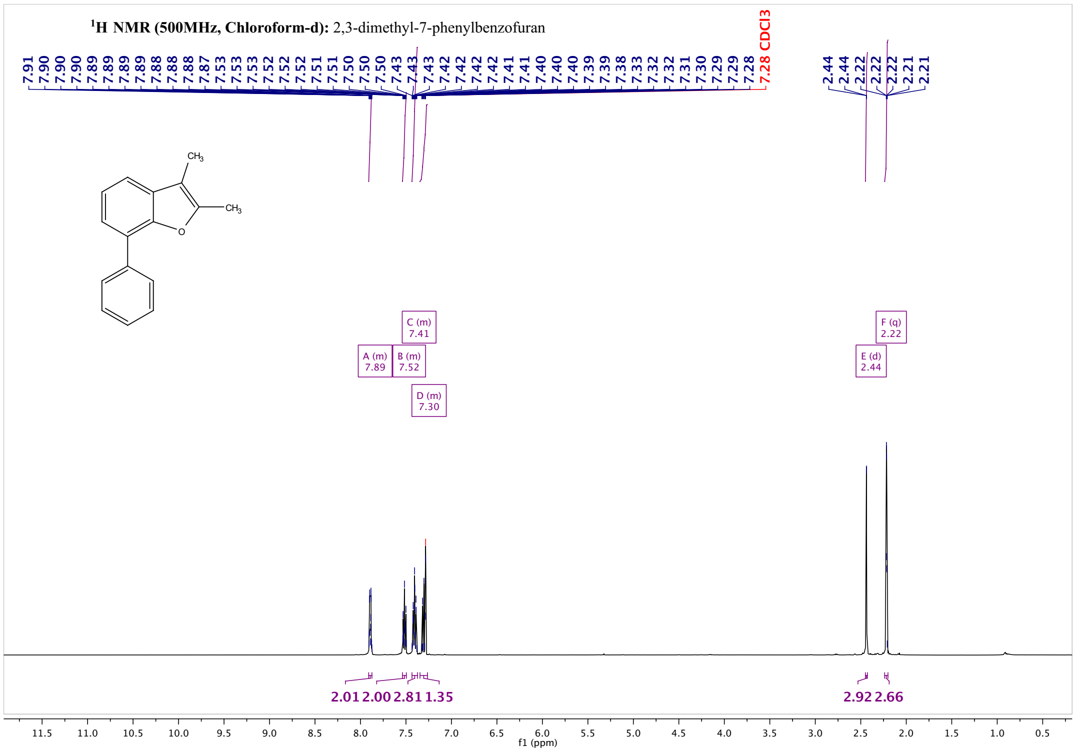

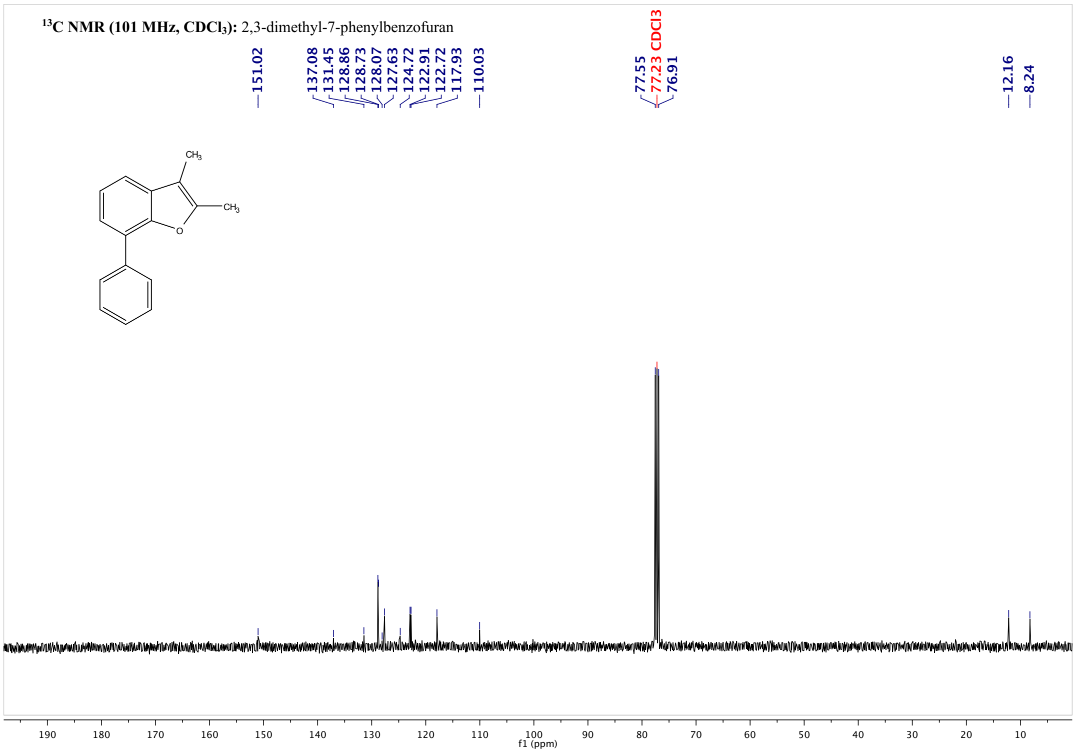

Scheme 3, R_1=CH_3, R_2= $(CH_2)_6CH_3$:

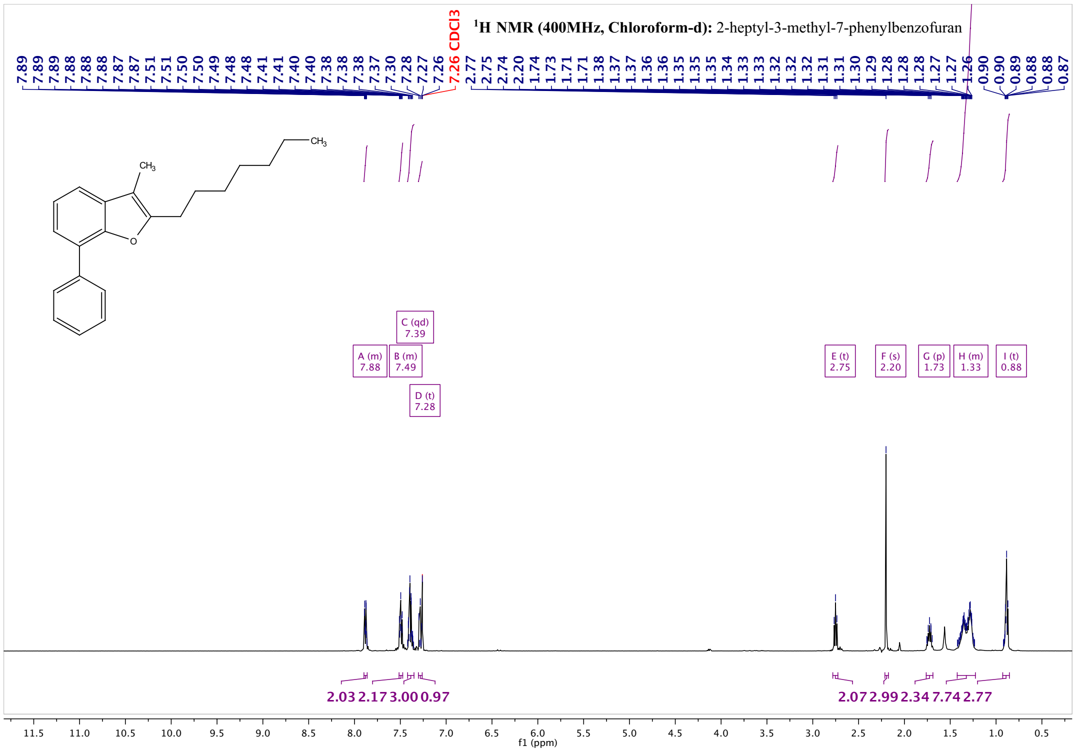

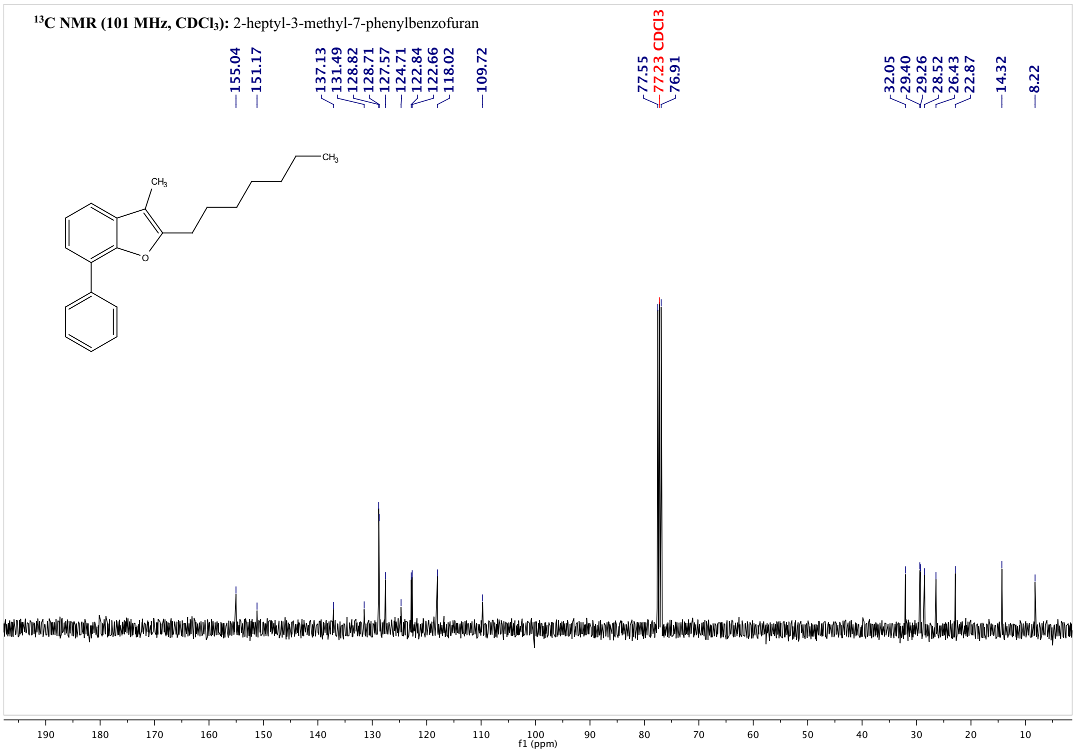

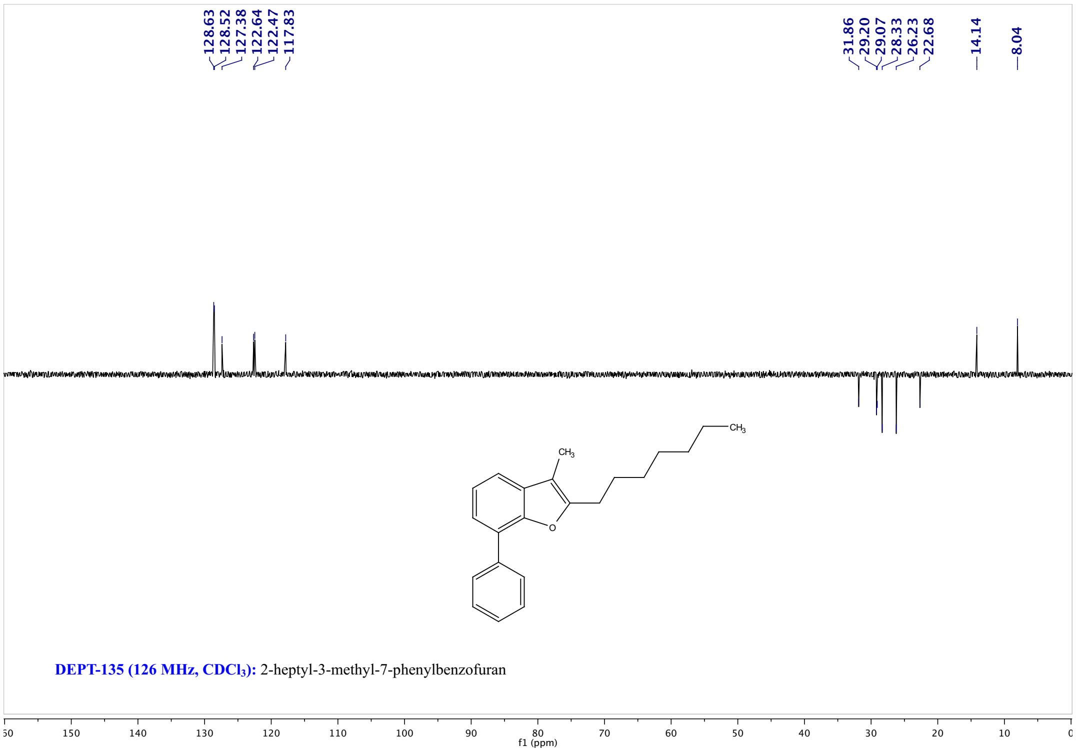

DEPT-135 (126 MHz, $CDCl_3$): 2-heptyl-3-methyl-7-phenylbenzofuran

2D NMR: COSY

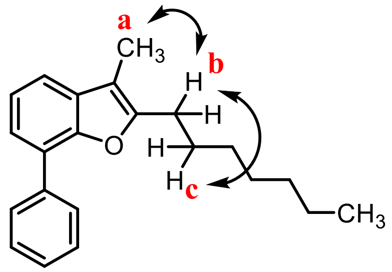

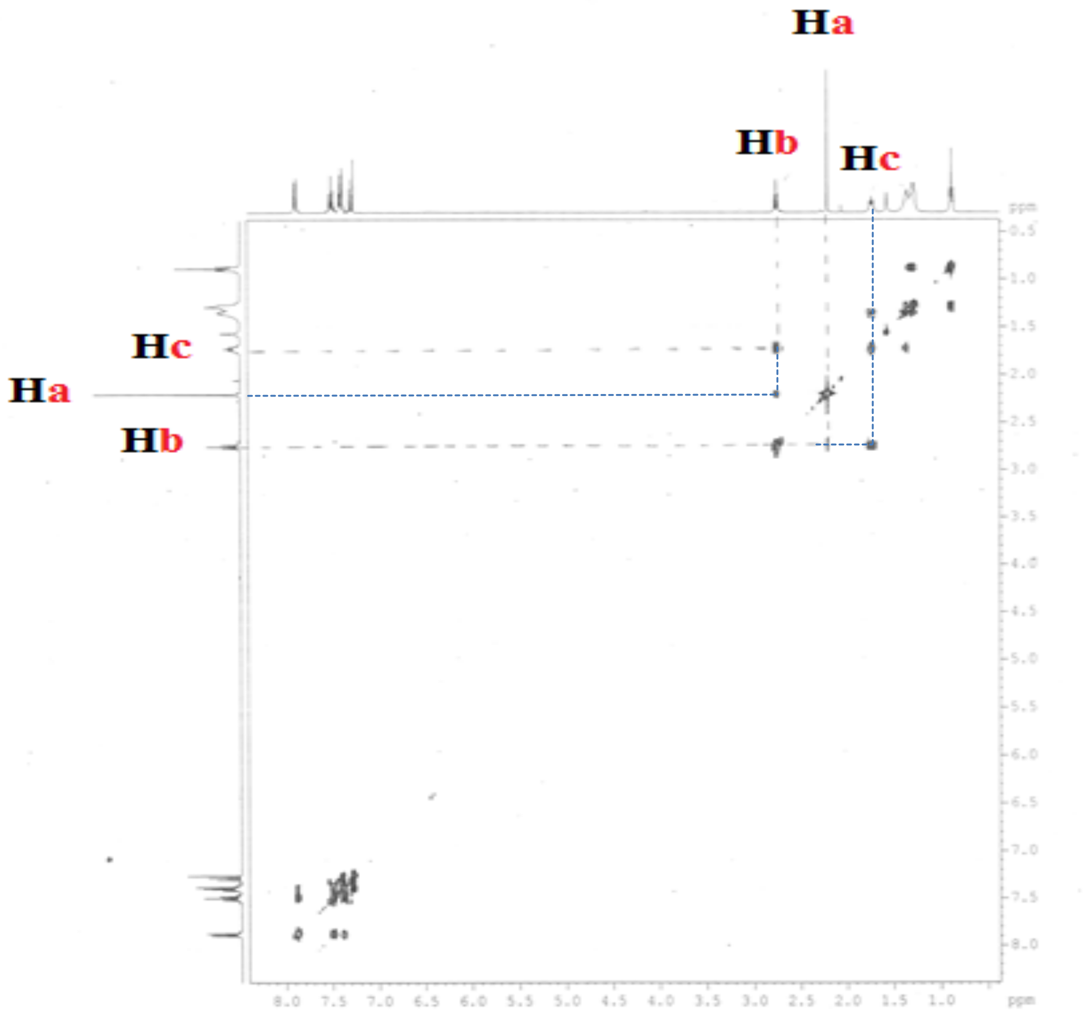

DM-SA4-BF-159-E1-1-cosy

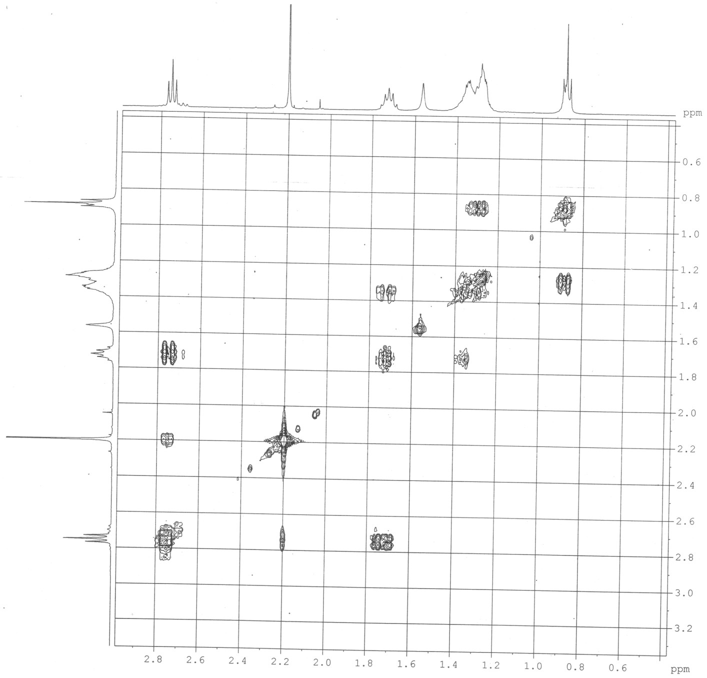

DM-SA4-BF-159-E1-1-cosy

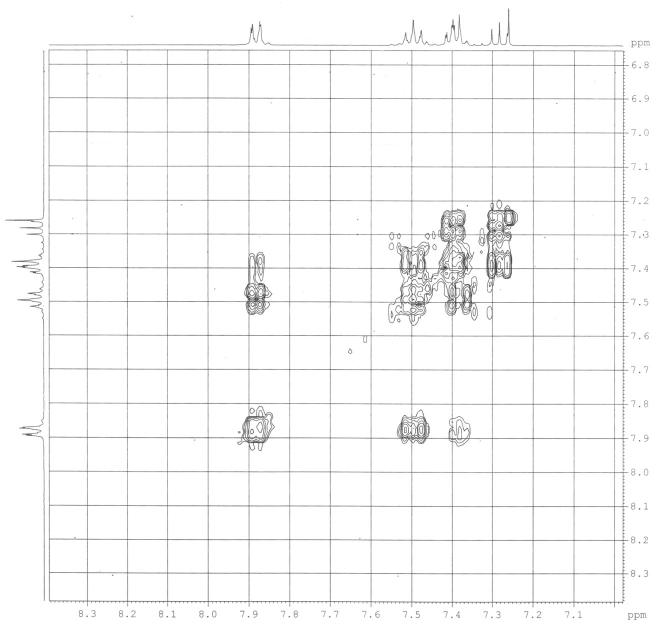

2D NMR: NOESY

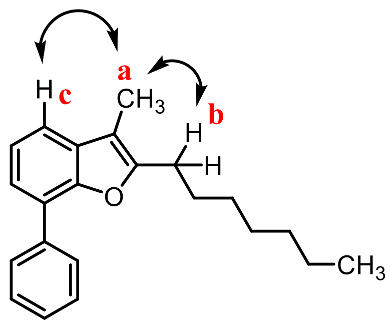
H c
a
CH3
b
H
H
O
CH3

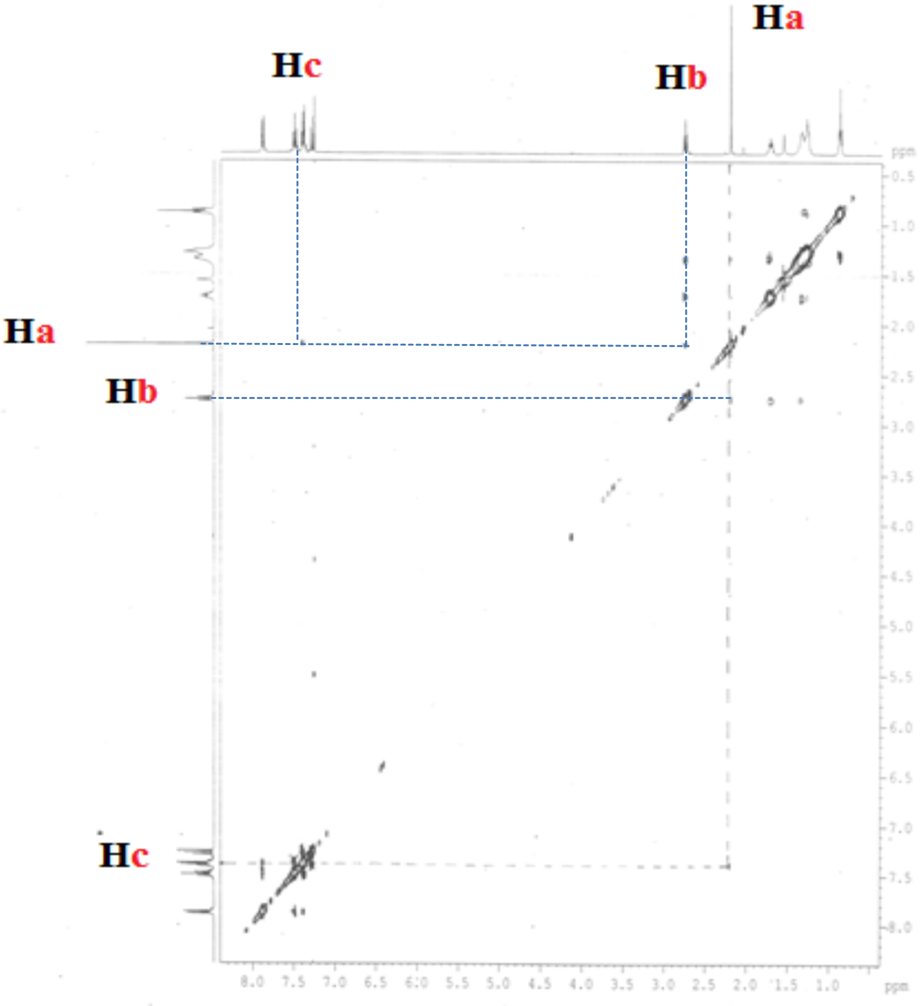
Ha
Hc
Hb
Ha
Hb
Hc
ppm
8.0 7.5 7.0 6.5 6.0 5.5 5.0 4.5 4.0 3.5 3.0 2.5 2.0 1.5 1.0

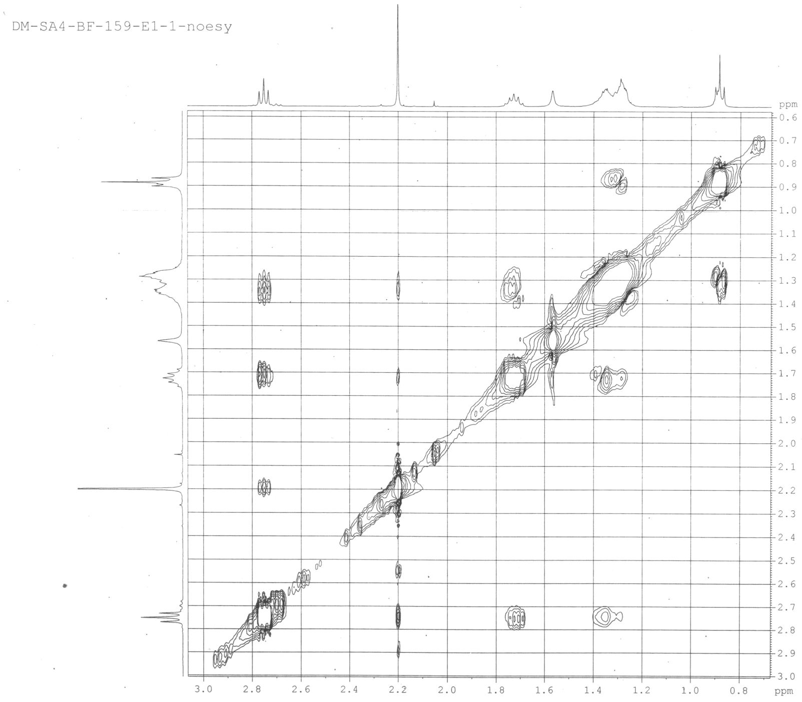
DM-SA4-BF-159-E1-1-noesy
ppm
0.6
0.7
0.8
0.9
1.0
1.1
1.2
1.3
1.4
1.5
1.6
1.7
1.8
1.9
2.0
2.1
2.2
2.3
2.4
2.5
2.6
2.7
2.8
2.9
3.0
3.0 2.8 2.6 2.4 2.2 2.0 1.8 1.6 1.4 1.2 1.0 0.8
ppm

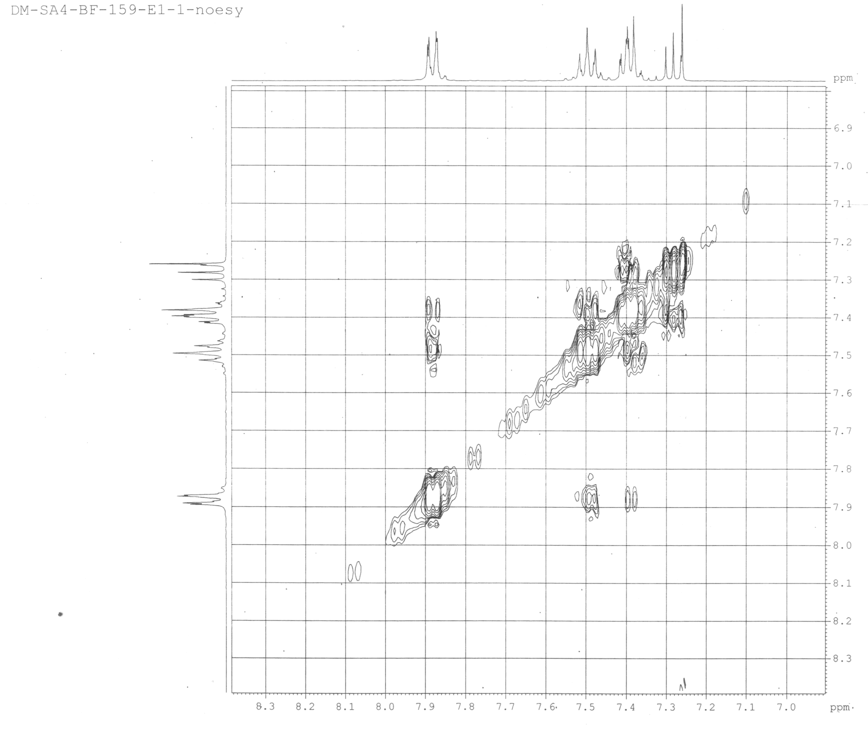
DM-SA4-BF-159-E1-1-noesy
ppm
6.9
7.0
7.1
7.2
7.3
7.4
7.5
7.6
7.7
7.8
7.9
8.0
8.1
8.2
8.3
8.3 8.2 8.1 8.0 7.9 7.8 7.7 7.6 7.5 7.4 7.3 7.2 7.1 7.0
ppm

2D NMR: HMBC

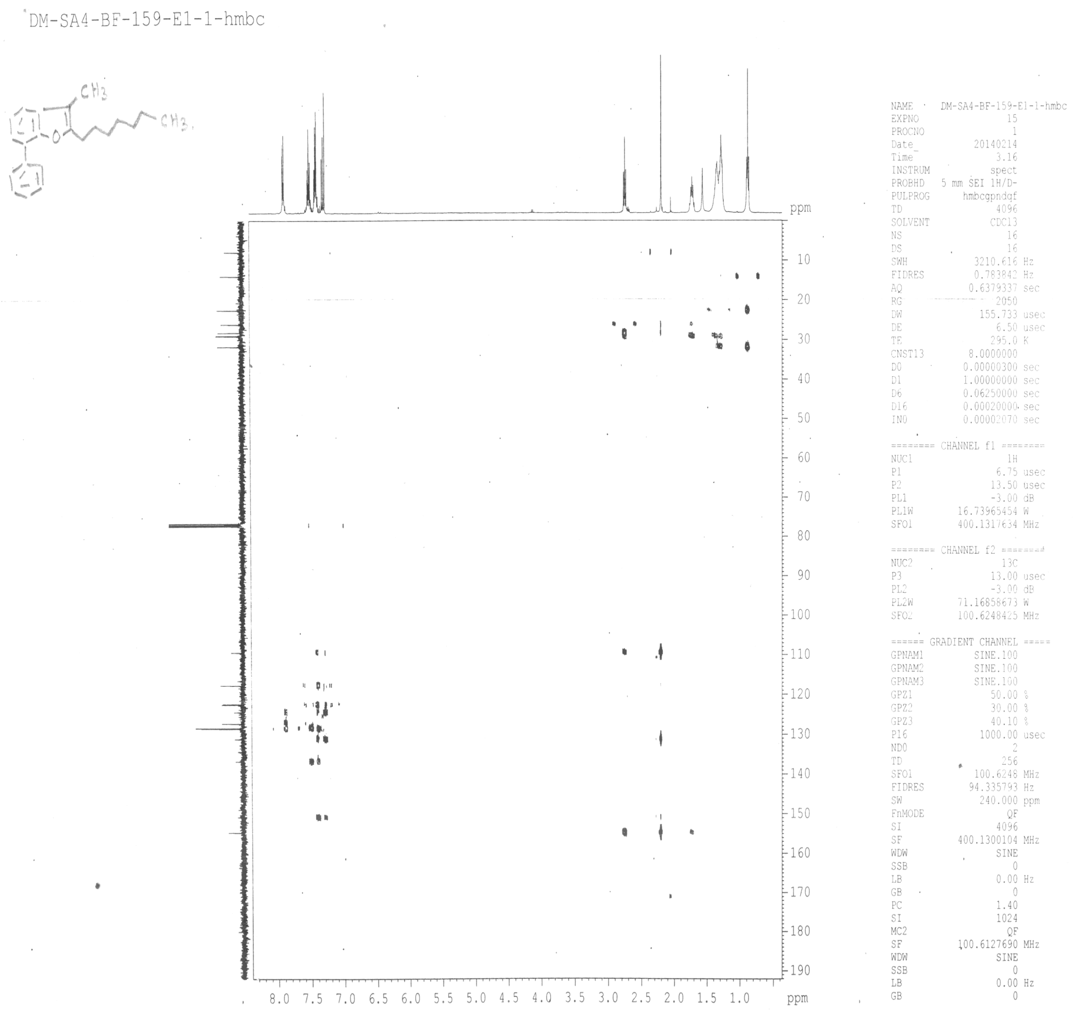
DM-SA4-BF-159-E1-1-hmbc
CH3
CH3
ppm
10
20
30
40
50
60
70
80
90
100
110
120
130
140
150
160
170
180
190
8.0 7.5 7.0 6.5 6.0 5.5 5.0 4.5 4.0 3.5 3.0 2.5 2.0 1.5 1.0 ppm
NAME DM-SA4-BF-159-E1-1-hmbc
EXPNO 15
PROCNO 1
Date_ 20140214
Time 3.16
INSTRUM spect
PROBHD 5 mm SEI 1H/D-
PULPROG hmbcgpndqf
TD 4096
SOLVENT CDCl3
NS 16
DS 16
SWH 3210.616 Hz
FIDRES 0.783842 Hz
AQ 0.6379337 sec
RG 2050
DW 155.733 usec
DE 6.50 usec
TE 295.0 K
CNST13 8.0000000
D0 0.00000300 sec
D1 1.00000000 sec
D6 0.06250000 sec
D16 0.00020000 sec
IN0 0.00002070 sec
======== CHANNEL f1 ========
NUC1 1H
P1 6.75 usec
P2 13.50 usec
PL1 -3.00 dB
PL1W 16.73965454 W
SFO1 400.1317634 MHz
======== CHANNEL f2 ========
NUC2 13C
P3 13.00 usec
PL2 -3.00 dB
PL2W 71.16858673 W
SFO2 100.6248425 MHz
====== GRADIENT CHANNEL =====
GPNAM1 SINE.100
GPNAM2 SINE.100
GPNAM3 SINE.100
GPZ1 50.00 %
GPZ2 30.00 %
GPZ3 40.10 %
P16 1000.00 usec
ND0 2
TD 256
SFO1 100.6248 MHz
FIDRES 94.335793 Hz
SW 240.000 ppm
FnMODE QF
SI 4096
SF 400.1300104 MHz
WDW SINE
SSB 0
LB 0.00 Hz
GB 0
PC 1.40
SI 1024
MC2 QF
SF 100.6127690 MHz
WDW SINE
SSB 0
LB 0.00 Hz
GB 0

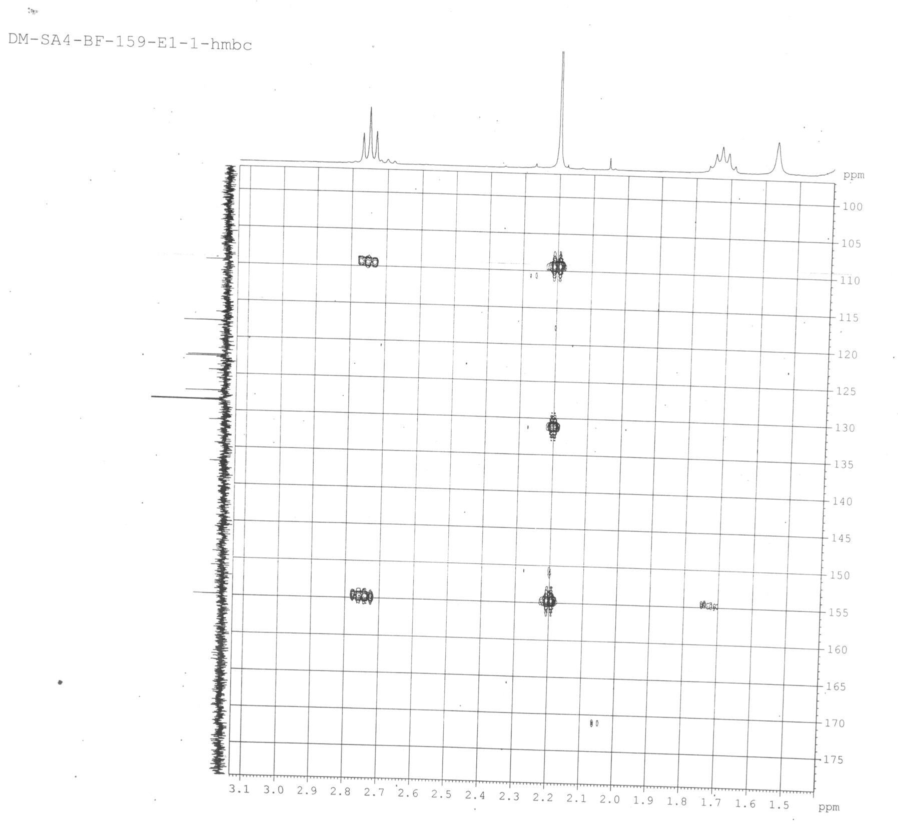

DM-SA4-BF-159-E1-1-hmbc
ppm
100
105
110
115
120
125
130
135
140
145
150
155
160
165
170
175
3.1 3.0 2.9 2.8 2.7 2.6 2.5 2.4 2.3 2.2 2.1 2.0 1.9 1.8 1.7 1.6 1.5
ppm

2D NMR: HSQC

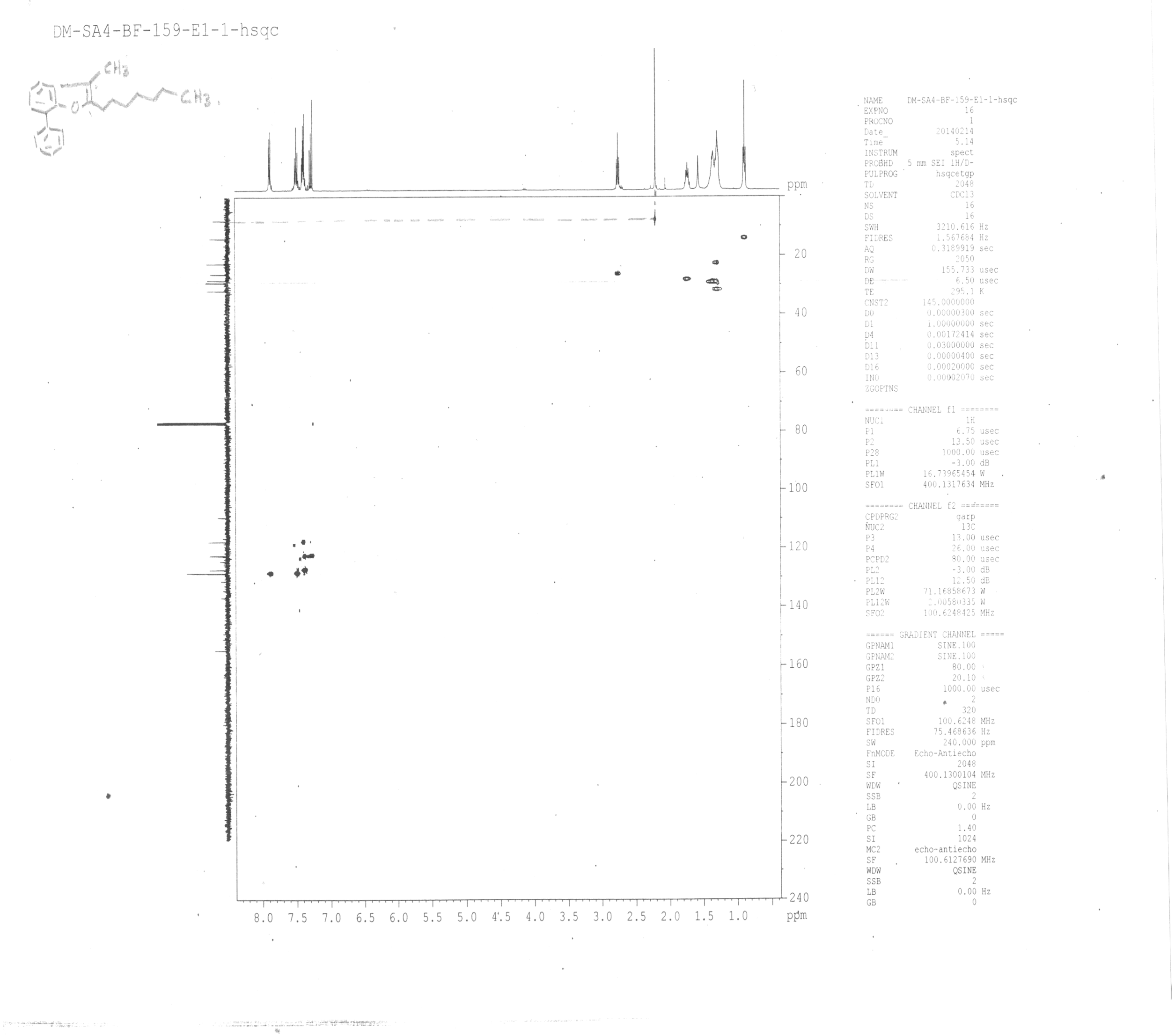

DM-SA4-BF-159-E1-1-hsqc

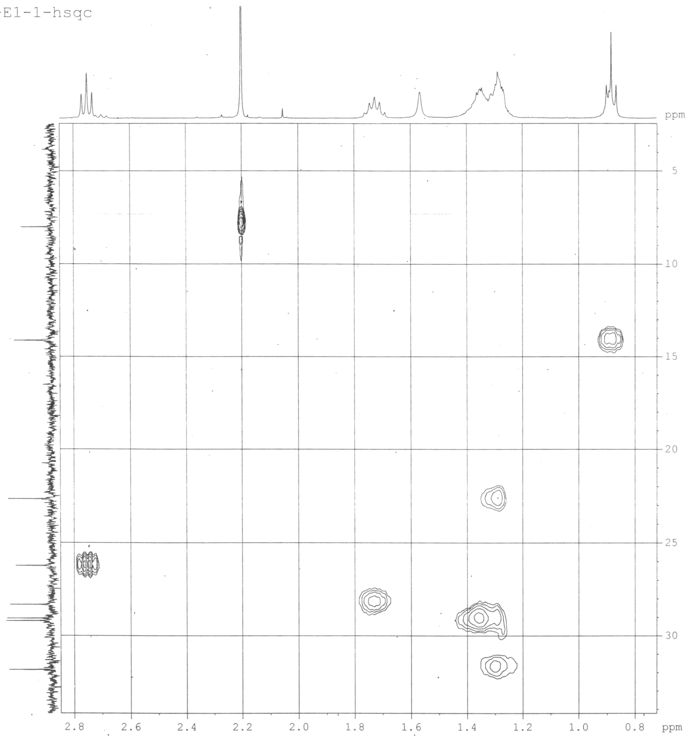

DM-SA4-BF-159-E1-1-hsqc

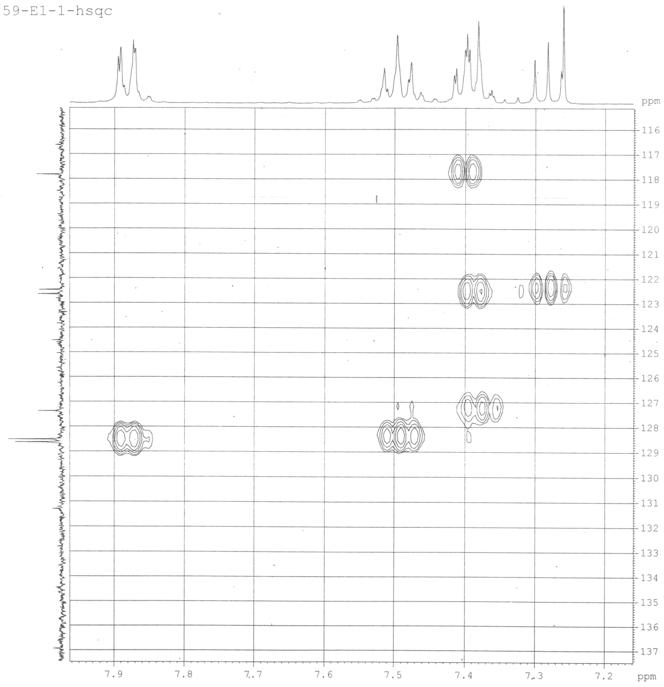

Table 2, Entry 6a:

^{1}H NMR (500MHz, CDCl3) : (*E*)-methyl 3-ethylidene-2-phenyl-2,3-dihydrobenzofuran-5-carboxylate

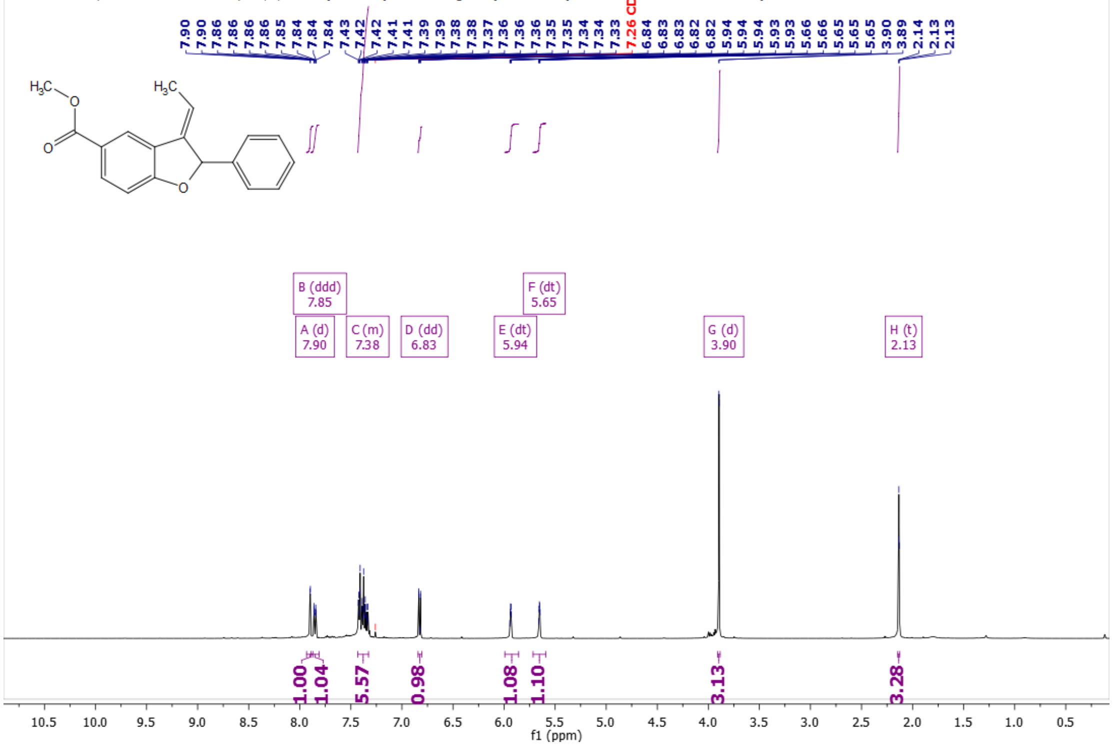

^{13}C NMR (126MHz, CDCl3) : (*E*)-methyl 3-ethylidene-2-phenyl-2,3-dihydrobenzofuran-5-carboxylate

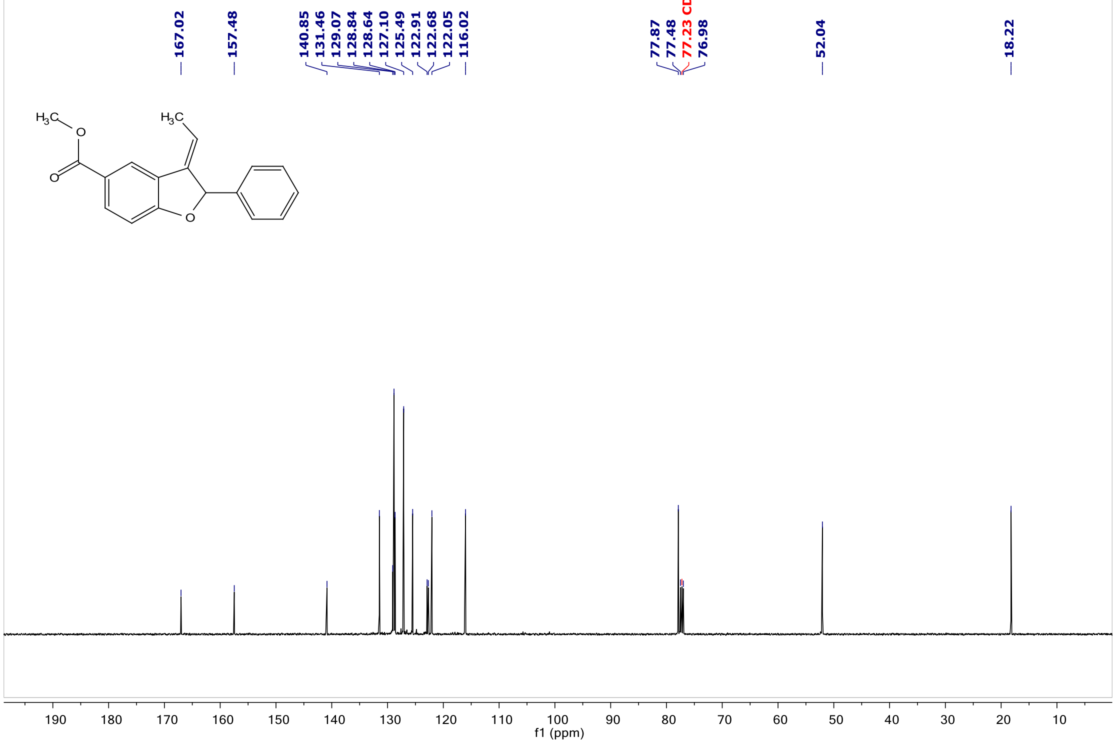

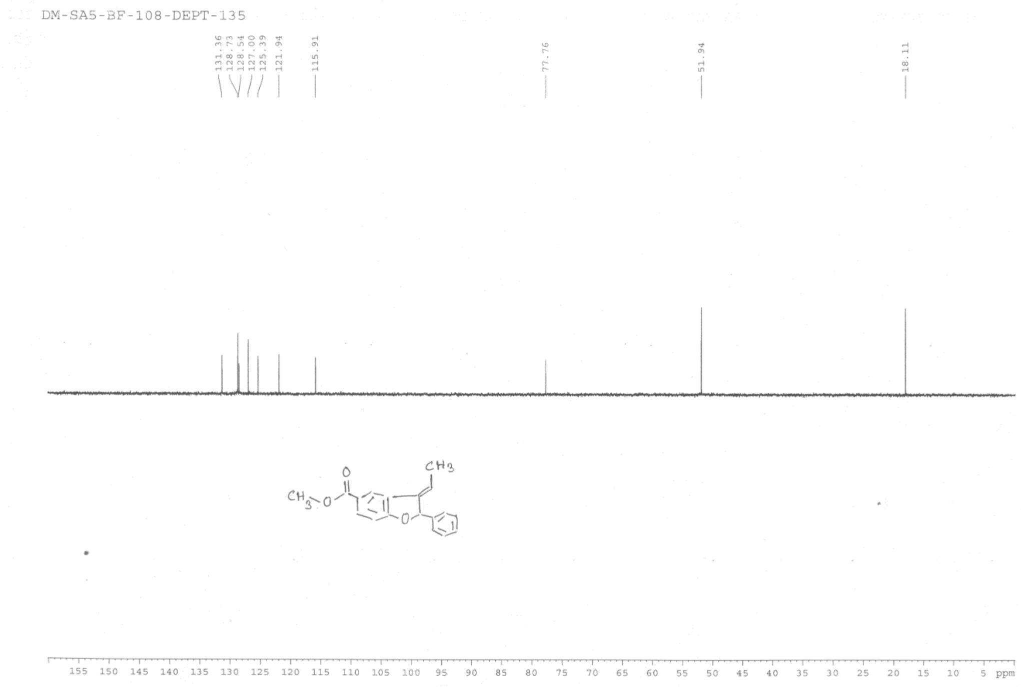
DM-SA5-BF-108-DEPT-135
131.36
128.73
128.54
127.00
125.39
121.94
115.91
77.76
51.94
18.11
155 150 145 140 135 130 125 120 115 110 105 100 95 90 85 80 75 70 65 60 55 50 45 40 35 30 25 20 15 10 5 ppm

1D NMR:NOE

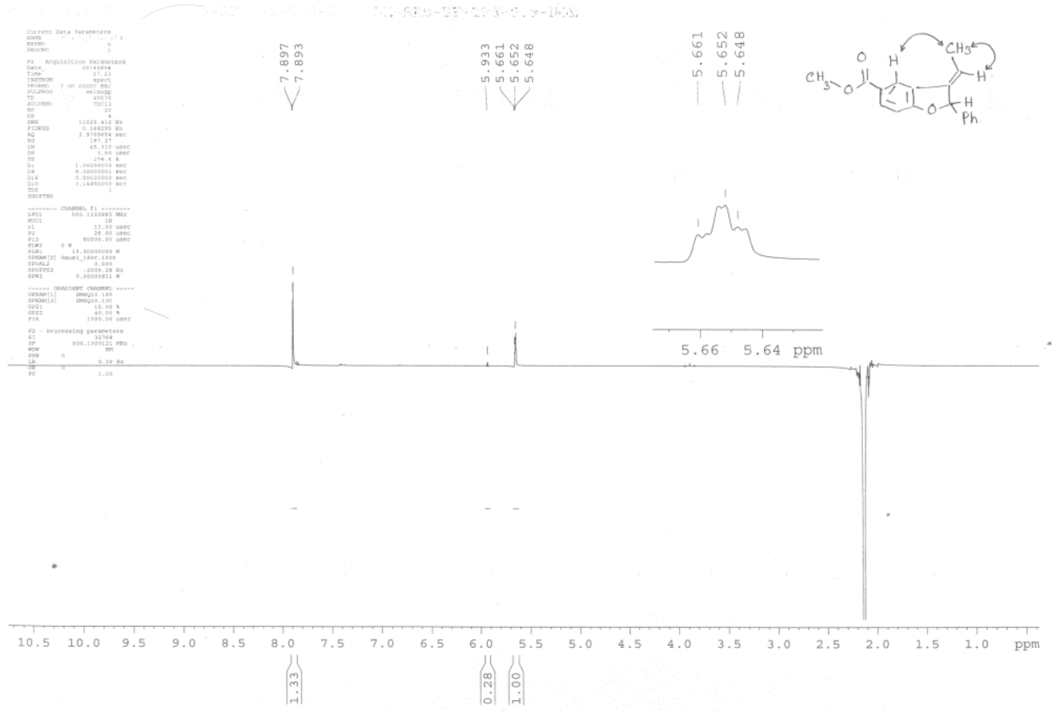

7.897
7.893
5.933
5.661
5.652
5.648
5.661
5.652
5.648
5.66 5.64 ppm
10.5 10.0 9.5 9.0 8.5 8.0 7.5 7.0 6.5 6.0 5.5 5.0 4.5 4.0 3.5 3.0 2.5 2.0 1.5 1.0 ppm
1.33
0.28
1.00

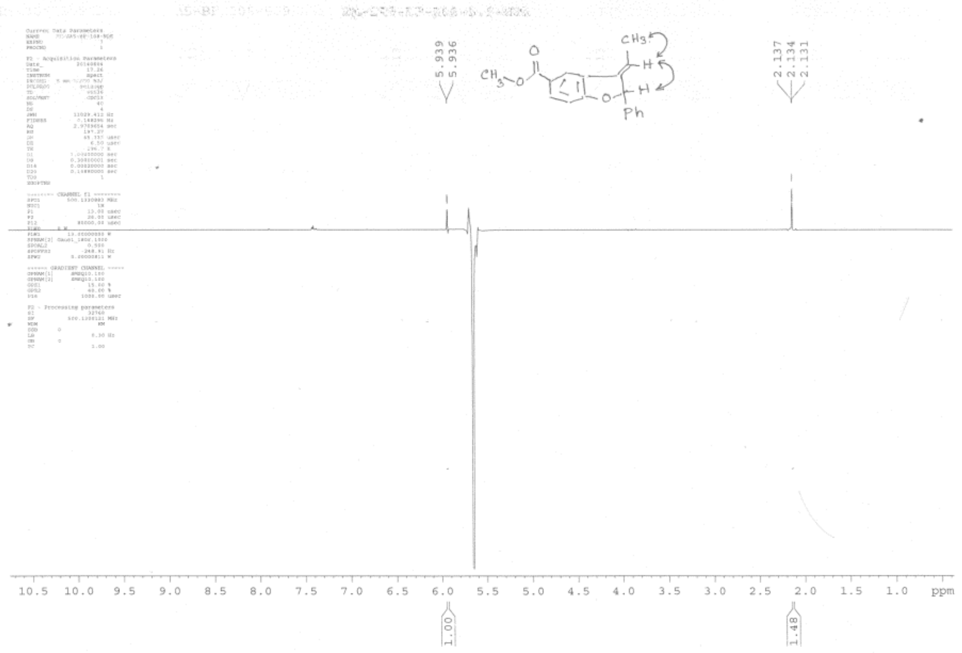

5.939
5.936
2.137
2.134
2.131
10.5 10.0 9.5 9.0 8.5 8.0 7.5 7.0 6.5 6.0 5.5 5.0 4.5 4.0 3.5 3.0 2.5 2.0 1.5 1.0 ppm
1.00
1.48

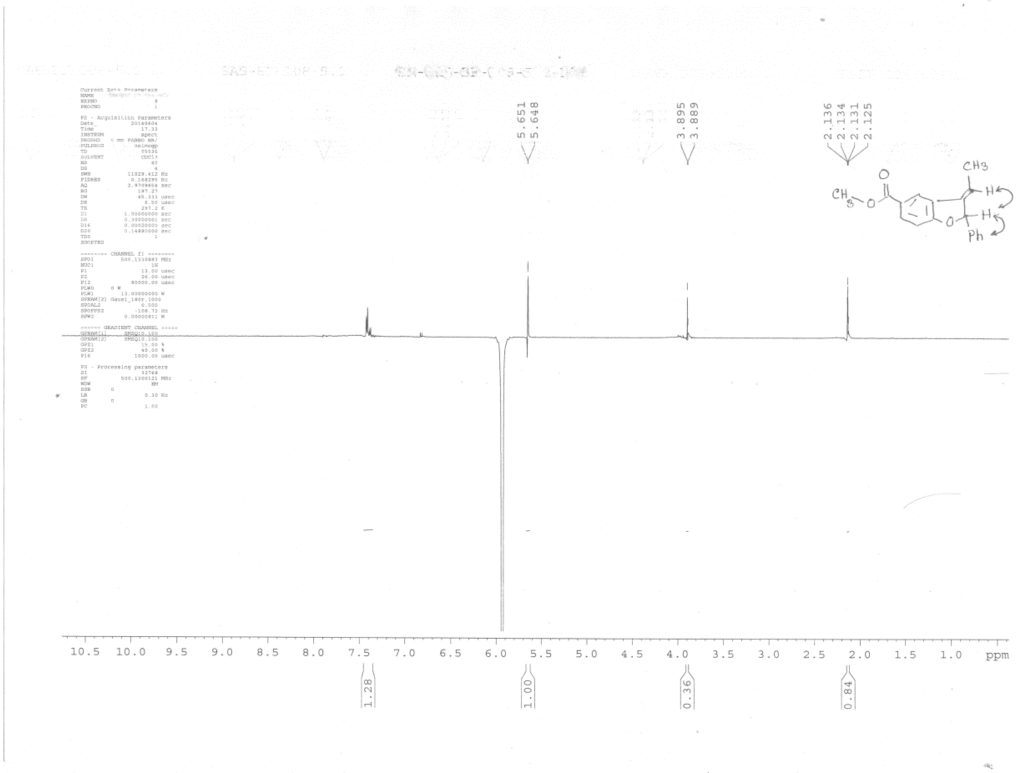

2D NMR: COSY

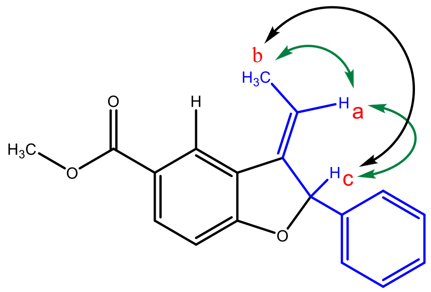

O
H
H3C
O
b
H3C
H a
H c
O

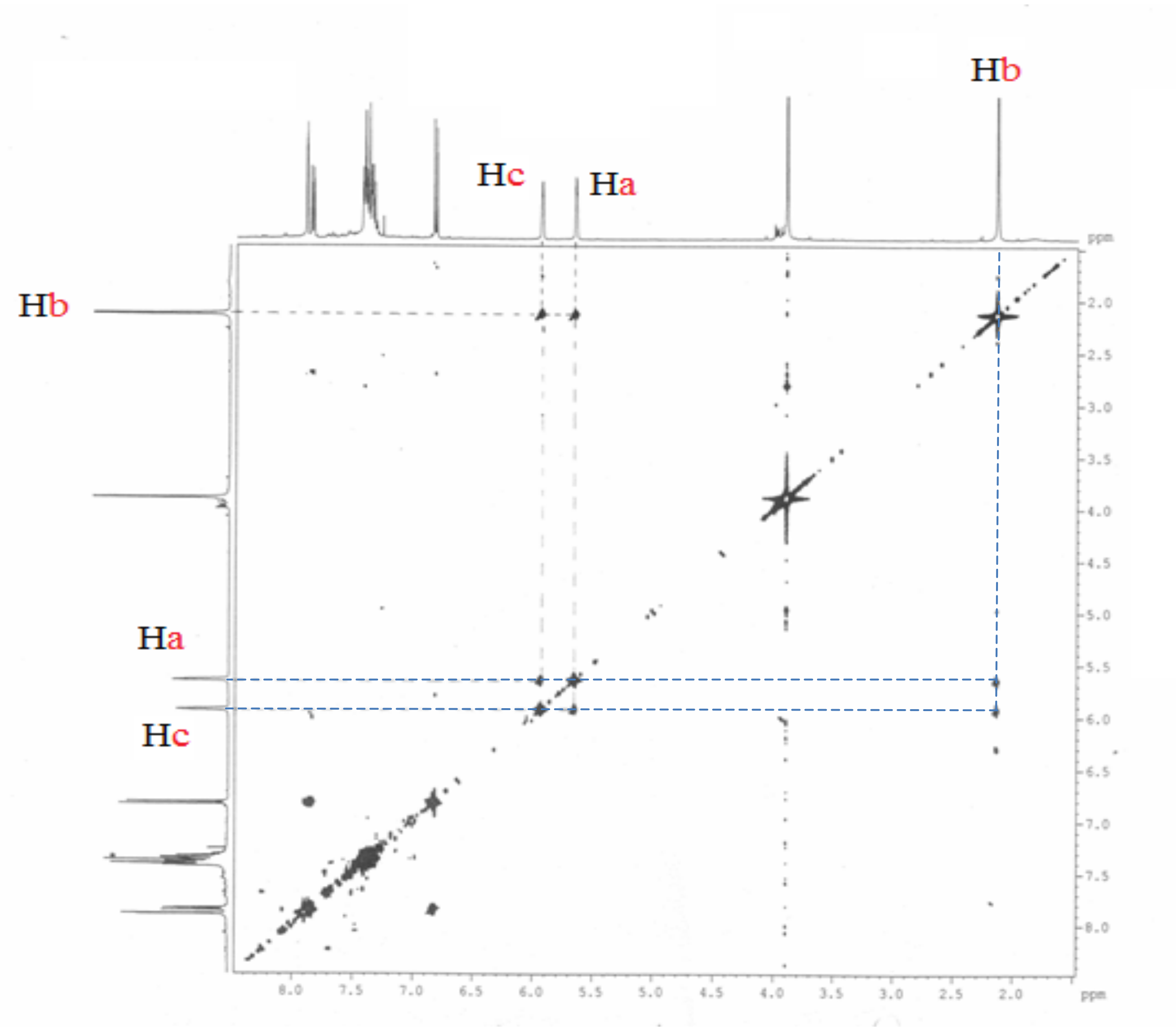

Hb
Hc
Ha
Hb
Ha
Hc
ppm
8.0
7.5
7.0
6.5
6.0
5.5
5.0
4.5
4.0
3.5
3.0
2.5
2.0
ppm

2D NMR: NOESY

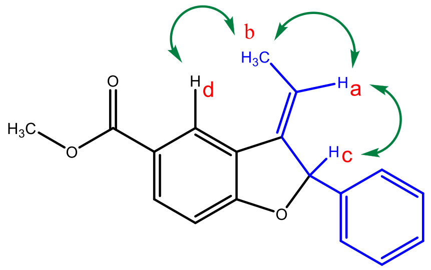

O
H3C
O
H d
b
H3C
H a
H c
O

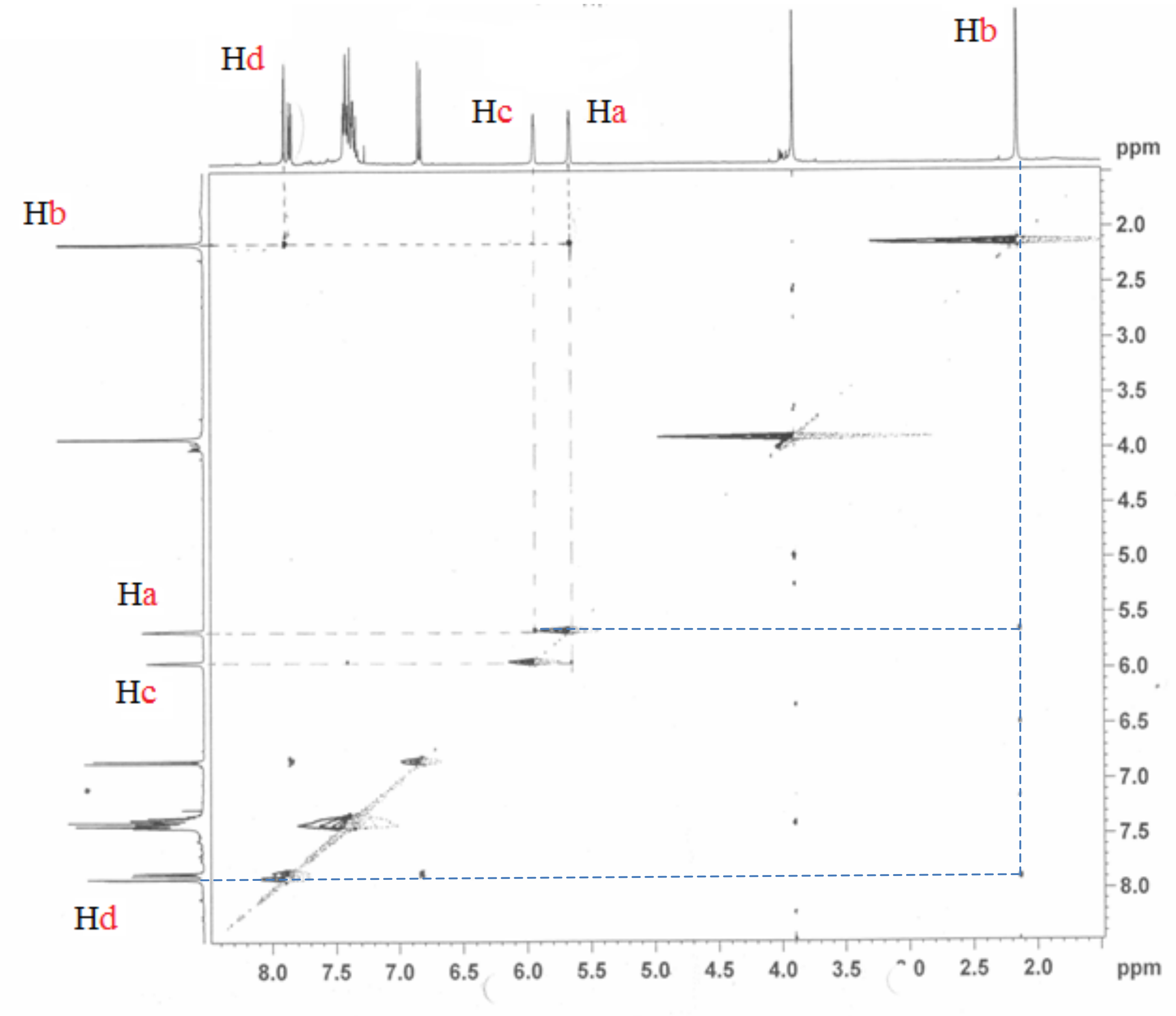

Hd
Hc
Ha
Hb
ppm
Hb
Ha
Hc
Hd
2.0
2.5
3.0
3.5
4.0
4.5
5.0
5.5
6.0
6.5
7.0
7.5
8.0
8.0
7.5
7.0
6.5
6.0
5.5
5.0
4.5
4.0
3.5
2.5
2.0
ppm

Table 2, Entry 6b:

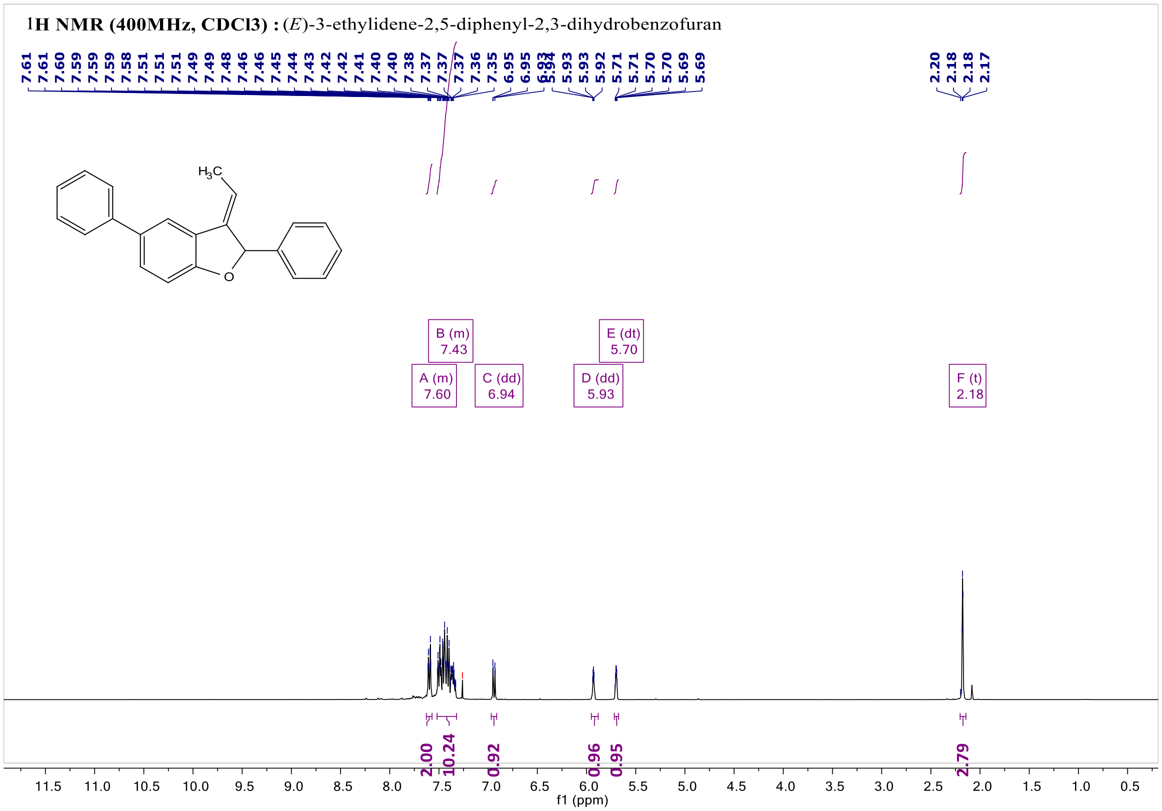

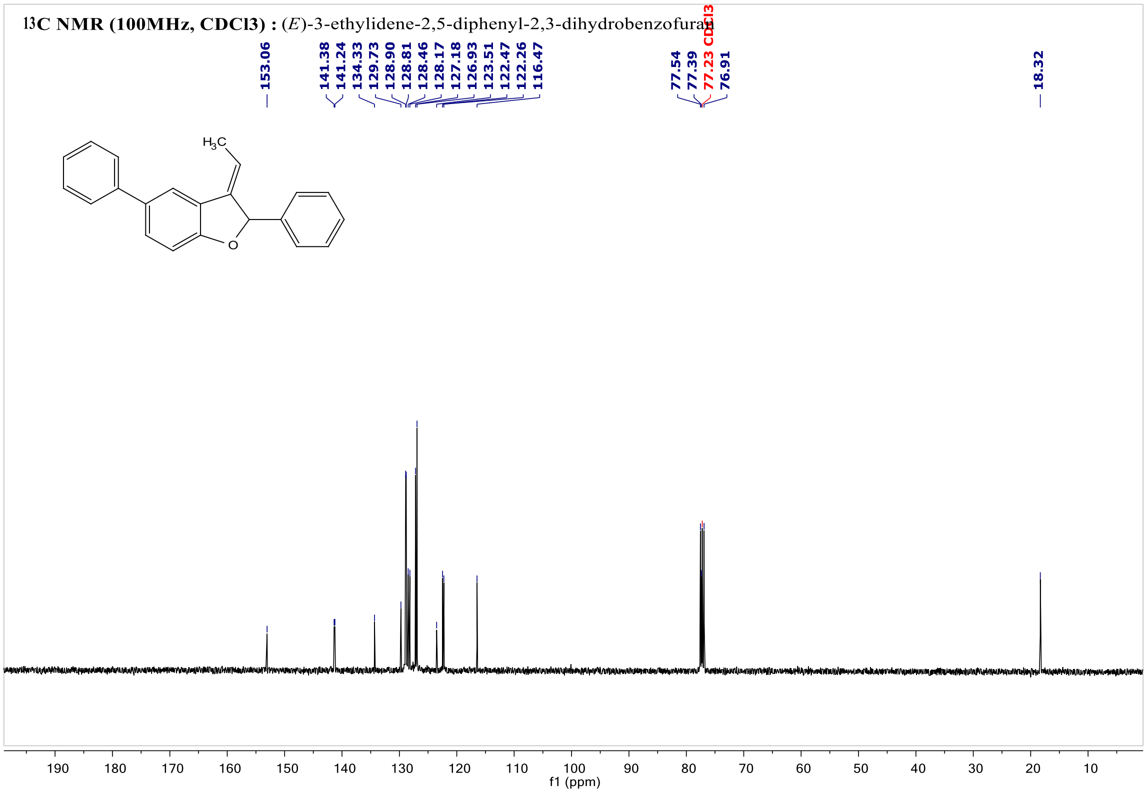

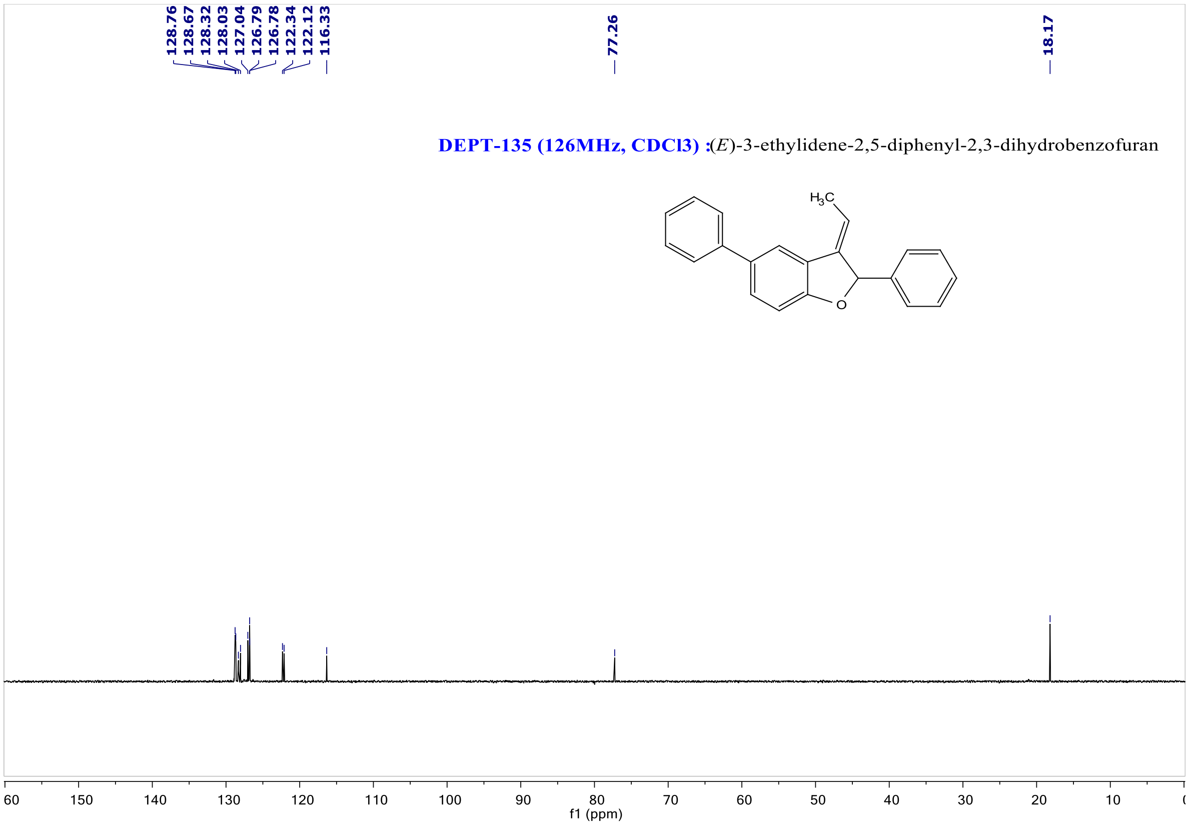

Table 2, Entry 6c:

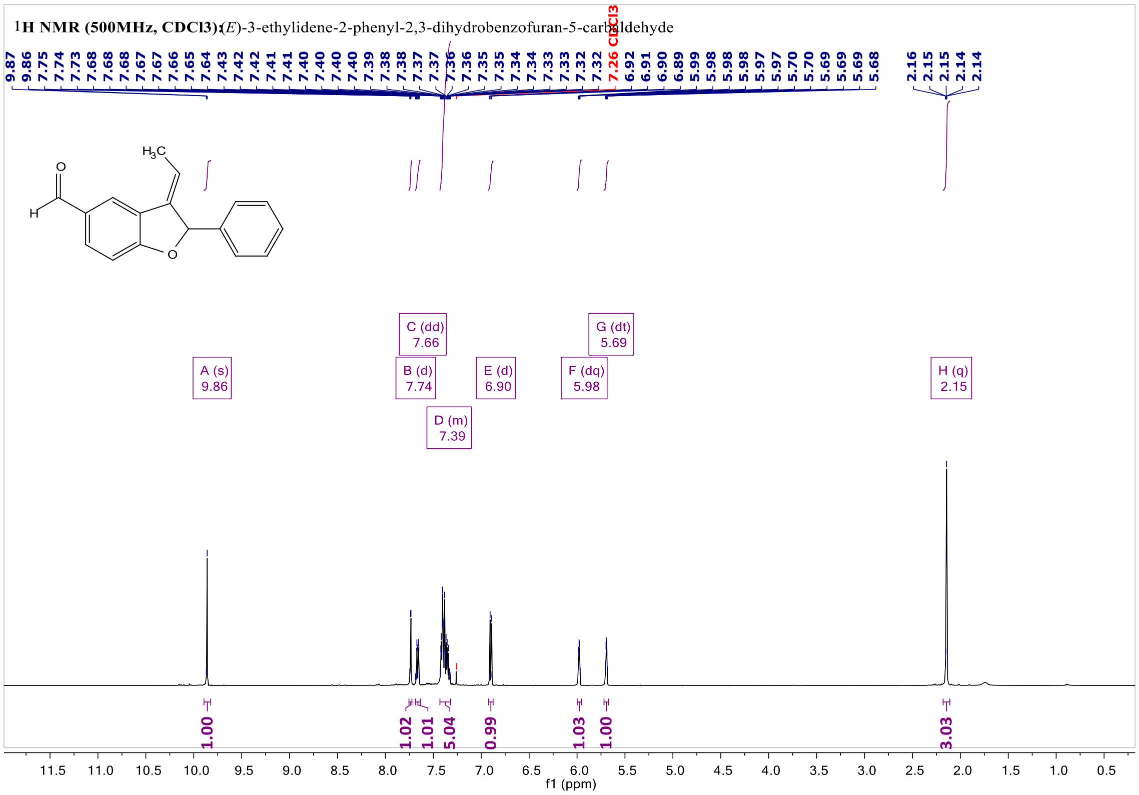

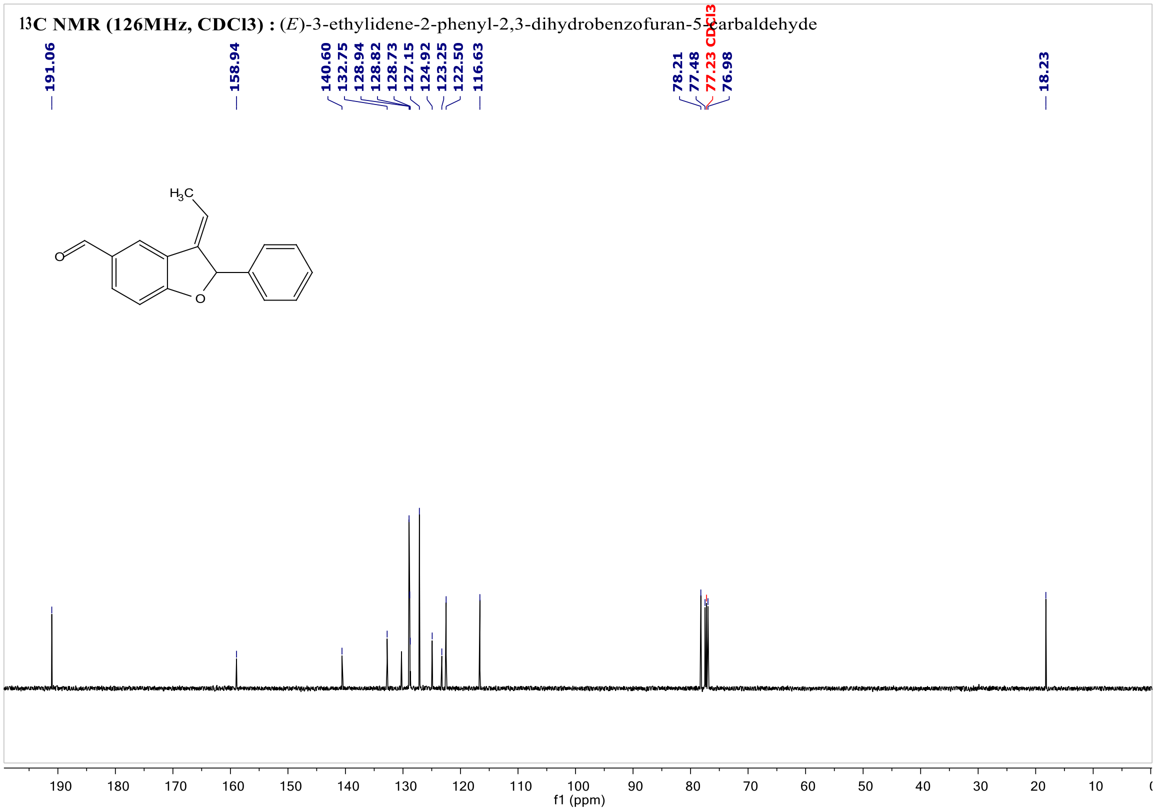

Table 2, Entry 6d:

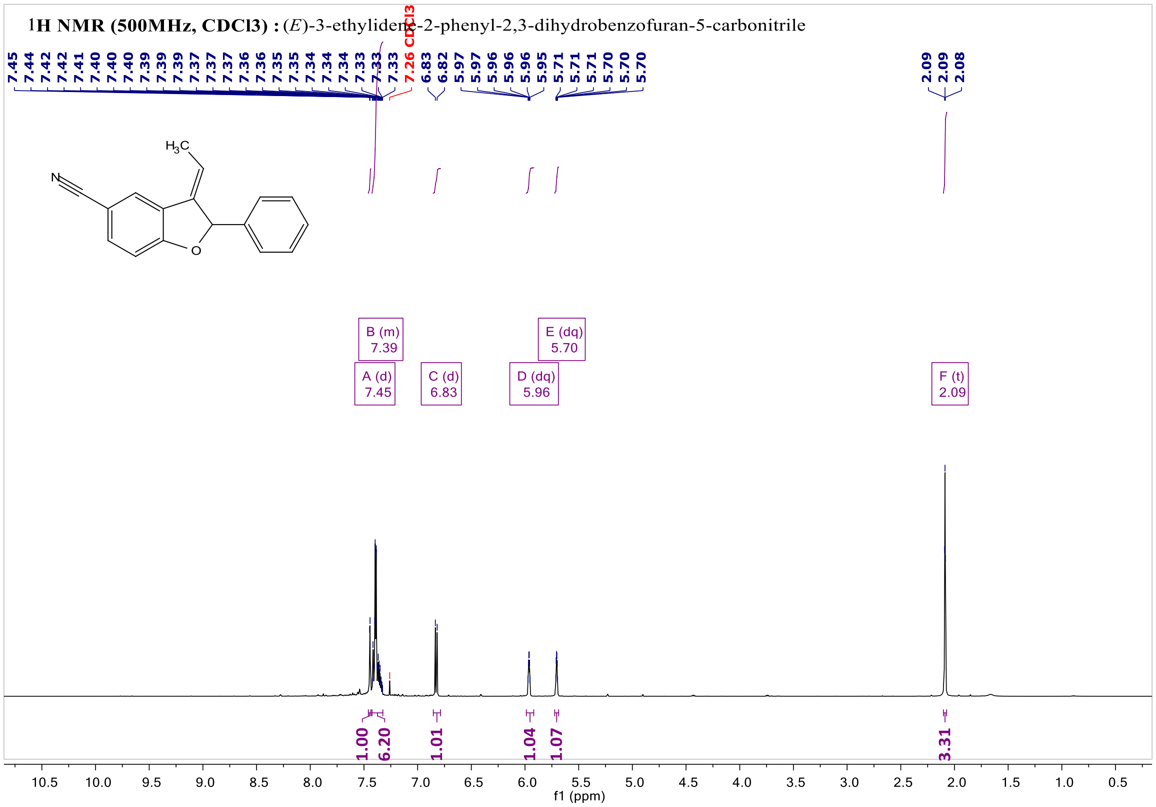

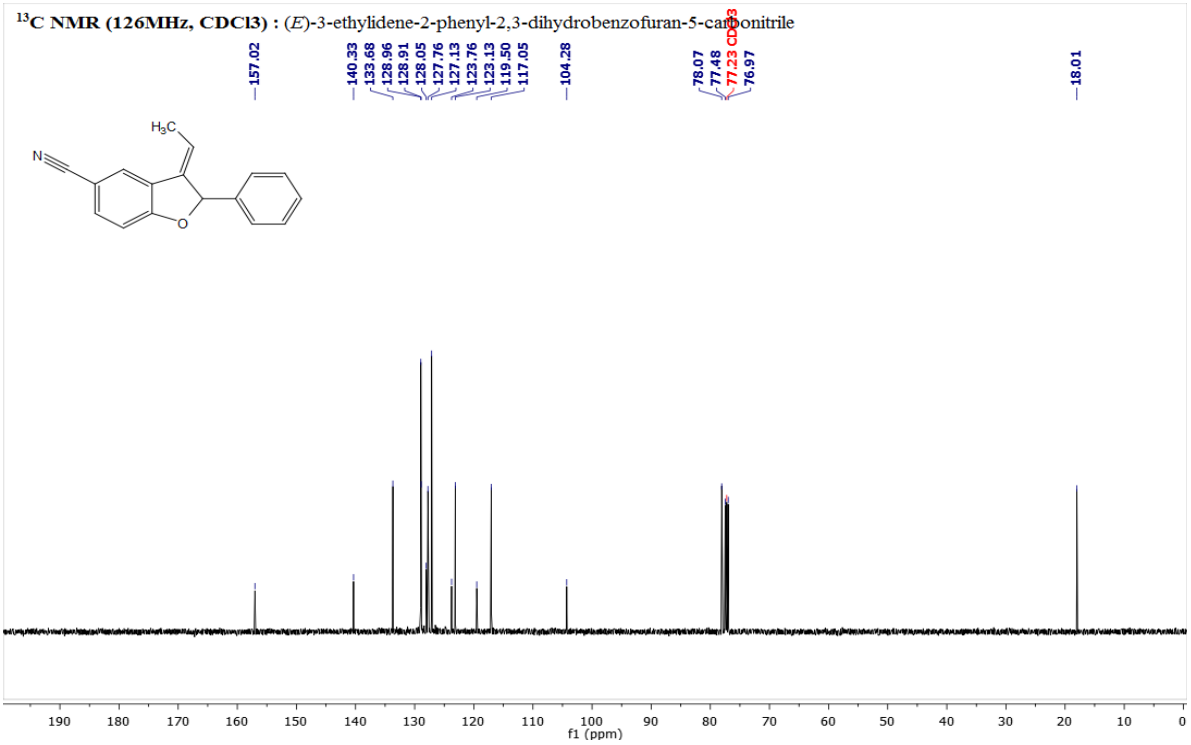
13C NMR (126MHz, CDCl3) : (E)-3-ethylidene-2-phenyl-2,3-dihydrobenzofuran-5-carbonitrile
157.02
140.33
133.68
128.96
128.91
128.05
127.76
127.13
123.76
123.13
119.50
117.05
104.28
78.07
77.48
77.23 CDCl3
76.97
18.01
f1 (ppm)

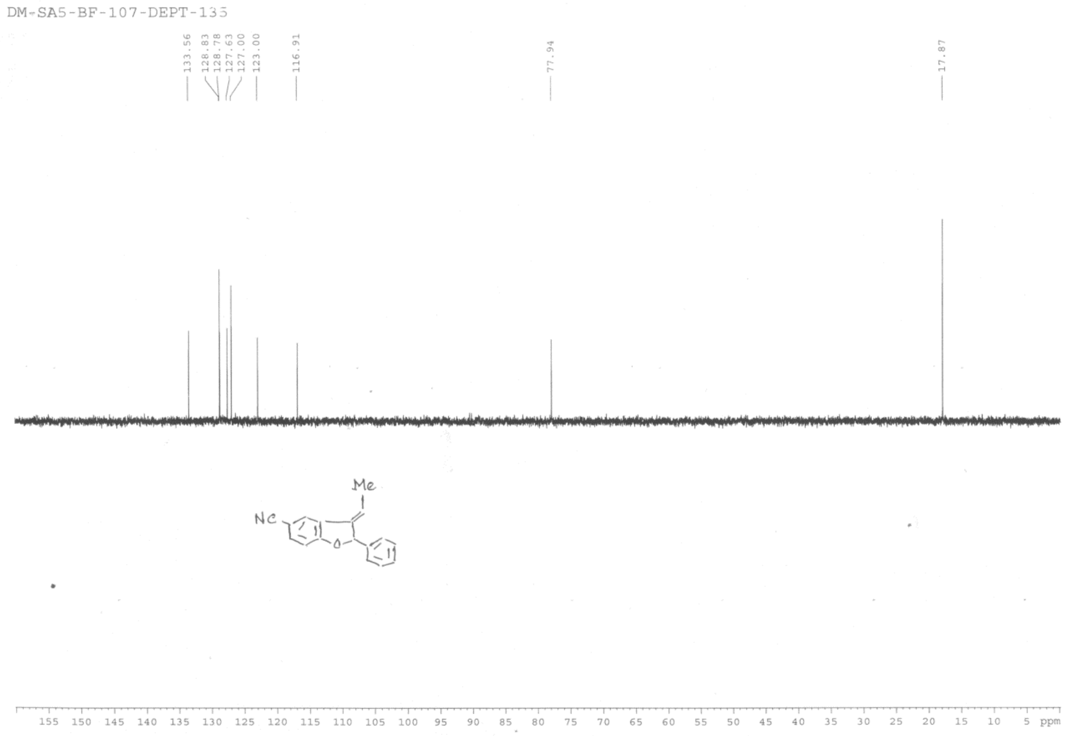
DM-SA5-BF-107-DEPT-135
133.56
128.83
128.78
127.63
127.00
123.00
116.91
77.94
17.87
ppm

Table 2, Entry 6e:

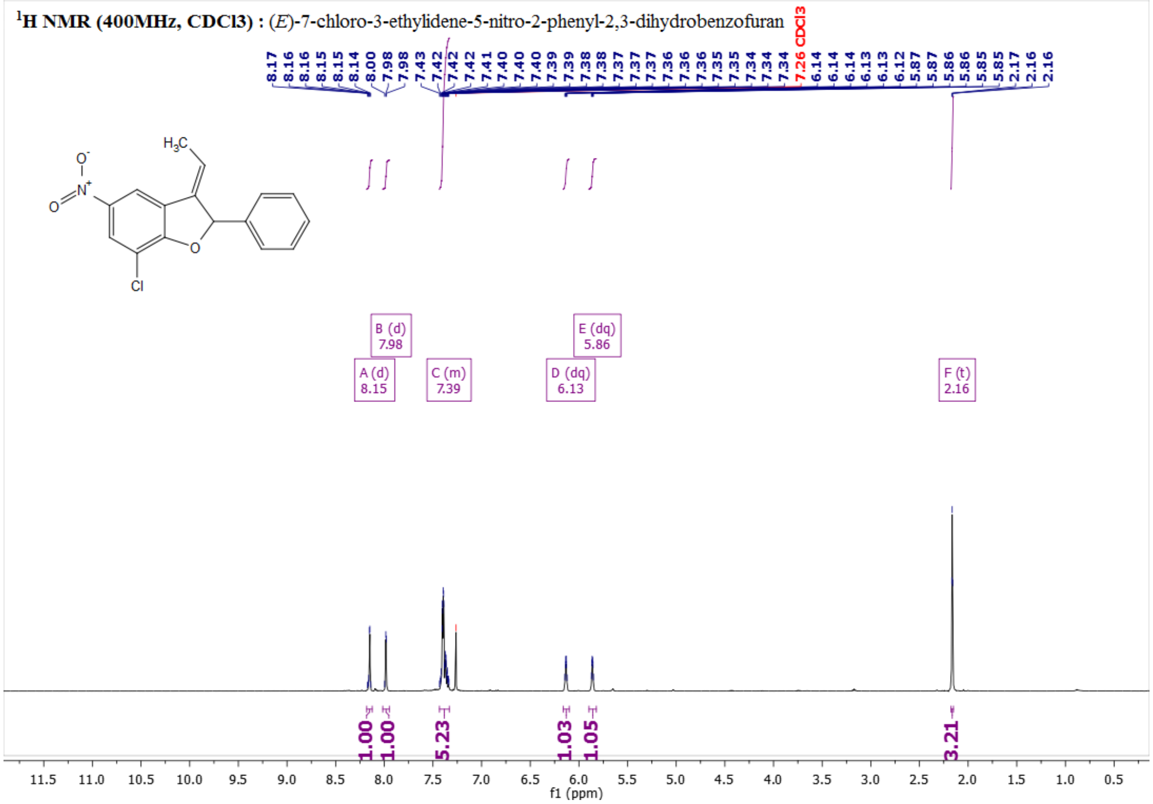

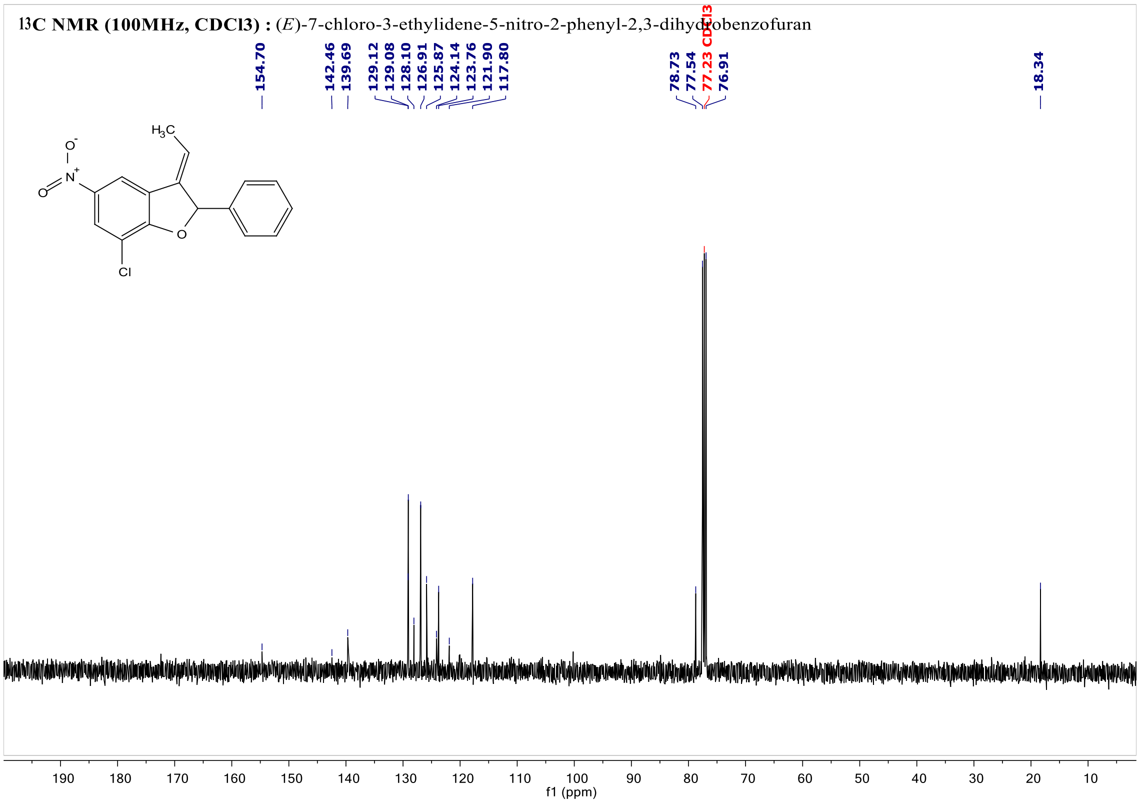

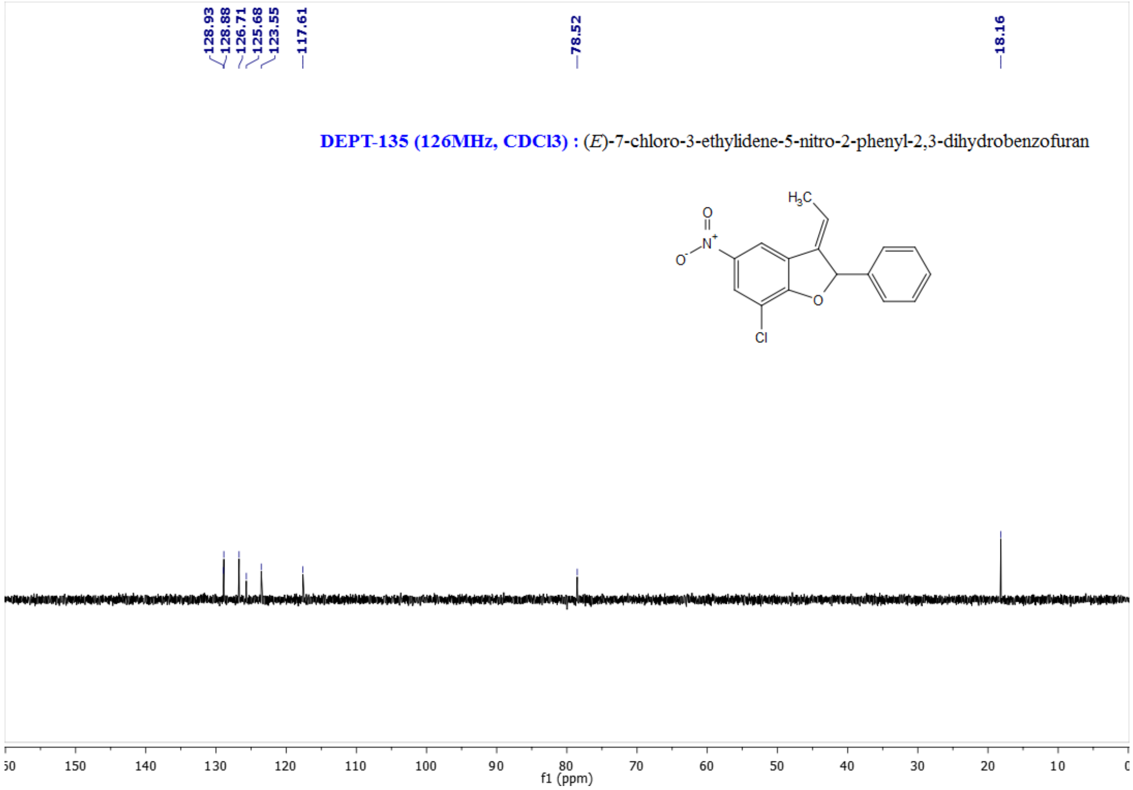

Table 2, Entry 6f:

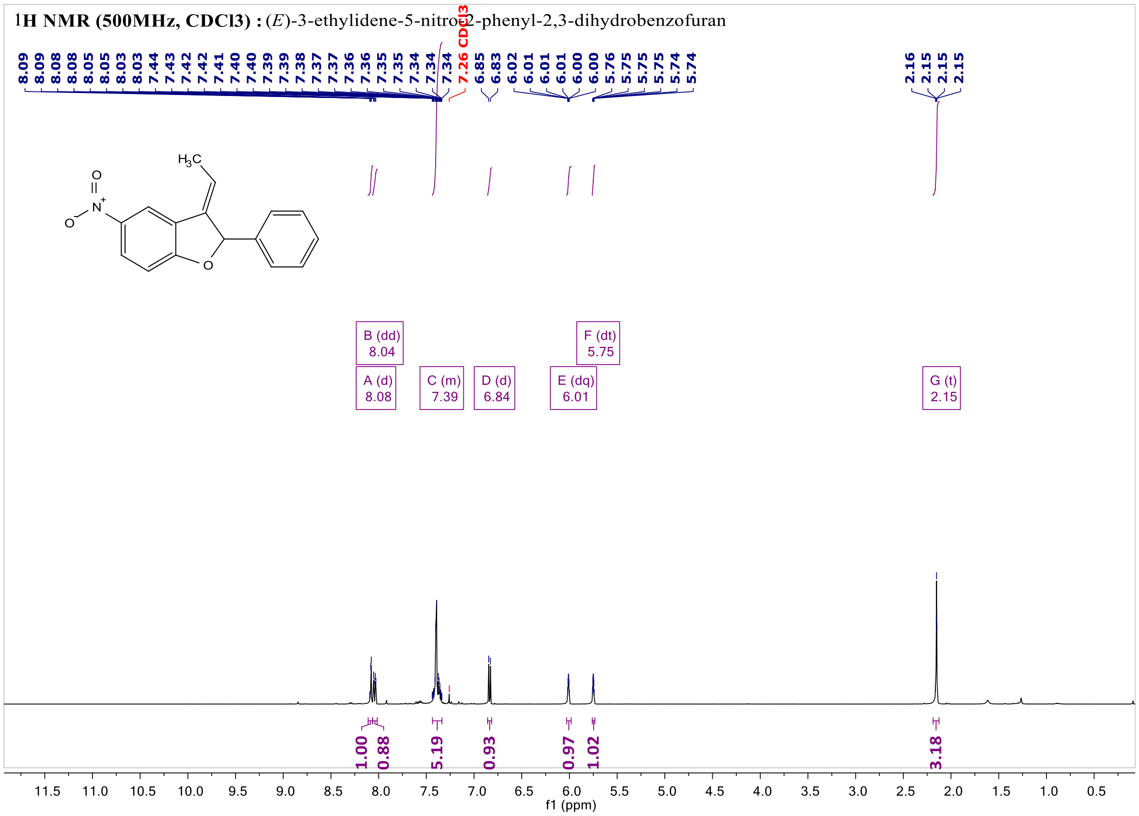

^{13}C NMR (126MHz, CDCl3) : (*E*)-3-ethylidene-5-nitro-2-phenyl-2,3-dihydrobenzofuran

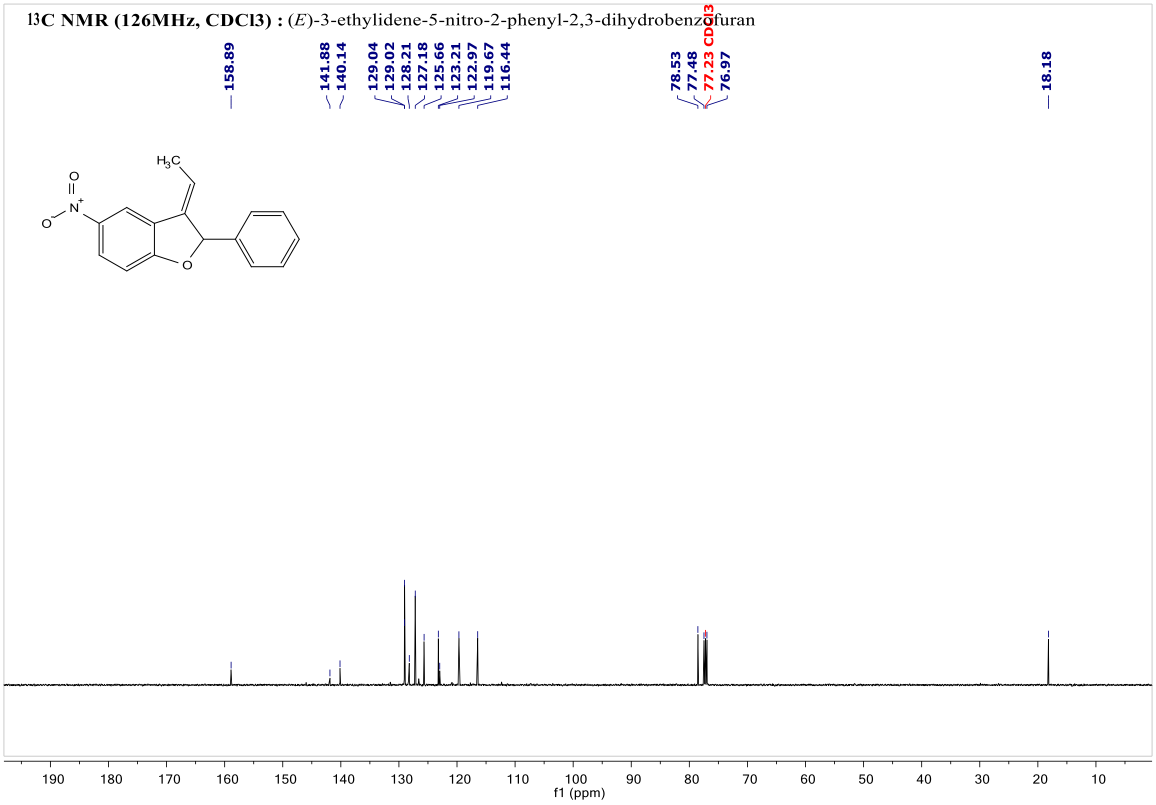

DEPT-135 (126MHz, CDCl3) : (*E*)-3-ethylidene-5-nitro-2-phenyl-2,3-dihydrobenzofuran

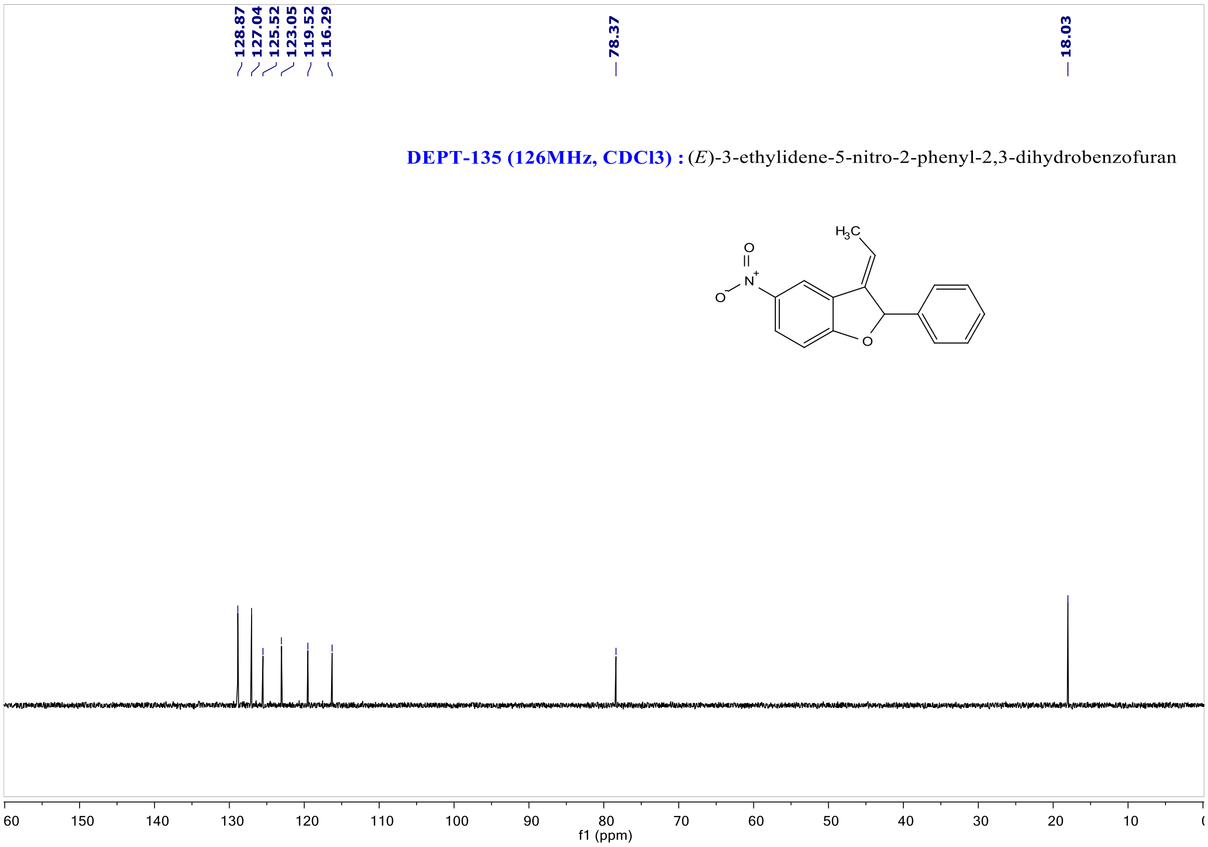

Scheme 4, Entry3'a:

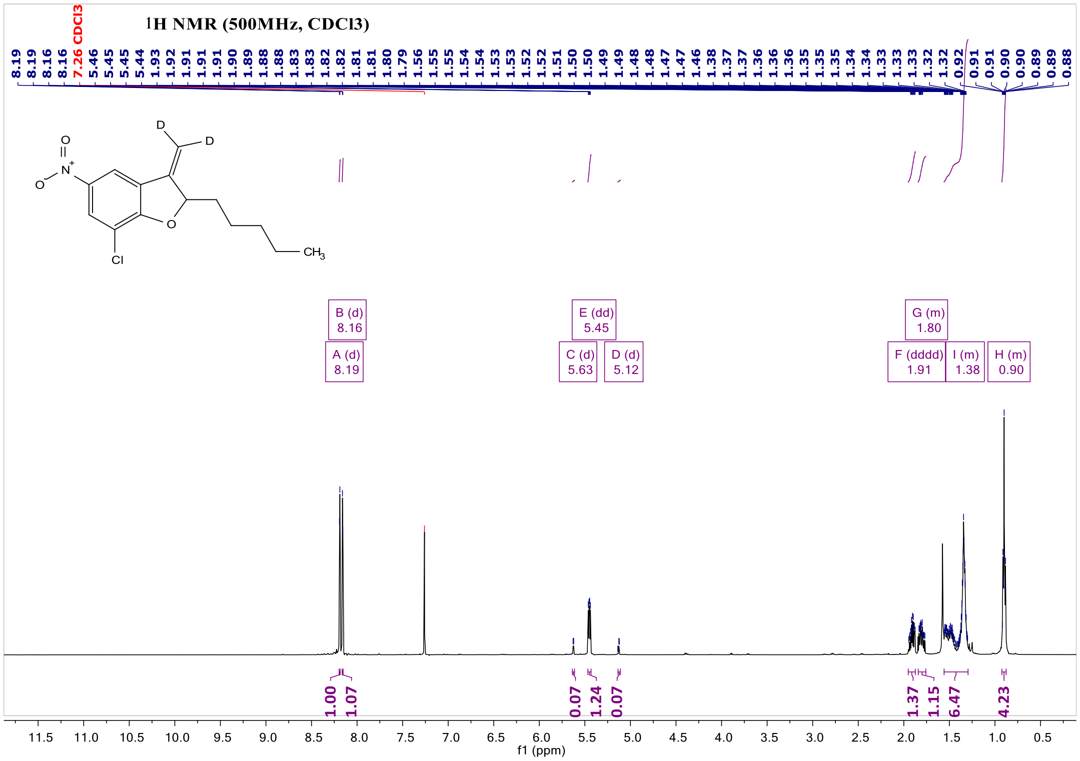

Scheme 4, Entry 3'b:

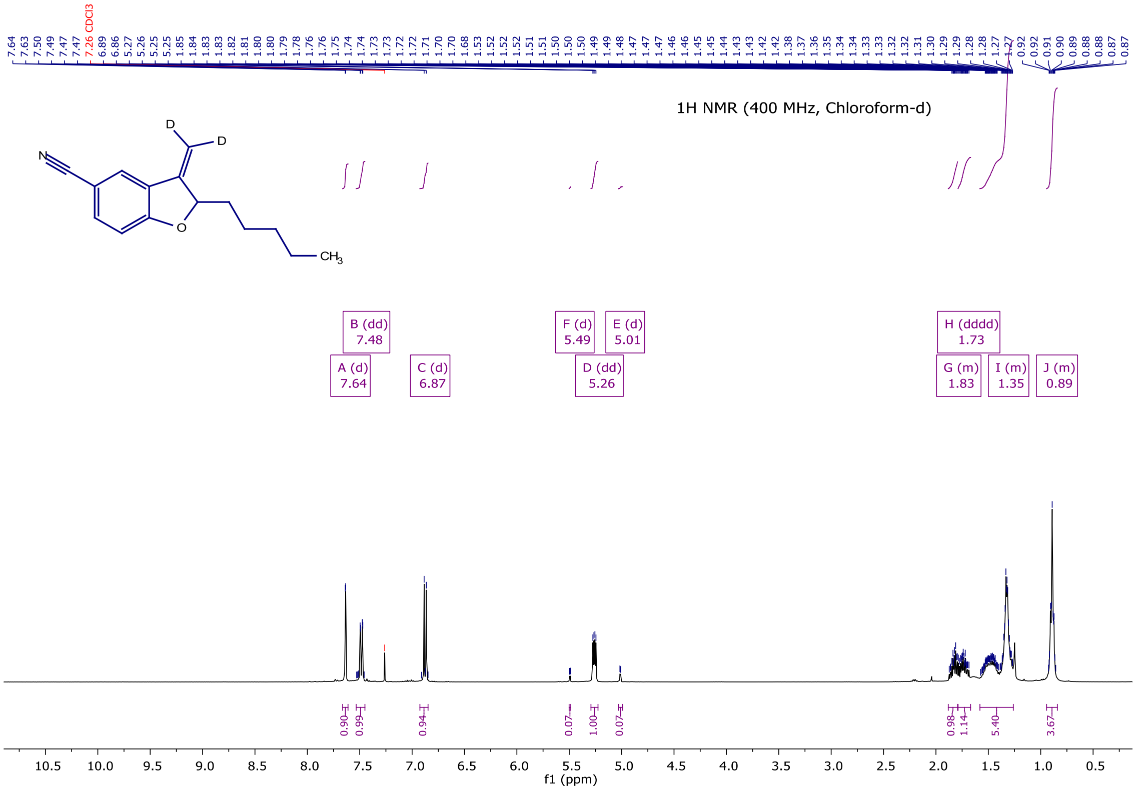

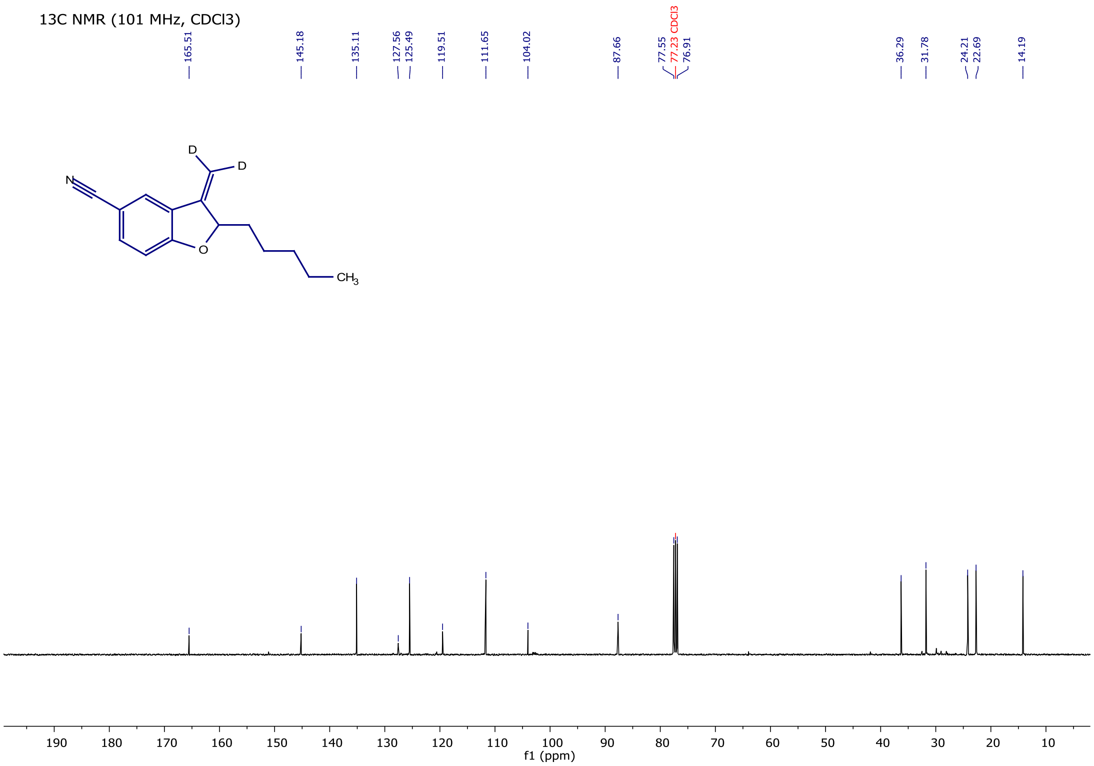

Scheme 4, Entry 3'c:

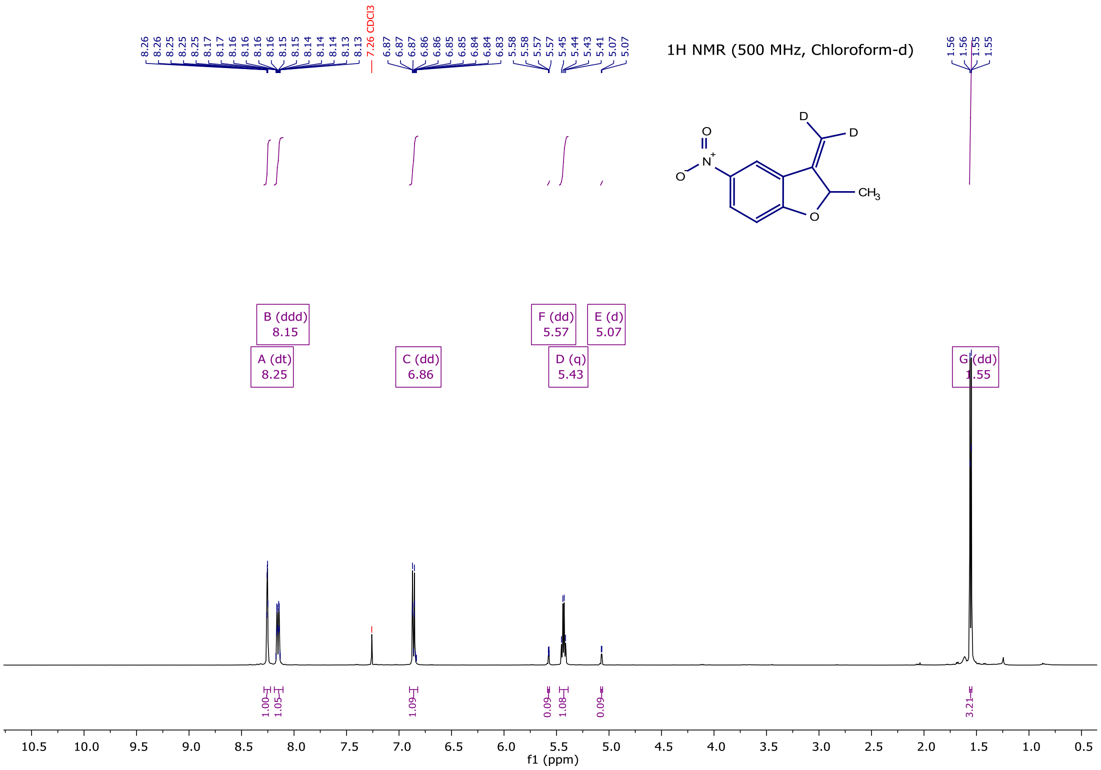

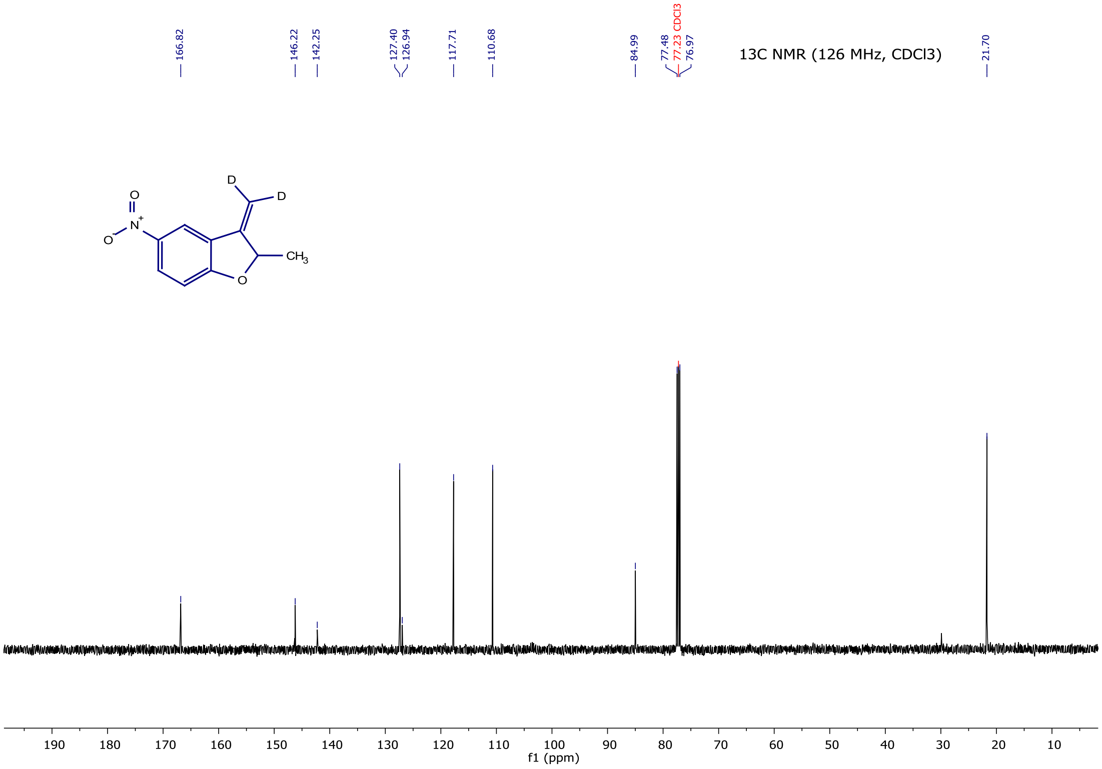
166.82
146.22
142.25
127.40
126.94
117.71
110.68
84.99
77.48
77.23 CDCl3
76.97
13C NMR (126 MHz, CDCl3)
21.70
D
D
O
N+
O-
CH3
O
190 180 170 160 150 140 130 120 110 100 90 80 70 60 50 40 30 20 10
f1 (ppm)

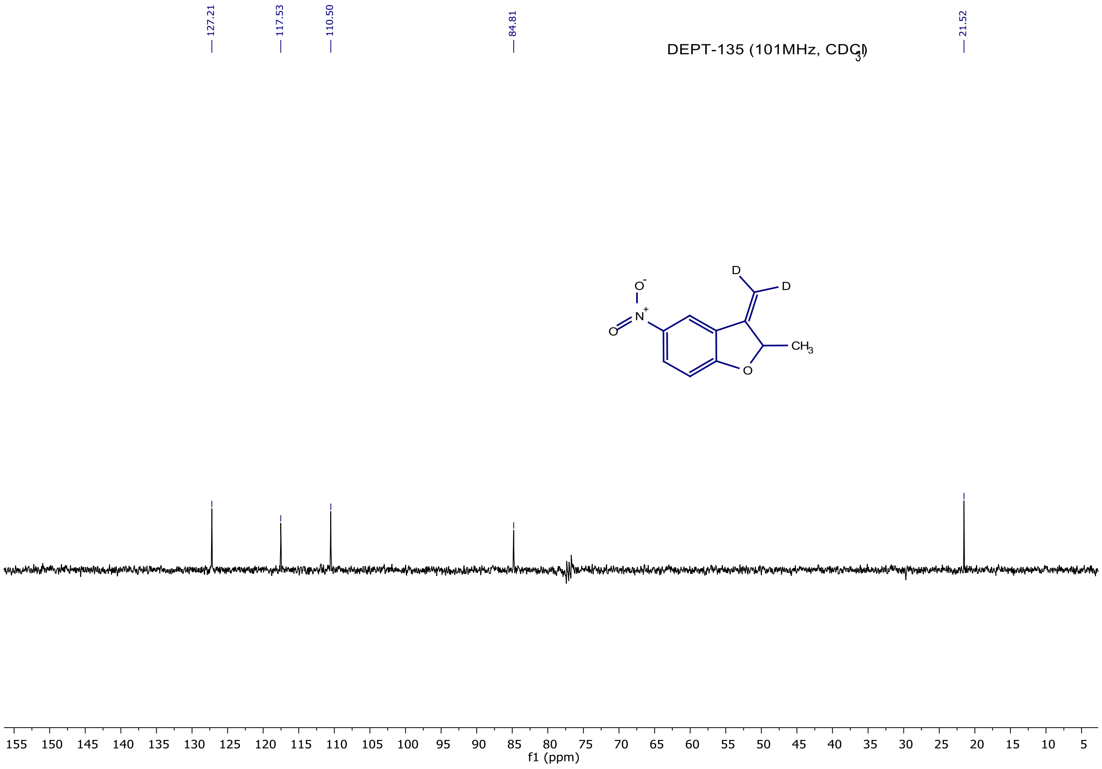
127.21
117.53
110.50
84.81
DEPT-135 (101MHz, CDCl3)
21.52
D
D
O-
N+
O
CH3
O
155 150 145 140 135 130 125 120 115 110 105 100 95 90 85 80 75 70 65 60 55 50 45 40 35 30 25 20 15 10 5
f1 (ppm)

Scheme 4, Entry 4'a:

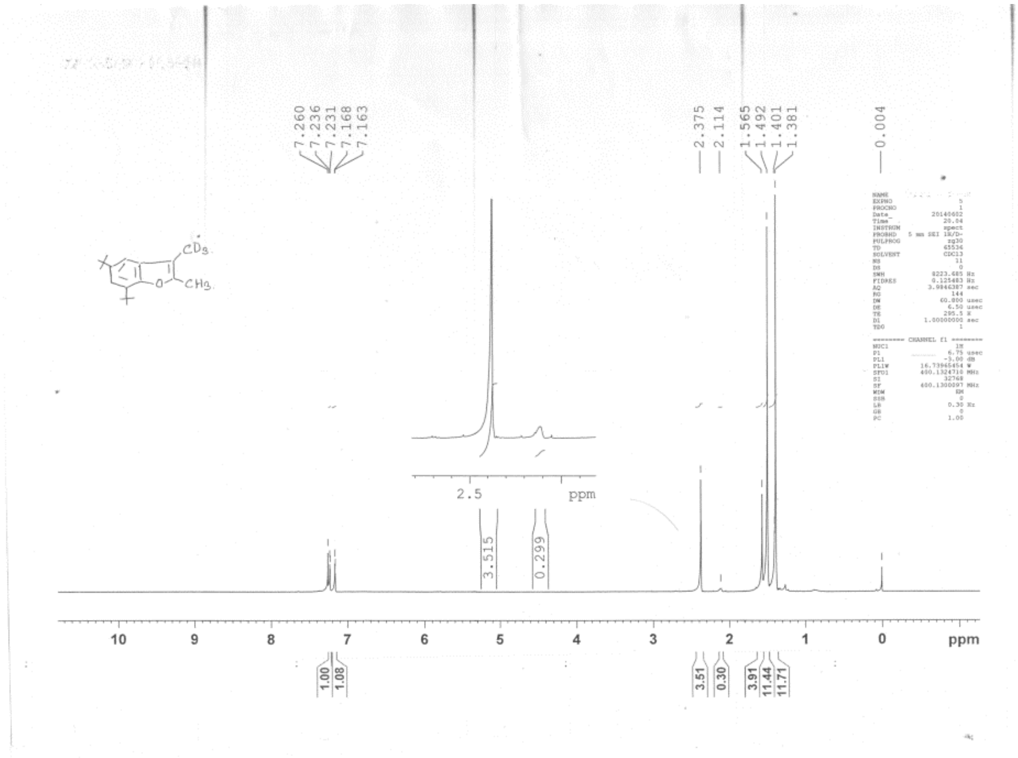

Scheme 4, Entry 4'b:

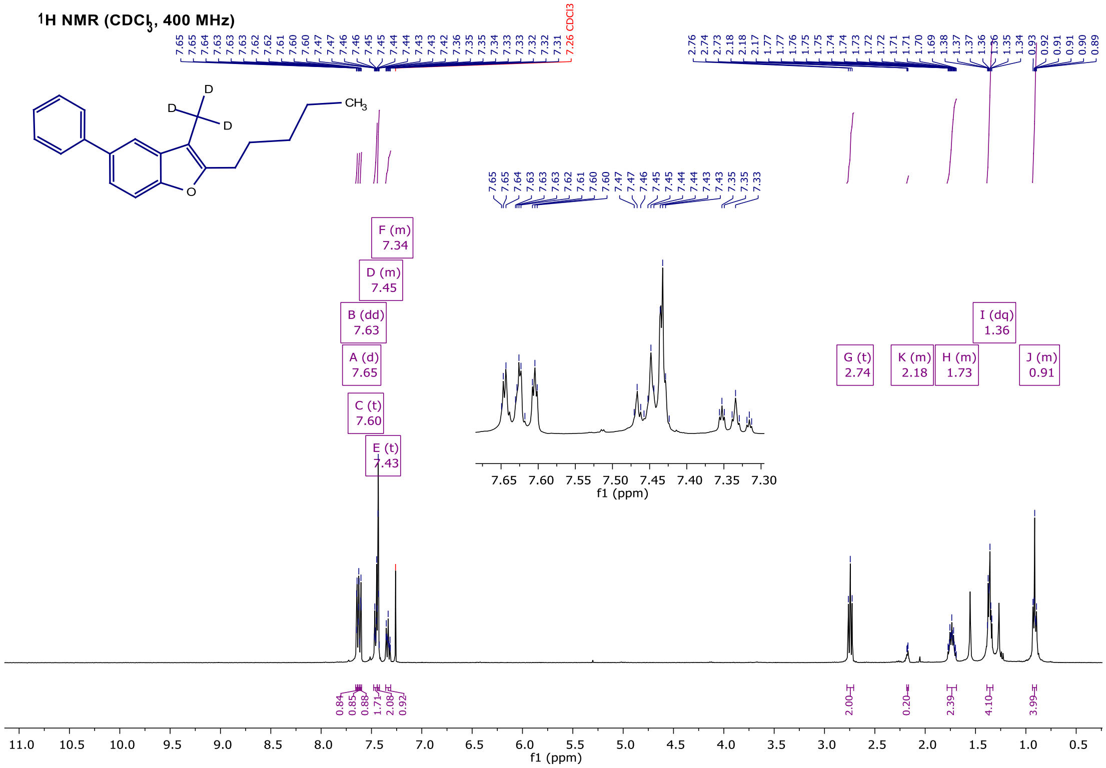

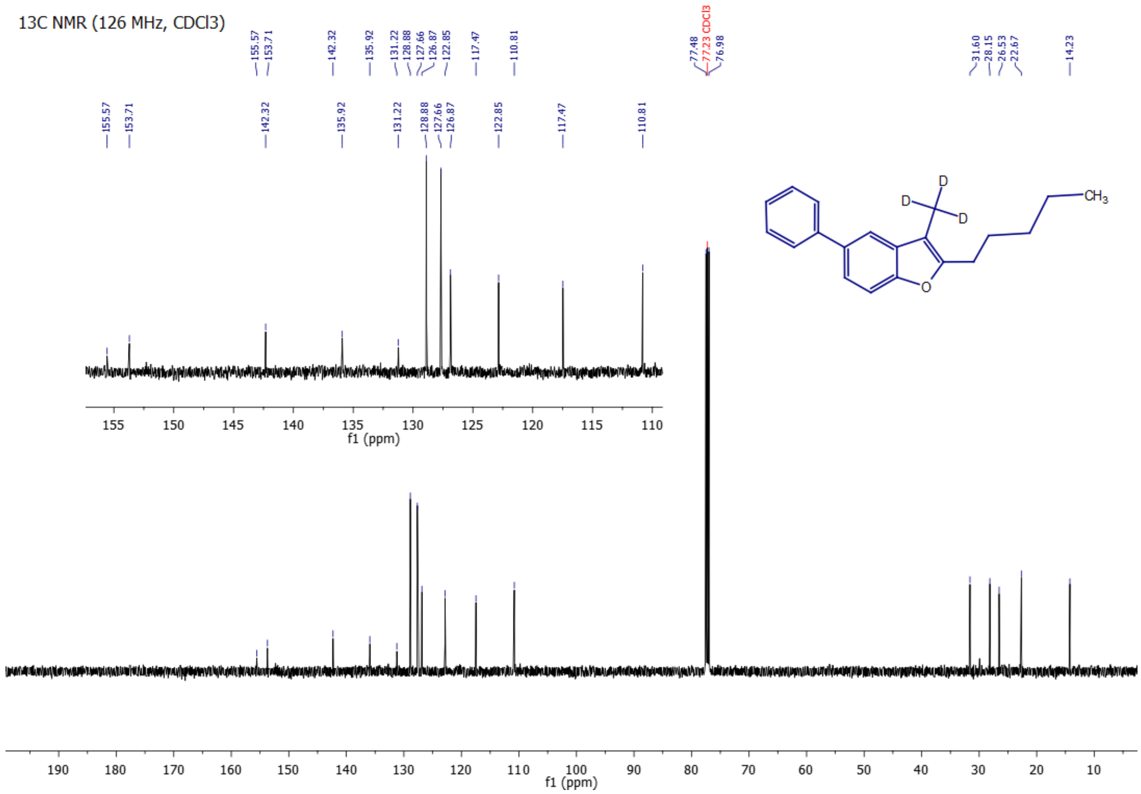
13C NMR (126 MHz, CDCl3)

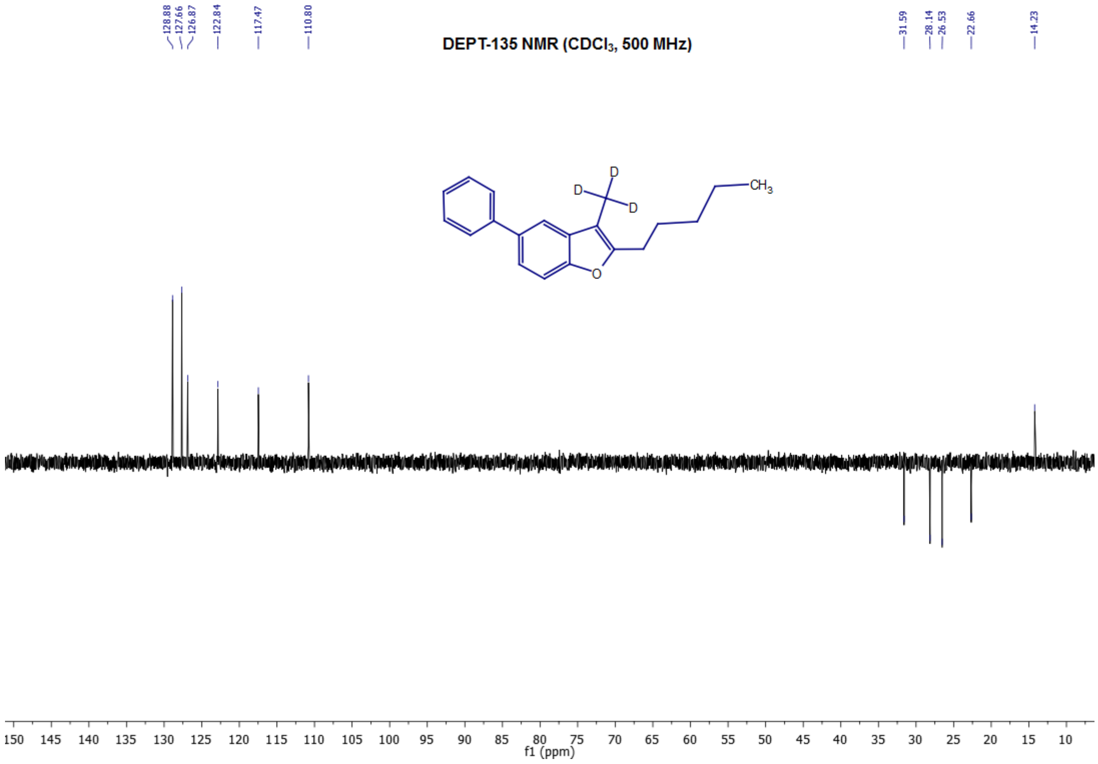
DEPT-135 NMR (CDCl3, 500 MHz)

Scheme 4, Entry 4'c:

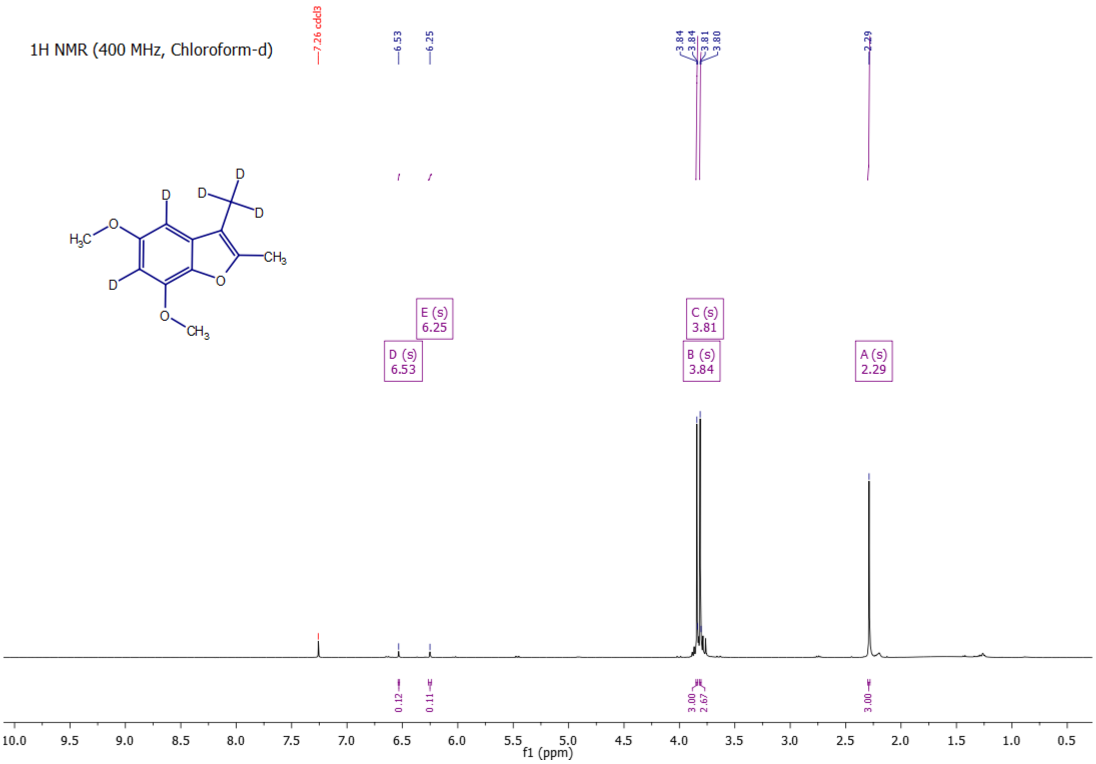

Scheme 4, Entry 6'a:

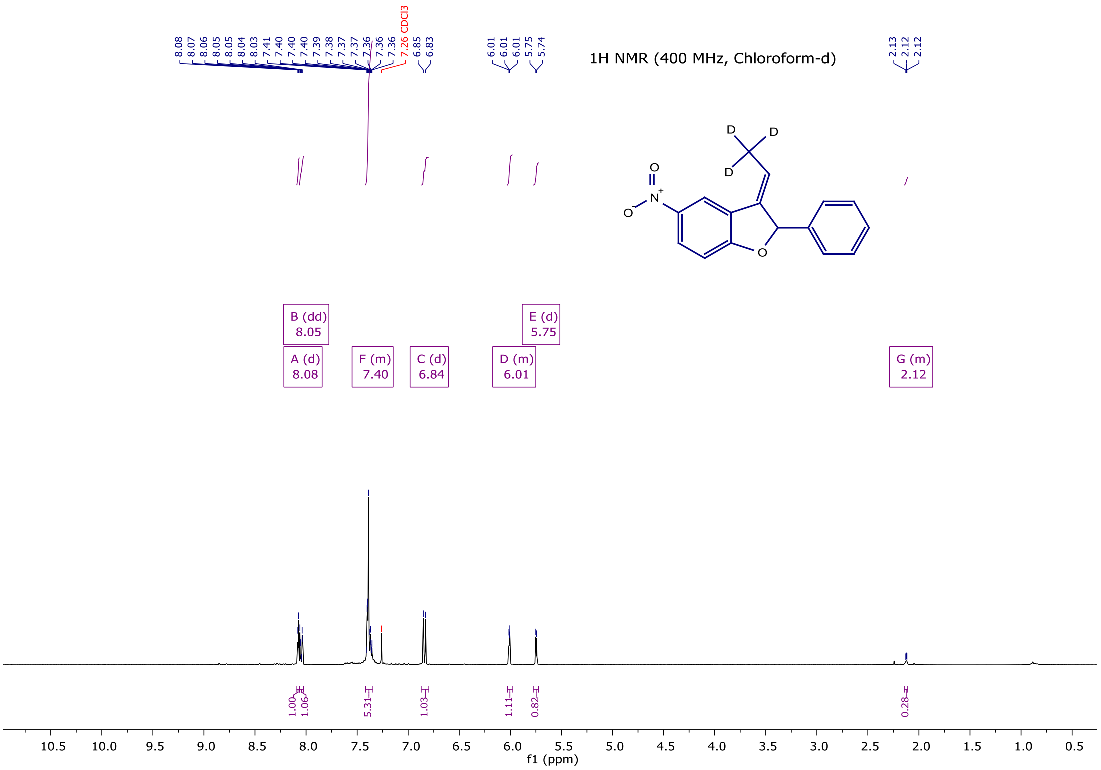

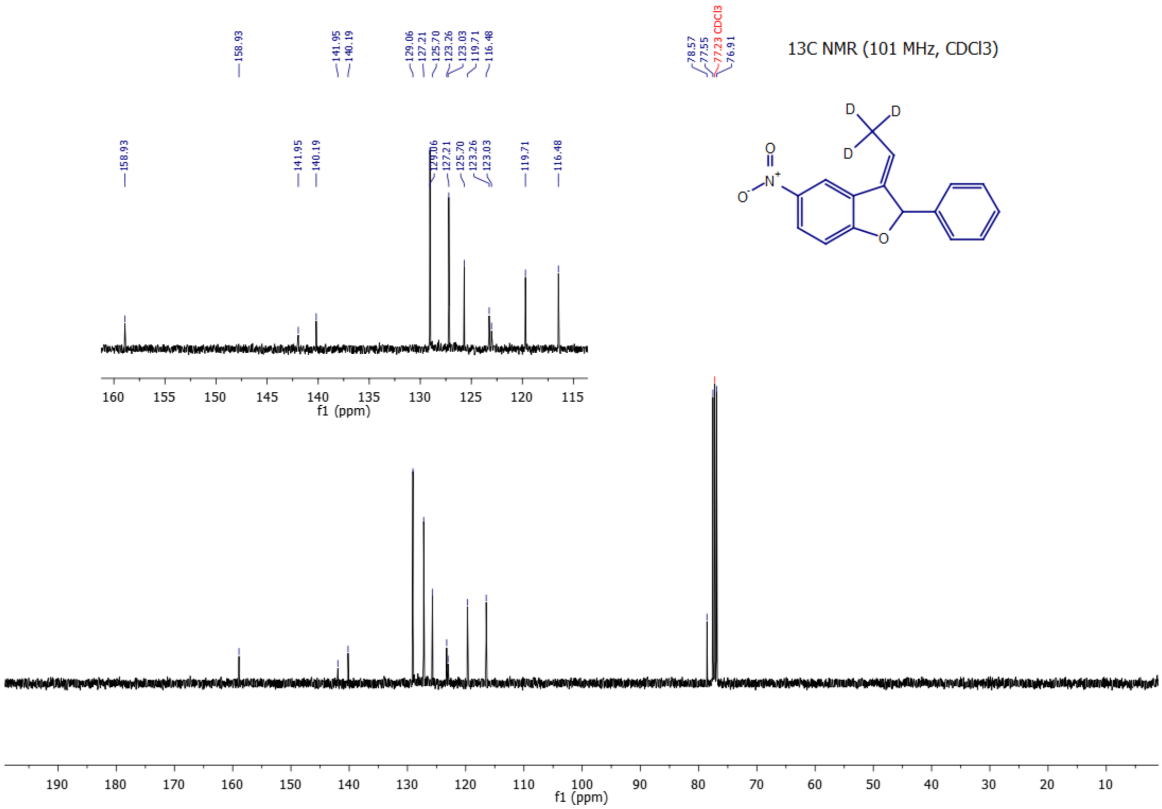
13C NMR (101 MHz, CDCl3)
158.93
141.95
140.19
129.06
127.21
125.70
123.26
123.03
119.71
116.48
78.57
77.55
77.23 CDCl3
76.91
f1 (ppm)

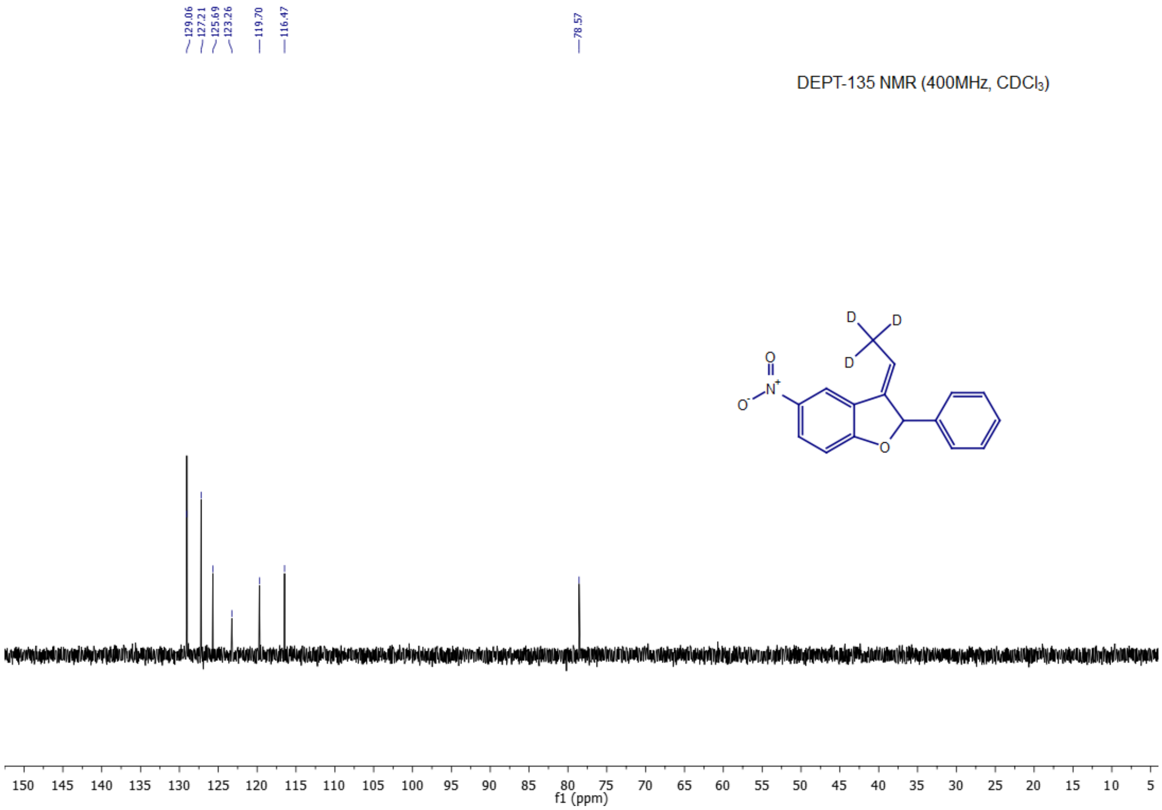
DEPT-135 NMR (400MHz, CDCl3)
129.06
127.21
125.69
123.26
119.70
116.47
78.57
f1 (ppm)

Scheme 4, Entry 6'b:

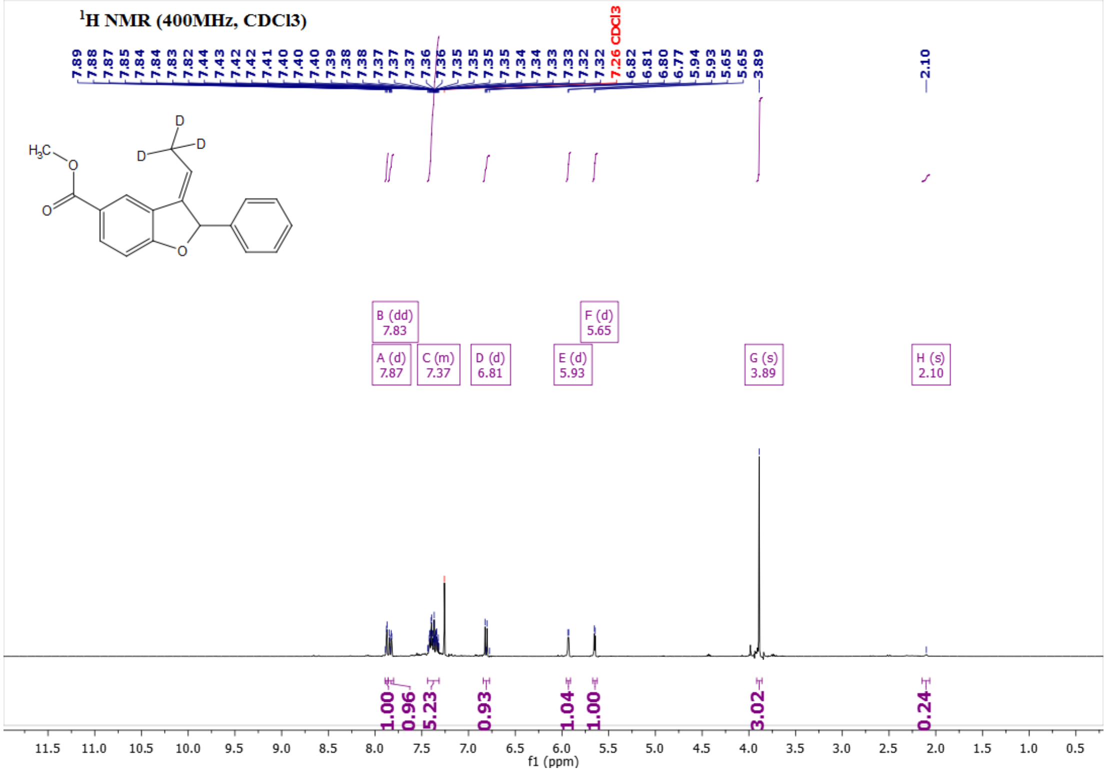

Scheme 4, Entry 6'c:

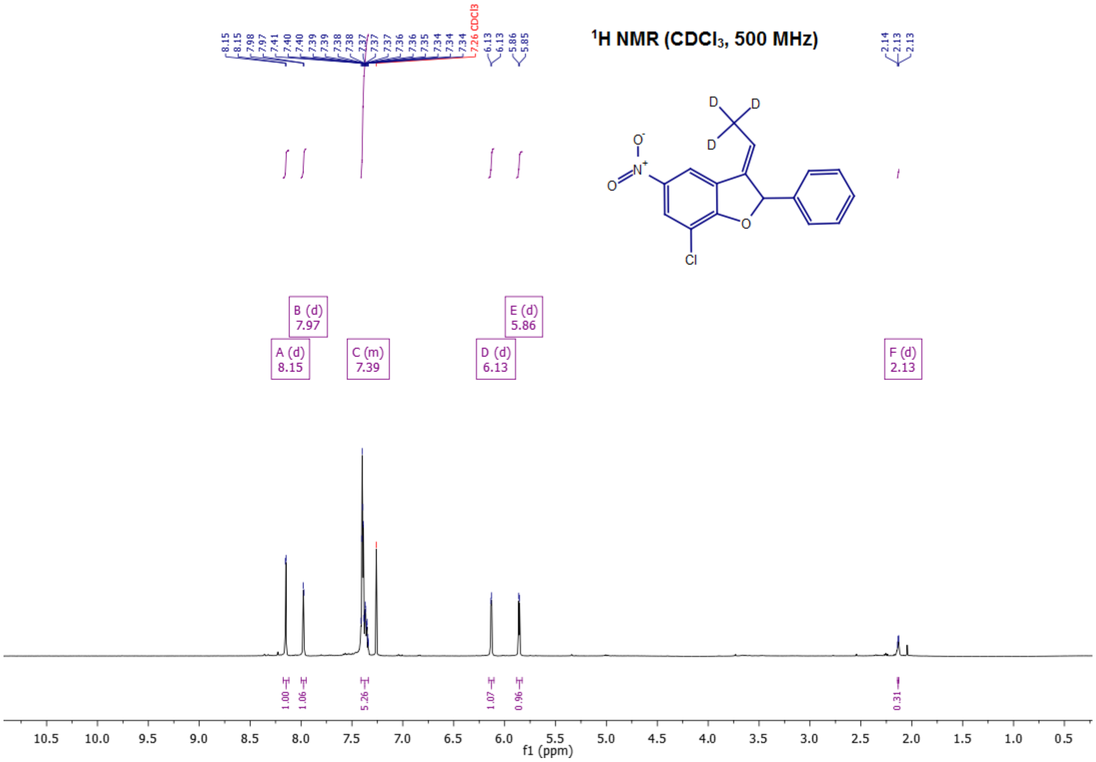

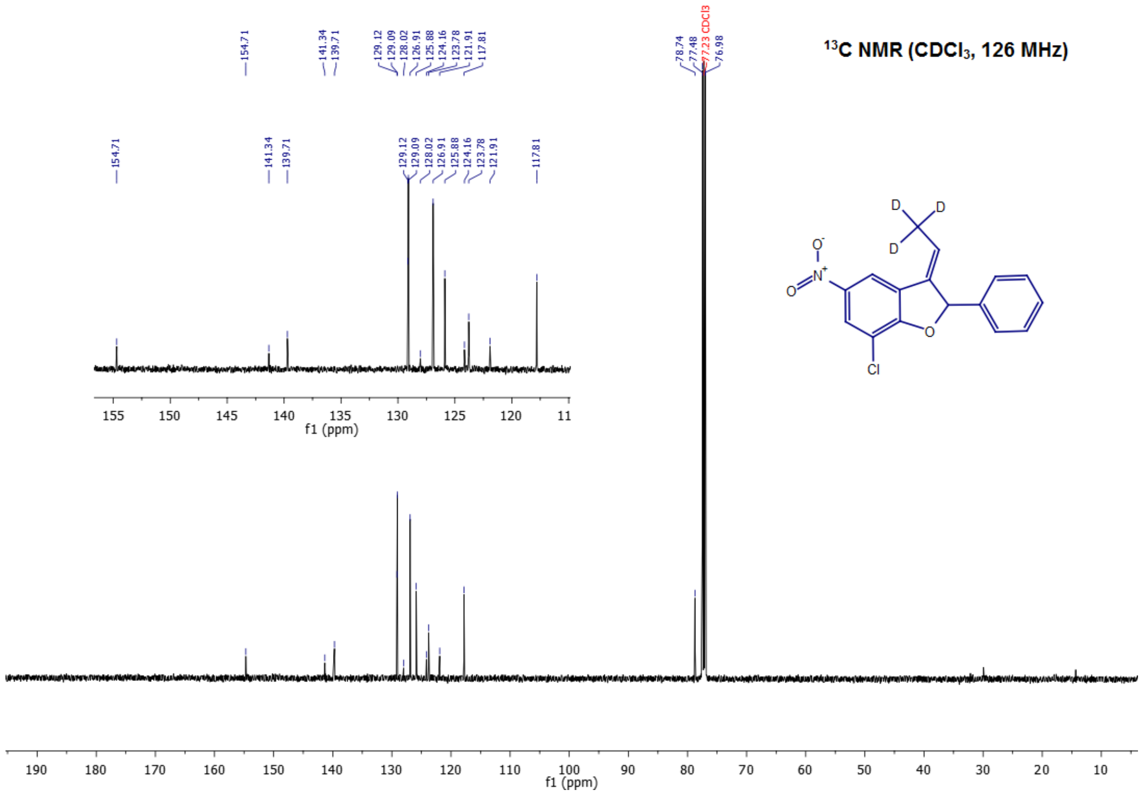
13C NMR (CDCl3, 126 MHz)
154.71
141.34
139.71
129.12
129.09
128.02
126.91
125.88
124.16
123.78
121.91
117.81
78.74
77.48
77.23 CDCl3
76.98
f1 (ppm)
D
D
D
O
N+
O
O
Cl

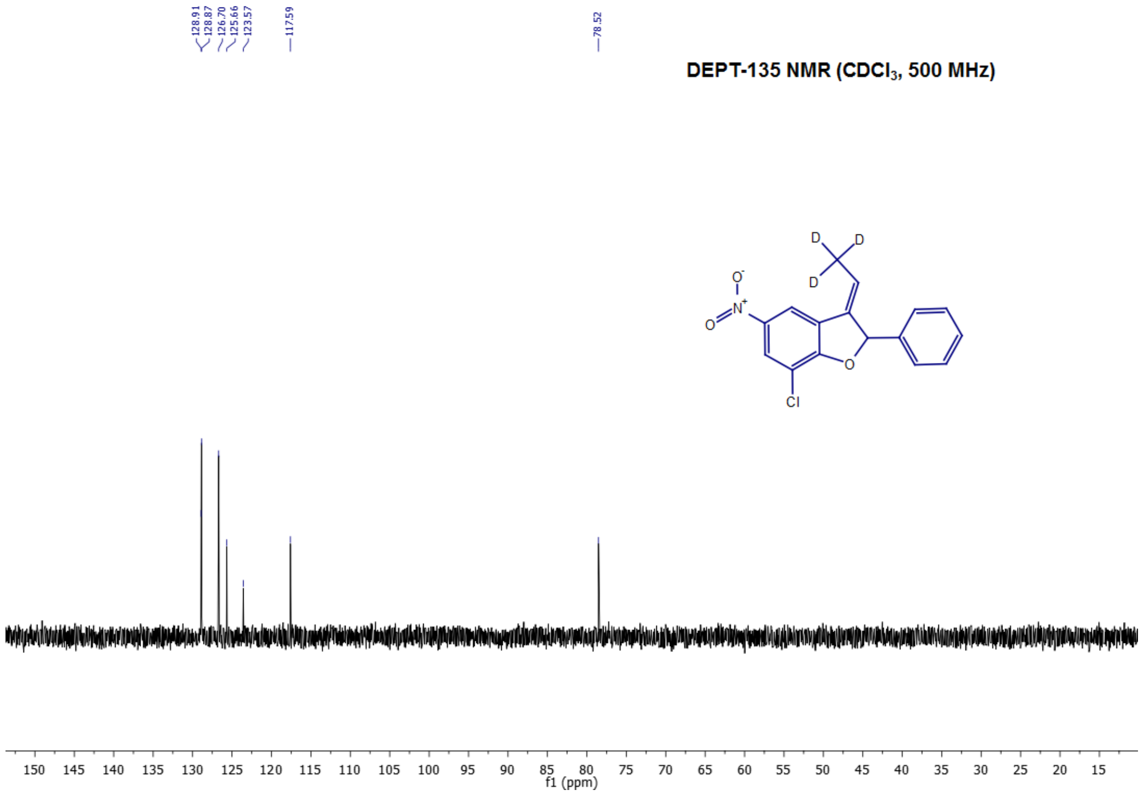
DEPT-135 NMR (CDCl3, 500 MHz)
128.91
128.87
126.70
125.66
123.57
117.59
78.52
f1 (ppm)
D
D
D
O
N+
O
O
Cl

[d$_5$]PhOH experiment (Scheme 5):

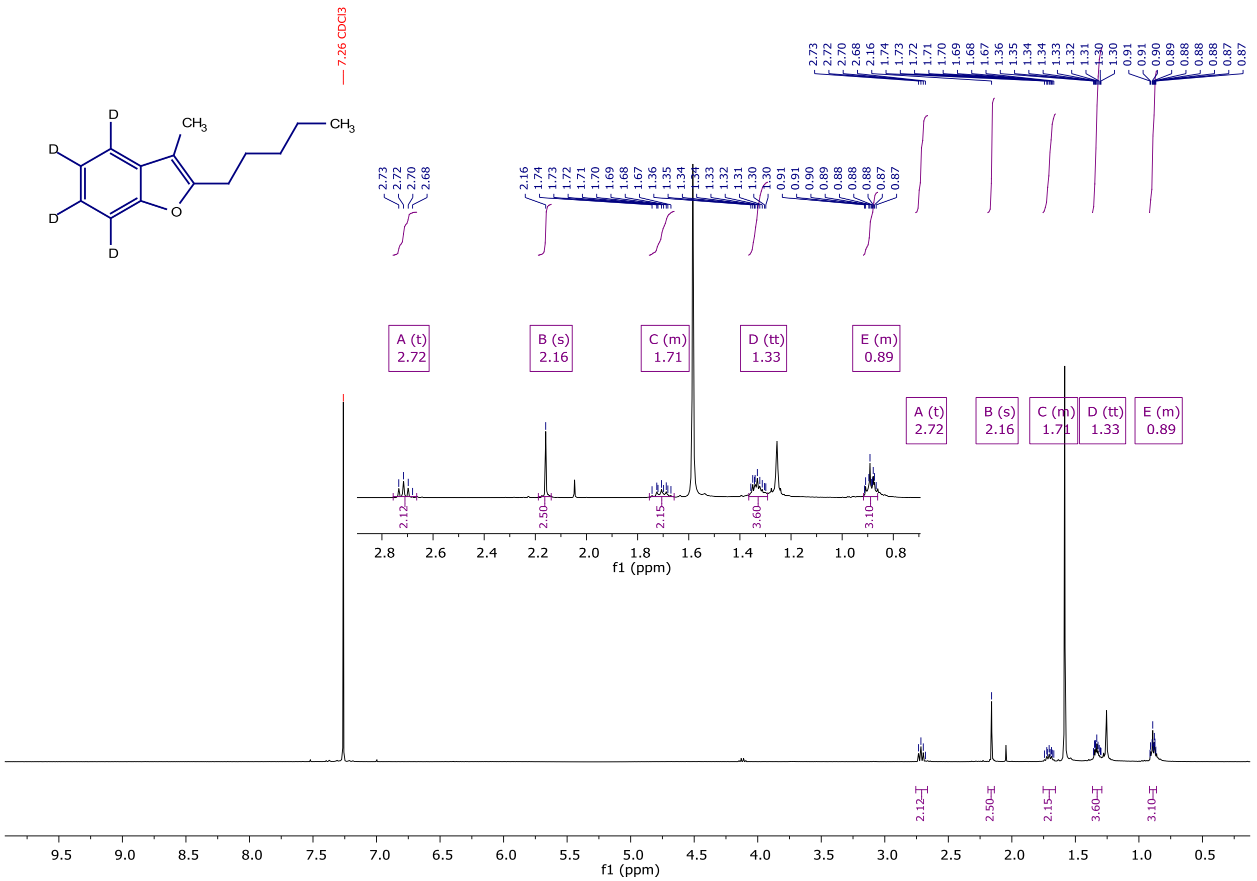

……………………..
